# MHC variation sculpts individualized microbial communities that control susceptibility to enteric infection

**DOI:** 10.1038/ncomms9642

**Published:** 2015-10-23

**Authors:** Jason L. Kubinak, W. Zac Stephens, Ray Soto, Charisse Petersen, Tyson Chiaro, Lasha Gogokhia, Rickesha Bell, Nadim J. Ajami, Joseph F. Petrosino, Linda Morrison, Wayne K. Potts, Peter E. Jensen, Ryan M. O’Connell, June L. Round

**Affiliations:** 1 Department of Pathology, Division of Microbiology and Immunology, University of Utah School of Medicine, Salt Lake City, Utah 84112, USA; 2 Department of Molecular Virology and Microbiology, Baylor College of Medicine, Houston, Texas 77030, USA; 3 Department of Molecular Virology and Microbiology, Alkek Center for Metagenomics and Microbiome Research, Baylor College of Medicine, Houston, Texas 77030, USA; 4 Department of Biology, University of Utah, Salt Lake City, Utah 84112, USA

## Abstract

The presentation of protein antigens on the cell surface by major histocompatibility complex (MHC) molecules coordinates vertebrate adaptive immune responses, thereby mediating susceptibility to a variety of autoimmune and infectious diseases. The composition of symbiotic microbial communities (the microbiota) is influenced by host immunity and can have a profound impact on host physiology. Here we use an MHC congenic mouse model to test the hypothesis that genetic variation at MHC genes among individuals mediates susceptibility to disease by controlling microbiota composition. We find that MHC genotype significantly influences antibody responses against commensals in the gut, and that these responses are correlated with the establishment of unique microbial communities. Transplantation experiments in germfree mice indicate that MHC-mediated differences in microbiota composition are sufficient to explain susceptibility to enteric infection. Our findings indicate that MHC polymorphisms contribute to defining an individual’s unique microbial fingerprint that influences health.

## Abstract (web summary)

Composition of the gut microbiota is regulated by IgA antibodies which are produced under the control of MHCII-restricted B cells. Here the authors show that MHCII polymorphisms sculpt bacterial composition of the gut, which influences a host’s susceptibility to enteric *Salmonella* infection.

## Graphical abstract

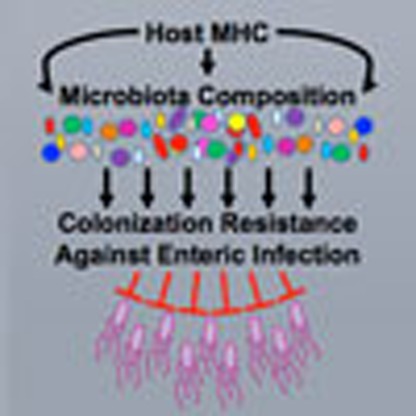

Classical major histocompatibility complex (*MHC*) genes encode cell-surface glycoproteins that form the basis of antigenic self versus non-self discrimination^1^,^2^ by presenting protein antigens (peptides) to circulating T lymphocytes^3^,^4^,^5^. Functionally, recognition of self peptides presented by MHC to T cells results in a tolerant immune response, while inflammation is mounted towards non-self antigens. *MHC* genes are also some of the most polymorphic loci found in vertebrates^6^, and alleles have been linked to most known infectious and autoimmune diseases of man^7^. The central role MHC molecules play in vertebrate adaptive immunity has led to intense research spanning several decades on the functional significance of their extreme diversity.

The physiological relevance of MHC polymorphisms has classically been appreciated from the perspective of host-pathogen interactions, where certain MHC alleles bias susceptibility to infection by virtue of their ability to present different pathogenic epitopes. However, in contrast to the transient nature of most infections, individuals are colonized from birth with their microbiota, which is known to have a pervasive influence on host physiology^8^. Studies in knockout mouse models have shown that immune-mediated dysregulation of microbiota composition is a predisposing factor for multiple diseases^9^,^10^,^11^,^12^,^13^,^14^,^15^. In addition, multiple studies in mice^16^, rats^17^, fish^18^ and humans^19^,^20^,^21^ have demonstrated correlations between MHC variation and microbiota composition, though the physiologic relevance of these relationships were not determined. Together, these observations suggest that an individual’s MHC genotype might exert its most profound effect on host fitness by influencing the relationship between hosts and their symbiotic microbiota. Whether MHC genotype impacts host health by functioning to sculpt an individual’s microbiota has not been tested.

Antibody-mediated (that is, humoral) immunity is facilitated in the gut by interactions between MHC class II restricted CD4^+^ T-follicular (T_FH_) helper cells and naive B cells that instigate germinal centre formation and the production of high-affinity immunoglobulin A (IgA). IgA controls the abundance of extracellular microbes by tagging organisms for destruction by the immune system, by regulating bacterial epitope expression^22^, and by aggregating and eliminating them from the gut via peristalsis. Thus, antibody-mediated selection is a key means by which hosts are capable of controlling microbial community composition in the gut. In support of this, activation-induced cytidine deaminase (AID)-deficient animals (whose B cells do not undergo somatic hypermutation and affinity maturation) have severe alterations to their gut microbiota^23^. In addition, defects in the interaction between T_FH_ cells and germinal centre B cells alters the host IgA antibody repertoire, which is associated with differences in the community of organisms that develop within these animals^15^,^24^. Given the role of MHC class II molecules in driving humoral immune responses, this is a likely mechanism through which MHC polymorphisms could shape microbiota composition.

Previous research has demonstrated differential patterns of susceptibility among MHC congenic mouse strains against a wide variety of enteric pathogens^25^,^26^. This is generally assumed to reflect variability in an individuals’ suite of MHC molecules that differentially stimulate the immune system to clear infection and limit disease. However, differences in the composition of resident microbial communities can influence disease susceptibility associated with pathogenic infection. Colonization resistance is a phenomenon that occurs when members of the microbiota inhibit the establishment of environmentally acquired pathogens, thus limiting their potential to infect and cause disease. Moreover, specific members of a microbiota are more important than others in conferring colonization resistance^27^,^28^. Based on this, we tested the hypothesis that MHC polymorphisms could dictate susceptibility to enteric infection and its associated disease by influencing microbial community architecture.

Results from our experiments demonstrate that MHC polymorphisms influence gut mucosal immunity by driving differential IgA responses that develop against commensal microbes. MHC-mediated differences in gut immunity were correlated with the establishment of unique microbiota communities among individuals. Importantly, microbiota transplant experiments in germfree mice demonstrated that the unique microbiotas formed in mice of different MHC genotypes impacted host health by controlling susceptibility to enteric infection independent of the immune response. In addition, microbiota from an MHC heterozygous genotype conferred resistance to infection similarly to the microbiota derived from the most resistant MHC homozygous genotype. Thus, results from our experiments indicate that MHC-mediated patterns of disease susceptibility, including heterozygote advantage, may partially be explained by how MHC sculpts microbiota composition in the gut. This study also establishes *MHC* genes as primary host immunogenetic factors driving the high degree of individuality in microbiota composition observed among humans.

## Results

### MHC polymorphisms control gut IgA phenotypes

**Figure 1:**
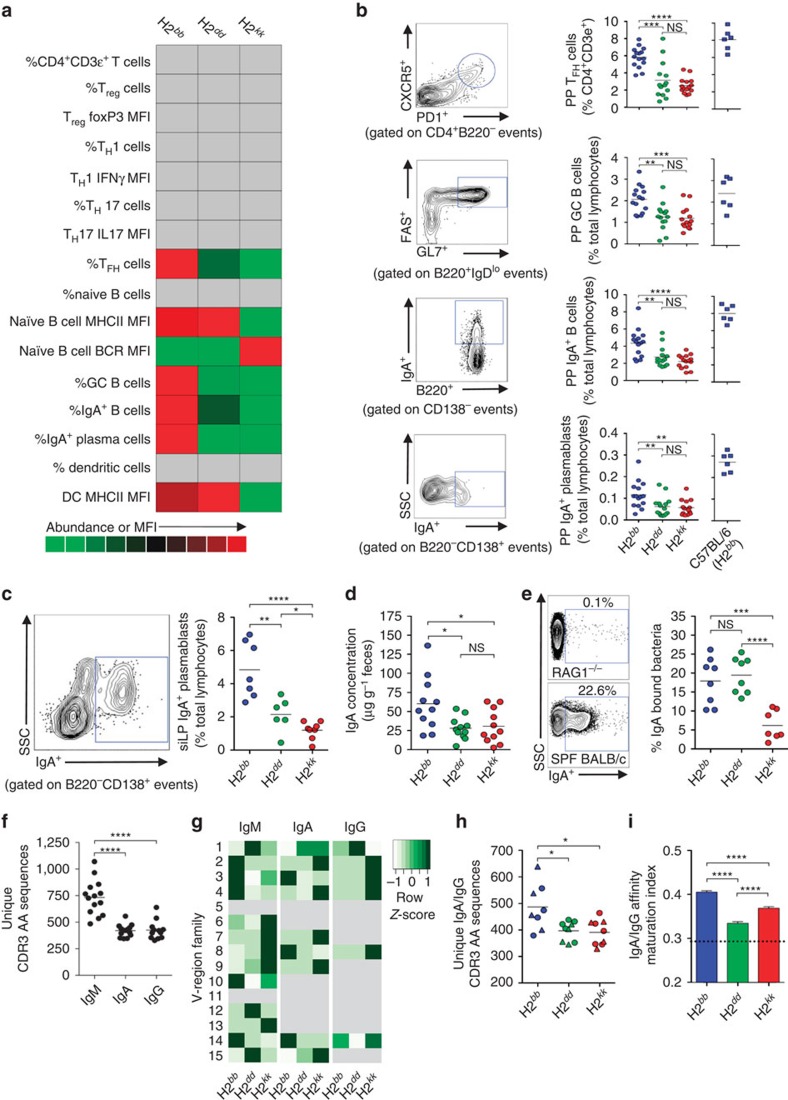
MHC mediates IgA response against commensals. (**a**) Flow cytometry (F.C.) was used to quantify multiple immune parameters within the PPs of MHC congenic animals. A heatmap is used to summarize results of immune phenotyping experiments. Heatmap depicting relative abundances of cell subsets (red=more abundant, green=less abundant). Gray boxes represent no significant differences among genotypes for a given cell subset based on the results of a one-way ANOVA (*P*>0.05). (**b**) Data sets comparing discriminating immune phenotypes (H2^*bb*^ *n*=15; H2^*dd*^ *n*=14; H2^*kk*^ *n*=15). Data represent pooled results of three replicate experiments. Data from H2-matched C57BL/6 (H2^*bb*^) mice (*n*=6) are provided for comparison against H2^*bb*^ BALB/c congenics. (**c**) Abundance of IgA^+^ plasmablasts in the siLP was enumerated via flow cytometry (H2^*bb*^ *n*=7; H2^*dd*^ *n*=6; H2^*kk*^ *n*=8). (**b**,**c**) Representative F.C. plots are provided to illustrate gating strategy. (**d**) The abundance of faecal IgA antibodies was measured by ELISA (H2^*bb*^ *n*=11; H2^*dd*^ *n*=11; H2^*kk*^ *n*=11). Data represent pooled results of two replicate experiments. (**e**) The relative amounts of faecal bacteria bound by IgA was enumerated via flow cytometry among cohorts of H2^*bb*^ (*n*=8), H2^*dd*^ (*n*=8), and H2^*kk*^ (*n*=7) animals. Representative F.C. plots illustrate assay specificity by demonstrating a low incidence of false-positive events recorded in IgA-deficient RAG1^−/−^ control animals. Data represents pooled results from two replicate experiments. (**f**) The number of unique PP-derived CDR3 amino acid sequences (from IgH chain) observed per sample in each immunoglobulin repertoire rarified to a depth of 1,250 sequence observations per sample. Plots represent pooled data from all three genotypes. (**g**) Heatmap showing IgH variable family usage among genotypes. For each repertoire, sequence abundance per variable region family is normalized across row via *Z*-score. Gray cells indicate that a variable family was absent in all three genotypes within a given Ig repertoire. (**h**) The number of unique IgA and IgG CDR3 amino acid sequences (pooled) observed among genotypes rarified to a depth of 1,250 sequence observations per sample (H2^*bb*^ *n*=4; H2^*dd*^ *n*=4; H2^*kk*^ *n*=4). (**i**) The mean affinity maturation index for all unique IgA and IgG sequences within a given genotype. Error bars represent s.e.m. (**b**,**c**,**d**,**e**,**f**,**h**,**i**) Bars represent means. Asterisks denote results of two-tailed unpaired Student’s *t*-tests (****P<0.00001; ***P<0.001; **P<0.01; *P<0.05). NS, not significant.

Humoral immunity is a key means by which hosts regulate microbial composition in the gut in an antigen-specific manner, primarily through production of secretory IgA^15^,^23^,^24^,^29^. *MHC class II* genes are central to this process, so we first sought to determine whether an individual’s MHC genotype influenced steady-state development of the gut immune response. We focused on characterizing immune parameters within the Peyer’s patches of mice as these are the primary inductive sites of T-cell-dependent IgA responses in the gut. MHC (called the H2 complex in mice) congenic mice have been bred to possess unique suites of alleles at *MHC class I* and *class II* genes, while sharing the same genetic background (BALB/c), making them an ideal model to investigate the role of MHC genotype on animal physiology (Supplementary Table 1). Hereafter our model BALB/c H2 congenic mouse strains will be designated as H2^*bb*^, H2^*dd*^ and H2^*kk*^, where superscripts denote homozygous H2 genotypes derived from C57BL/6, BALB/c and C3H/He mice, respectively. Immune phenotyping experiments using flow cytometry revealed significant differences among MHC genotypes in a variety of immune parameters related to the IgA response (Fig. 1a,b). Specifically, compared with animals possessing the H2^*dd*^ and H2^*kk*^ genotypes, congenic mice possessing the H2^*bb*^ region had significantly increased abundance of T_FH_ cells (CD4^+^B220^−^CXCR5^+^PD-1^+^), germinal centre B cells (B220^+^IgD^lo^FAS^+^GL7^+^), IgA^+^ B cells (B220^+^CD138^−^IgA^+^) and IgA^+^ plasmablasts (B220^−^CD138^+^IgA^+^; Fig. 1b). Interestingly, H2^*kk*^ congenic mice had significantly reduced MHC class II surface expression on conventional dendritic cells (CD40^+^CD86^+^CD11c^+^MHCII^+^) as well as on naive B cells (B220^+^IgD^hi^MHCII^+^) compared with H2^*dd*^ and H2^*bb*^ mice (Supplementary Fig. 1a). Finally, epistatic interactions between MHC polymorphisms and *non-H2* genes in the BALB/c genetic background could potentially explain the observed differences in immune phenotypes between mice possessing the natural H2^*dd*^ region and congenic animals possessing the recombined H2^*bb*^ and H2^*kk*^ regions. To address this, we compared the same set of immune parameters in C57BL/6 and C3H/He mouse strains that were H2-matched with the H2^*bb*^ and H2^*kk*^ congenic strains, respectively. Parallel effects were observed between H2-matched genotypes for all the observed phenotypes. Similar to H2^*bb*^ congenic BALB/c mice, C57BL/6 (H2^*bb*^) had higher levels of T_FH_, GC B cells, IgA^+^ B cells and IgA^+^ plasmablasts compared with the H2^*dd*^ genotype (Fig. 1b), and C3H/He (H2^*kk*^) animals had similarly reduced surface expression of MHC on conventional dendritic cells and naive B cells (Supplementary Fig. 1a). These data strongly support that observed phenotypic differences among BALB/c H2 congenic mice are a result of MHC-intrinsic effects, and demonstrate that variability in *MHC* genes leads to unique steady-state immune development in the gut.

### MHC controls IgA responses generated against commensals

IgA^+^ B cells and plasmablasts migrate to the small intestinal lamina propria for final maturation into Ig-producing plasma cells, where secreted antibody is then transported and deposited onto the luminal surface of the gut epithelium. Consistent with data from Peyer’s patches, we found that H2^*bb*^ animals had a significantly higher abundance of IgA^+^ plasmablasts in their small intestinal lamina propria compared with the other two genotypes (Fig. 1c). This was associated with significantly greater amounts of IgA in the faeces of H2^*bb*^ animals (Fig. 1d). To ascertain whether differences in humoral immune responses resulted in a quantifiable difference in antibody binding to commensal bacteria, we employed a flow cytometry-based assay. A significantly greater fraction of commensal bacteria were bound to IgA in both H2^*bb*^ and H2^*dd*^ animals compared with H2^*kk*^ animals (Fig. 1e). This is not due to differences in bacterial abundance as faecal bacterial loads are equivalent among genotypes (Supplementary Fig. 1b).

### MHC controls antibody repertoire selection in the gut

Selection on B cells to differentiate into high-affinity antibody-producing plasma cells is MHC restricted^30^,^31^. To address whether MHC polymorphisms influenced the quality of the antibody response against commensals by influencing the nature of selection on the antibody repertoire, we performed Illumina sequencing to characterize developing antibody repertoires in the Peyer’s patches of H2 congenic animals. Somatic recombination produces a naive Ig repertoire with the potential capacity to recognize any epitope. However, during T-cell-dependent Ig responses, signals from T cells instruct selected B-cell clones to expand and undergo the process of affinity maturation and class-switch recombination. Therefore, only a fraction of the diversity within the naive antibody repertoire should be represented in the mature class-switched antibodies. Consistent with this process of negative selection, we observed a significant reduction in sequence diversity in the mature IgA^+^ and IgG^+^ repertoires compared with the naive IgM^+^ repertoire (Fig. 1f). In addition, differences in V-region gene usage were also observed among H2 genotypes in all three Ig repertoires (Fig. 1g), consistent with an influence of MHC polymorphisms on selection of the developing Ig repertoires. Moreover, consistent with the stronger humoral response seen in H2^*bb*^ animals, this genotype is also associated with a significant increase in the accumulation of novel class-switched IgA and IgG sequences (Fig. 1h) and has the highest IgA/IgG affinity maturation scores of the three genotypes (Fig. 1i). Importantly, no differences in repertoire diversity or affinity maturation are observed among H2 genotypes when naive IgM repertoires are compared (Supplementary Fig. 1c,d), which further supports the argument that observed differences arise as a consequence of differential MHC-mediated selection on maturing IgA and IgG antibody repertoires. Collectively, results from our phenotyping, IgA-binding and Ig repertoire experiments indicate that MHC polymorphisms lead to an individualized antibody response that develops in the gut towards commensals.

### MHC sculpts unique microbiota communities

**Figure 2:**
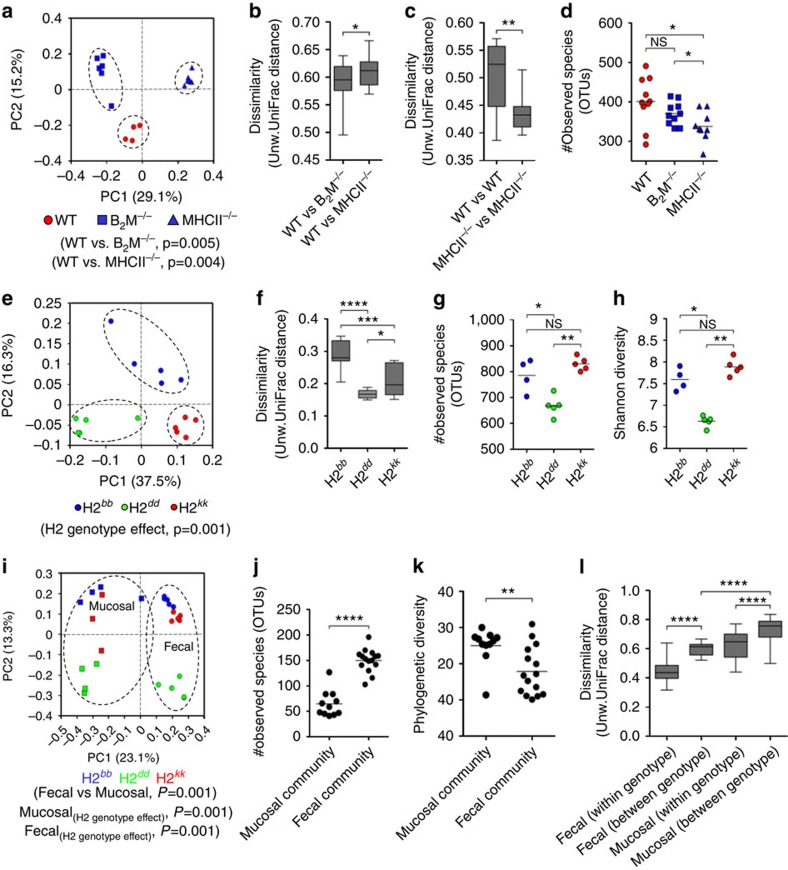
MHC influences microbial composition in the gut. (**a**) PcoA plot based on unweighted UniFrac of faecal communities from male WT (*n*=4), B_2_M^−/−^(*n*=6), and MHCII^−/−^(*n*=6) C57BL/6 animals. (**b**) Distance boxplots of community similarity between WT animals and each of the two KO mouse strains. (**c**) Distance boxplots of community similarity among WT animals versus similarity among MHCII^−/−^ animals. (**d**) Comparison of the observed number of species in WT, B_2_M^−/−^, and MHCII^−/−^ communities. (male and female animals pooled per genotype (WT *n*=9; B_2_M^−/−^ *n*=10; MHCII^−/−^ *n*=9) (**e**) PcoA plot depicting relationships between faecal communities among female MHC congenic animals (H2^*bb*^=blue dots (*n*=5); H2^*dd*^=green dots (*n*=5); H2^*kk*^=red dots (*n*=5)). (**f**) Distance boxplots of community similarity among individuals within each MHC genotype. (**g**) Comparison of observed number of species among H2 congenics. (**h**) Comparison of Shannon diversity estimates among communities from H2 congenic animals. (**i**) PcoA plot illustrating differences in community composition between faecal and mucosal communities, and among MHC genotypes within each of these communities (H2^*bb*^=blue dots (*n*=5); H2^*dd*^=green dots (*n*=5); H2^*kk*^=red dots (*n*=5))(squares=mucosal samples, circles=faecal samples). (**j**,**k**) Comparison of observed number of species (**j**), Shannon diversity (**k**) between faecal and mucosal communities. (**l**) Distance boxplots based on between-genotype unweighted UniFrac distance estimates reflecting the degree of dissimilarity in community composition between genotypes by site (feces versus Mucosa-associated). (**a**,**e**,**i**) Results of PERMANOVAs for PcoA plots provided under respective plot. (**b**,**c**,**d**,**f**,**g**,**h**,**j**,**k**,**l**) Results of two-tailed unpaired Student’s *t*-test (****P<0.00001; ***P<0.001; **P<0.01; *P<0.05). (**d**,**g**,**h**,**j**,**k**) Bars represent means. (**b**,**c**,**f**,**l**) Boxplots represent medians, the 25 and 75% quartiles, and whiskers represent range. NS, not significant.

The IgA response against commensals has been shown to directly influence microbial community composition in the gut^23^,^24^,^29^,^32^,^33^. Therefore, we next sought to determine whether differences in the immune response between H2 congenic mice translated into alterations in microbiota architecture. We used 16S *ribosomal RNA* (*rRNA*) gene sequencing to characterize the influence of MHC-mediated antigen presentation and MHC polymorphisms on microbial community architecture in the gut. Differences in microbial communities can be quantified based on both presence/absence and relative abundance of OTUs (Operational Taxonomic Units at 97% similarity; approximately species equivalents). We hereafter use the terms ‘composition’ and ‘structure’, respectively, to refer to these different quantifications. Unweighted and weighted UniFrac analyses^34^ reflect the relative importance of host MHC genotype on the composition or structure of the microbiota, respectively. Lack of class I- and class II-mediated antigen presentation (B_2_M^−/−^ and MHCII^−/−^ mice, respectively) was associated with significant shifts in faecal microbial composition and structure compared with wild-type (WT) controls (Fig. 2a, Supplementary Fig. 2a), with significant differences in the relative abundance of a variety of specific genera (Supplementary Fig. 2b). Notably, the genus *Lactobacillus* was significantly reduced in MHCII^−/−^ compared with WT animals, while segmented filamentous bacteria and *Helicobacter* were enriched (Supplementary Fig. 2b). Faecal microbial communities from MHCII^−/−^ mice were significantly more dissimilar to those from WT animals than were communities from B_2_M^−/−^ mice (Fig. 2b), implying that class II antigen presentation plays a stronger role in mediating microbial community composition in the gut. In addition, communities from MHCII^−/−^ animals were more similar to one another (Fig. 2c) and had fewer observed species (Fig. 2d) compared with WT and B_2_M^−/−^ animals, suggesting that class II antigen presentation promotes inter-individual variability in microbiota composition and microbial community diversity, respectively.

We next sought to determine whether MHC polymorphisms lead to distinct microbial communities. To control for effects of isolation among congenic strains (legacy effects), homozygous animals were purchased from Jackson Laboratories and bred to create F_1_ heterozygotes. F_1_ animals were bred and homozygous offspring were identified via genotyping using microsatellite markers that distinguish the respective H2 haplotypes. F_2_ homozygotes were subsequently bred pure and housed under identical conditions in our specific pathogen-free (SPF) facility. Unweighted UniFrac analysis revealed significant divergence of faecal microbial communities among H2 congenic animals (Fig. 2e), while a marginal difference among genotypes was observed based on weighted UniFrac analysis (Supplementary Fig. 2c). Differences among MHC genotypes in constraining community membership were also observed, as comparisons between individuals of the same genotype revealed greater dissimilarity within H2^*bb*^ and H2^*kk*^ communities than within H2^*dd*^ communities (Fig. 2f). Finally, microbial communities from H2^*dd*^ animals were also observed to be less species rich (Fig. 2g) and less diverse (Fig. 2h) than H2^*bb*^ and H2^*kk*^ communities.

IgA antibodies are transported across mucosal epithelium and concentrate within the mucus layer where these molecules provide a first line of antigen-specific defense of underlying host tissues^35^,^36^. Indeed, IgA tends to target organisms present within the mucus^15^. To test whether MHC genotype exerted its strongest effect at this site, we sequenced mucosally associated and faecal communities in MHC congenic mice. In agreement with previously published findings^15^, faecal and mucosally associated communities were significantly different (Fig. 2i). Mucosally associated communities were less species rich (Fig. 2j) and more phylogenetically diverse (Fig. 2k) than faecal communities. These data imply that the mucosa represents an environment with more unique niches relative to faeces that are filled by a more phylogenetically diverse array of species. In addition, while MHC genotype had a significant effect on community composition at both sites (Fig. 2i, Supplementary Fig. 2d), compositional differences were more pronounced when mucosally associated versus faecal communities were compared (Fig. 2l). Thus, MHC polymorphisms are again associated with significant compositional differences among individuals and the effect of MHC genotype is most potent at the site where antibodies are at their highest concentrations; the mucosal surface. Collectively, results from three independent sequencing experiments provide strong support to the growing body of evidence indicating that MHC antigen presentation sculpts microbiota communities and contributes to the high degree of variability in microbiota composition among individuals. Finally, the vast majority of significantly different OTUs across all of our experiments fall within the same six bacterial families from the two dominant phyla found within the vertebrate gut; the Bacteroidetes (families S24-7, Prevotellaceae and Rikenellaceae) and Firmicutes (families Lactobacillaceae, Lachnospiraceae and Ruminococcaceae; Supplementary Fig. 2e, Supplementary Tables 4–6). This is noteworthy because, with the exception of the Rikenellaceae, all of these families of bacteria have previously been shown to either be significantly impacted by defective IgA responses, or to be preferentially targeted by IgA antibodies^15^,^23^,^24^,^29^,^37^,^38^. This further supports our argument that observed microbiota shifts are likely due to MHC-mediated variability in the IgA response generated against commensal microbes.

### MHC mediates susceptibility to enteric Salmonella infection

**Figure 3:**
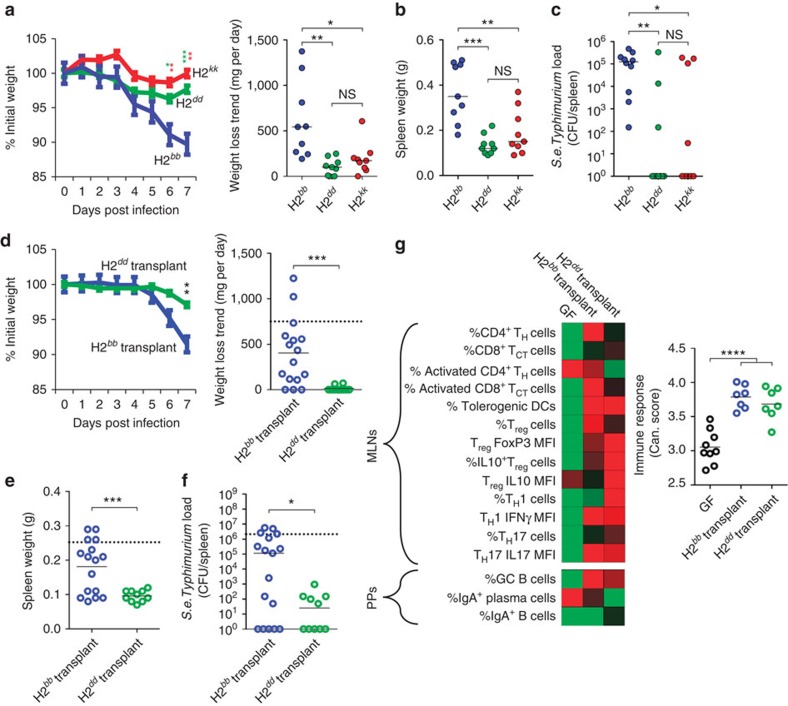
MHC-mediated susceptibility to *S. e. typhimurium* infection is microbiota-dependent. (**a**–**c**) Susceptibility to systemic disease resulting from oral infection with 10^4^ *S. e. typhimurium* CFUs among MHC genotypes (H2^*bb*^ *n*=10; H2^*dd*^ *n*=9; H2^*kk*^ *n*=9). (**a**) Weight loss by day (left panel) and trend in weight loss over time (right panel), (**b**) splenomegaly, and (**c**) *S. e. typhimurium* loads in the spleen on day 7 are shown. Data represents pooled results from two independent experiments. (**d**–**f**) Susceptibility to systemic disease resulting from oral infection with 10^4^ *S. e. typhimurium* CFUs among GF BALB/c animals that had been previously colonized with the microbiotas derived from either H2^*bb*^ (blue open circles (*n*=16)) or H2^*dd*^ (green open circles (*n*=10)) congenic animals. Data represent the pooled results of three independent replicate experiments. Dotted lines represent the mean scores of GF BALB/c animals for each of the respective disease parameters. (**g**) (left panel) Heatmap illustrating how colonization of GF animals with H2^*bb*^ or H2^*dd*^ microbiota influences the gut immune phenotype of animals (GF-*n*=9; H2^*bb*^ transplant *n*=7; H2^*dd*^ transplant *n*=7). (right panel) Results of MANOVA analysis demonstrating that colonization of GF animals results in a significant, but not differential, increase in immune investment by microbiota treatment. MANOVA analysis was based on cell abundance estimated as a percentage. MANOVA (****P<0.00001). Data represents results of two independent experiments. (**a** right panel, **b**,**d** right panel, **e**) Results of two-tailed unpaired Student’s *t*-test (***P<0.001; **P<0.01; *P<0.05). (**a** and **d** (left panels)) Error bars represent s.e.m. Asterisks represent significant differences based on results of *t*-tests comparing weight loss by day and reflect significant differences compared to H2^*bb*^ animals in **a** or GF animals receiving the microbiota of H2^*bb*^ animals in **d**. (**c**,**f**) Asterisks represent significance based on results of Mann–Whitney *U*-Test, (**P<0.01; *P<0.05). NS, not significant.

To investigate whether MHC genotype and the associated microbial communities that develop within these genotypes influence susceptibility to disease, we utilized the *Salmonella enterica typhimurium* (*S. e. typhimurium*) model of enteric infection to which BALB/c mice are susceptible. To first demonstrate that MHC genotype influences susceptibility to *S. e. typhimurium* infection cohorts of H2^*dd*^, H2^*bb*^ and H2^*kk*^ animals were infected orally with 10^4^ *S. e. typhimurium* colony-forming units (CFUs) to model the natural route of infection, and the severity of disease was compared 7 days later. In this model, orally gavaged *S. e. typhimurium* rapidly colonizes the gastrointestinal tract in as little as 1 h post infection, followed by rapid clearance from the faeces (Supplementary Fig. 3a). H2^*bb*^ animals were significantly more susceptible to Salmonella-induced disease than were H2^*dd*^ and H2^*kk*^ BALB/c mice as measured by weight loss, splenomegaly and splenic *S. e. typhimurium* loads (Fig. 3a–c). Importantly, these data establish MHC-mediated patterns of susceptibility to lethal Salmonellosis.

To determine whether the reduced disease severity in H2^*dd*^ and H2^*kk*^ animals was associated with a more robust immune response against *S. e. typhimurium*, we analysed multiple immune parameters via flow cytometry at day 7 post infection. Multiple components of cell-mediated immunity were increased in response to infection, including activated and inflammatory CD4^+^ T_H_ cells (Supplementary Fig. 3b,c). However, this was exclusively driven by animals possessing the susceptible H2^*bb*^ genotype (Supplementary Fig. 3b,c). In addition, while phagocyte uptake is a means by which *S. e. typhimurium* can disseminate to the systemic compartment, there was no significant difference in phagocyte abundance among genotypes (Supplementary Fig. 3d–f). Results from these experiments indicate that resistance to infection in our model is not driven by a more robust immune response and that enhanced disease in H2^*bb*^ animals is not simply a consequence of increased phagocyte trafficking of *S. e. typhimurium* into the systemic compartment.

### Susceptibility to enteric infection is microbiota dependent

To determine whether enhanced resistance to enteric *S. e. typhimurium* infection was influenced by the microbiota, we first treated H2^*dd*^ animals with a cocktail of antibiotics for 3 days to deplete the microbiota and then assessed resulting patterns of susceptibility. Short-term antibiotic treatment, which reduced faecal microbiota loads by an order of magnitude, rendered resistant H2^*dd*^ animals highly susceptible to *Salmonella*-induced disease and systemic invasion (Supplementary Fig. 4a–d). Thus, depletion of the microbiota facilitates systemic invasion and renders an otherwise resistant host genotype highly susceptible to lethal salmonellosis. Previous work has shown that germfree BALB/c animals orally infected with this pathogen quickly succumb to systemic salmonellosis, and that animals can be protected from systemic disease through mono-association with specific bacterial species or colonization by a complex microbiota^39^,^40^. Consistent with these studies, germfree BALB/c animals raised in our facility were highly susceptible to systemic *S. e. typhimurium* infection, whereas exGF BALB/c animals that had been conventionally housed since birth in our SPF facility were protected from disease (Supplementary Fig. 4e–g). Together, these results indicate that the microbiota provide strong colonization resistance against *S. e. typhimurium* infection. Finally, to directly test whether compositional differences in microbial communities are responsible for observed differences in patterns of disease among MHC genotypes, we performed microbial transplantation experiments into GF BALB/c recipients. For these experiments, animals were equally colonized with microbiota stocks derived from animals of the highly resistant H2^*dd*^ or highly susceptible H2^*bb*^ genotypes (Supplementary Fig. 5a–c) and they were subsequently challenged with an oral dose of 10^4^ *S. e. typhimurium* CFUs. Consistent with patterns of disease susceptibility observed between H2^*dd*^ and H2^*bb*^ animals, GF mice colonized with the microbiota derived from H2^*bb*^ animals developed significantly worse disease than those animals colonized with microbiota derived from resistant H2^*dd*^ animals (Fig. 3d–f). Importantly, the results shown in Fig. 3d–f are the pooled results of three independent replicate experiments, and for each replicate experiment a new microbiota stock was created from each H2 genotype. Each replicate experiment showed similar trends in disease susceptibility, which is inconsistent with a cage effect significantly biasing microbiota composition in our model. Collectively, these data demonstrate that the unique microbiotas formed in discrete MHC genotypes are both necessary and sufficient to explain observed patterns of disease susceptibility.

### Microbiotas do not differentially prime gut immunity

The microbiota can promote defense against an invading pathogen by stimulating an immune response^27^. Immune phenotyping experiments in infected MHC congenic animals suggested that resistance to infection in our model is not immune mediated (Supplementary Fig. 3b–f). However, our initial analysis of immune system development in MHC congenic mice demonstrated differences in steady-state levels of several immune parameters (Fig. 1a). Thus, it is possible that colonization with H2^*bb*^ or H2^*dd*^ microbiotas differentially influence the immune response that develops in the gut, which mediates susceptibility to infection. To test this we again performed faecal transplants into GF BALB/c mice and compared the immune phenotypes that emerged in the absence of *Salmonella* infection. Analysis of multiple immune parameters 3 weeks post colonization revealed a significant overall increase in immune response in the gut of colonized animals (Fig. 3g, left panel). Importantly, however, microbiota derived from H2^*bb*^ and H2^*dd*^ animals did not differentially influence the immune response that develops (Fig. 3g, right panel). These data indicate that microbiota-dependent patterns of disease susceptibility shown in Fig. 3d–f are not a consequence of these two microbiotas driving the development of significantly different immune responses in the gut. Thus, microbiota derived from H2^*bb*^ or H2^*dd*^ genotypes recapitulate patterns of disease susceptibility independently of the immune response. In addition, these data also suggest that the differential patterns of immune response observed in MHC congenic animals in Fig. 1 are not due to differences in microbiota composition, but rather are a consequence of host genetics.

### MHC heterozygote advantage is microbiota dependent

**Figure 4:**
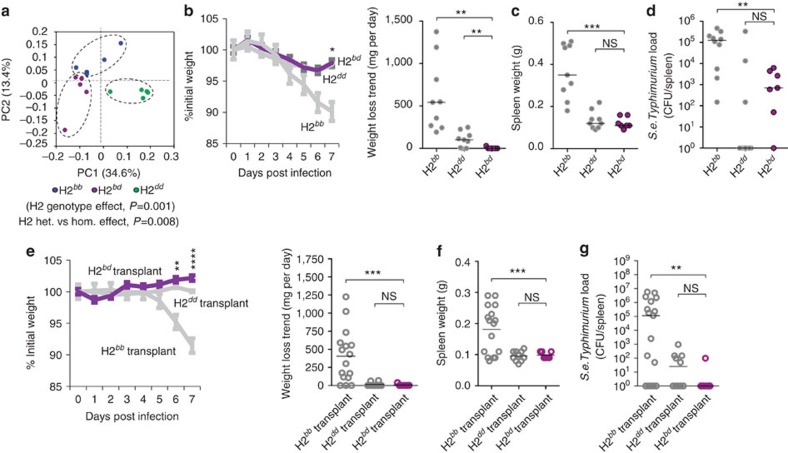
MHC heterozygote advantage is microbiota-dependent. (**a**) PcoA plots highlighting the significant difference in community composition based on unweighted UNIFRAC between H2^*bd*^ (*n*=5) heterozygotes and animals from the respective homozygote H2^*bb*^ (*n*=5) and H2^*dd*^ (*n*=5) genotypes. *P*-value represents result of PERMANOVA significance test. Ellipses highlight relevant comparisons and are non-quantitative. H2^*bb*^ and H2^*dd*^ data points are from the same animals depicted in Fig. 2e–h. (**b**–**d**) Susceptibility to systemic disease resulting from oral infection with 10^4^ *S. e. typhimurium* CFUs among H2^*bd*^ (*n*=7) animals compared to H2^*bb*^ or H2^*dd*^ animals. Data represents pooled results from two replicate experiments. Greyed H2^*bb*^ and H2^*dd*^ data points in b-d are the same as those shown in Fig. 3a–c. (**e**–**g**) Susceptibility to systemic disease resulting from oral infection with 10^4^ *S. e. typhimurium* CFUs among GF BALB/c animals that had been previously colonized with the microbiota from H2^*bd*^ heterozygous animals (*n*=7). Data represents pooled results from two independent experiments. Greyed H2^*bb*^ transplant and H2^*dd*^ transplant data shown in **e**–**g** are the same as those shown in Fig. 3d–f. (**b** right panel, **c**,**e** right panel, **f**) Results of two-tailed unpaired Student’s *t*-test (***P<0.001; **P<0.01). (**b** and **e** (left panels)) Error bars represent S.E.M. Asterisks represent significant differences based on results of *t*-tests comparing weight loss by day and reflect significant differences between H2^*bd*^ heterozygotes compared to H2^*bb*^ animals in **b** or GF animals receiving the microbiota of H2^*bb*^ animals in **e**. (**d**,**g**) Asterisks represent significance based on results of Mann–Whitney *U*-test, (**P<0.01). NS, not significant.

*MHC* genes are highly polymorphic and most individuals possess a unique suite of alleles. Moreover, MHC alleles are co-dominantly expressed. Because of these features it is widely assumed that MHC heterozygosity benefits host health by allowing the immune system to present a wider variety of antigens, thereby enhancing resistance to disease. However, our results demonstrate that MHC polymorphisms lead to unique microbial communities and this can control disease associated with enteric infection. Thus, the advantage conferred by MHC heterozygosity might also be a result of how MHC influences the composition of the microbiota. To test this, we performed 16S *rRNA* gene sequencing to compare microbial communities between female H2^*bd*^ heterozygotes and the two H2^*bb*^ and H2^*dd*^ parental strains used to derive this genotype. While on average H2^*bd*^ heterozygotes do not have more diverse microbial communities compared with homozygotes (Supplementary Fig. 6a,b), MHC heterozygosity had a significant effect on community composition but not structure based on unweighted (Fig. 4a) and weighted (Supplementary Fig. 6c) UniFrac analysis, respectively. These results lend further support for a deterministic role of host MHC in sculpting the composition of microbial communities.

To directly test heterozygote advantage in our model, we performed experimental *S. e. typhimurium* infections in H2^*bd*^ animals and compared resulting patterns of disease susceptibility with that observed in the H2^*dd*^ and H2^*bb*^ parent strains. H2^*bd*^ animals were observed to be similarly resistant to infection as the H2^*dd*^ parent strain (Fig. 4b–d). These data indicate that resistance to *S. e. typhimurium* infection in the H2^*bd*^ heterozygote genotype is dominant, which is consistent with heterozygote advantage in this model. To address whether the microbiota formed in H2^*bd*^ heterozygotes conferred protection against *S. e. typhimurium* infection we again conducted microbial transplantation experiments in GF BALB/c mice. Transfer of the microbiota from H2^*bd*^ donor mice into GF BALB/c recipients completely protected these animals from *S. e. typhimurium* invasion and associated disease (Fig. 4e–g). Therefore, in this model heterozygote advantage operates independently of the effect of heterozygosity on immunocompetence, and instead results from a bias in the composition of the microbiota formed within this genotype. Collectively, results from experiments with MHC heterozygotes indicate that MHC-mediated resistance to *S. e. typhimurium* infection is dominant, which is a commonly observed pattern^25^. Our data indicate that this common pattern, which results in heterozygote advantage over the course of many infections, might be driven by how MHC influences microbiota architecture in addition to its influence on immunocompetence. Interestingly, overall microbiota composition and structure in H2^*bd*^ animals is more similar to H2^*bb*^ than H2^*dd*^ animals (Supplementary Fig. 6d). These data suggest that the presence or relative abundance of a specific species shared between H2^*bd*^ and H2^*dd*^ animals might be the basis of colonization resistance against Salmonella.

### *Lactobacillus spp*. are enriched in resistant animals

**Figure 5:**
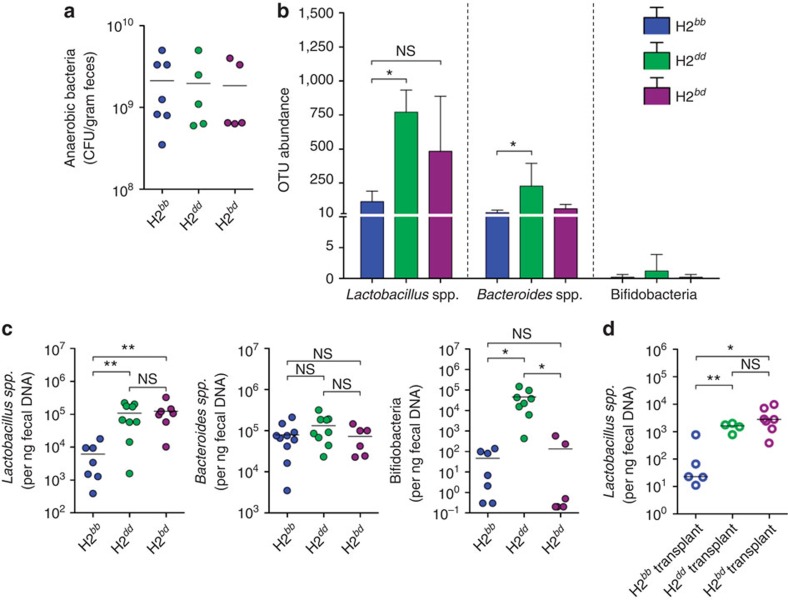
Enrichment of Lactobacillus may explain enhanced colonization resistance. (**a**) Results of experiment quantifying faecal loads of anaerobic bacteria from the faeces of H2^*bb*^, H2^*dd*^ and H2^*bd*^ animals. Loads are standardized to faecal weight (per mg faeces). (**b**) OTU abundance plots of specific bacterial groups from H2^*bb*^ (*n*=5), H2^*dd*^ (*n*=5), and H2^*bd*^ (*n*=5) animals. Asterisks denote significance based on Mann–Whitney *U*-test (*P<0.05). (**c**) Results of qPCR experiment quantifying faecal bacterial loads of Lactobacillus, Bacteroides, and Bifidobacteria in the faeces of H2^*bb*^ (*n*=10), H2^*dd*^ (*n*=9), and H2^*bd*^ (*n*=7) animals. Loads are standardized to the amount of input DNA per reaction and are reported as copies per nanogram faecal DNA. Data represents pooled results from two replicate experiments. (**d**) Results of Q-PCR experiment quantifying faecal bacterial loads of Lactobacillus in the faeces of GF animals that had been previously colonized with the microbiotas of H2^*bb*^ (*n*=5), H2^*dd*^ (*n*=4), or H2^*bd*^ (*n*=7) animals (day 7 of colonization). (**c**,**d**) Asterisk’s denote results of two-tailed unpaired Student’s *t*-test (**P<0.01; *P<0.05). NS, not significant.

Anaerobic bacteria are known to interfere with colonization of the murine gut by *Salmonella*, so enhanced susceptibility to Salmonellosis in H2^*bb*^ animals, or GF animals colonized with H2^*bb*^-derived microbiota, could be due to reduced loads of anaerobic bacteria, or reduced loads of specific groups of anaerobes. Plating of faeces did not reveal a difference in total loads of anaerobic bacteria among these genotypes (Fig. 5a). To determine whether specific anaerobic species previously shown to be antagonistic to *Salmonella* colonization were differentially abundant between H2^*bb*^, H2^*dd*^ and H2^*bd*^ animals, we quantified the relative abundance of species known to be antagonistic to *Salmonella* colonization of the gut. Based on sequencing results, *Lactobacillus spp.* and *Bacteroides spp.* were enriched in H2^*dd*^ animals over H2^*bb*^ animals, but only *Lactobacillus* appeared to be enriched in the H2^*bd*^ genotype as well (Fig. 5b). Quantitative PCR using *Lactobacillus*-specific primers confirmed this result (Fig. 5c). Also, during microbiota transplants significantly less *Lactobacillus* is transplanted into animals colonized with H2^*bb*^-derived microbiota than in animals colonized with H2^*dd*^- and H2^*bd*^-derived microbiota (Fig. 5d). These data imply that enrichment for anaerobes, and specifically *Lactobacillus spp.*, a bacterial group known to be highly antagonistic to *Salmonella* colonization, might account for enhanced colonization resistance against *S. e. typhimurium* in our model.

## Discussion

Colonization resistance is a phenomenon that occurs when members of the microbiota inhibit the establishment of environmentally acquired pathogens, thus limiting their potential to infect and cause disease. Moreover, specific members of a microbiota are more important than others in conferring colonization resistance^27^,^28^. Microbiota transplantation experiments in animal models have been instrumental in defining the relationship between alterations to microbiota composition in the gut and emerging diseases of man including obesity^9^, diabetes^10^, metabolic syndrome^11^ and inflammatory bowel disease (IBD)^12^,^13^,^14^,^15^. Using this methodology, we have provided data to support a model whereby an individuals’ MHC genotype biases IgA-mediated selection against the microbiota that results in compositional differences that impact host health by controlling microbiota-dependent colonization resistance against an enteric pathogen. Importantly, microbial transplants from resistant donors can protect highly susceptible hosts from disease. The relevance of our experimental results to humans is supported by multiple studies. Class II HLA polymorphisms in humans have been correlated with shifts in microbiota composition and diseases like ankylosing spondylitis^19^, rheumatoid arthritis^41^ and coeliac disease^21^. In addition, more recent deep sequencing efforts have revealed strong associations between class II HLA alleles and IgA deficiency^42^ and susceptibility to IBDs like Crohn’s and ulcerative colitis^43^. Importantly, our experiments now identify microbiota-mediated colonization resistance as a malleable resistance mechanism influenced by an individual’s MHC genotype.

Encounters with highly virulent pathogens are rare and autoimmune diseases tend to manifest later in life, so health costs associated with infectious and autoimmune diseases might not be the sole driving force of selection on *MHC* genes. Importantly, results from our experiments provide evidence of reciprocal selection between hosts and their microbiota mediated by *MHC* genes; the hallmark feature of coevolution. Hosts influence the fitness of commensal microbes, and these microbes have a profound influence on host health. Perhaps, the evolution of MHC diversity reflects local co-evolutionary dynamics with commonly encountered environmental microbes whose domestication diversified the metabolic capacity of their vertebrate hosts and limited colonization by more aggressive species. MHC alleles could be maintained over time because they facilitated beneficial symbiotic interactions that outweighed potential risks associated with infectious and autoimmune diseases. In addition, perhaps, antagonistic co-evolution between hosts and rapidly evolving pathogens promoted and maintained alleles that resulted in colonization by less beneficial symbionts, which might explain individual predisposition towards microbiota-mediated diseases like IBDs, metabolic diseases or infections by enteric pathogens. We speculate that *MHC* gene evolution has in part been influenced by co-evolutionary forces between hosts and their resident commensal microbes.

While our study provides support for a model whereby MHC polymorphisms differentially influence antibody-mediated selection on the microbiota, there are a myriad of additional potential mechanisms by which MHC-mediated antigen presentation could influence commensal populations. MHC class II molecules present exogenously derived peptides to CD4^+^ helper T cells that coordinate immune responses against extracellular antigens. Recent studies have shown that multiple CD4^+^ T_H_ cell subsets (T_FH_, T_reg_ and T_H_17) directly recognize commensal antigens in the gut^44^,^45^. Each of these subsets are known to influence humoral immune responses against the gut microbiota, making both MHC-directed regulatory and inflammatory pathways capable of influencing microbial composition^15^,^24^,^29^,^46^. Recent work has also uncovered a previously unappreciated role for MHC class II presentation in innate lymphoid cells. Group 3 innate lymphoid cells utilize MHC class II presentation to anergize commensal-specific T cells to promote tolerance towards the microbiota^47^. Finally, gut enterocytes secrete a variety of antimicrobial molecules capable of influencing microbiota composition, and these cells express MHC class II on their surface^48^. Whether MHC class II expression by gut epithelial cells influences microbial communities is completely unknown, but warrants attention given the direct physical interaction between these cells and commensal microbes. Together, these studies highlight a central and functionally diverse role for MHC-mediated antigen presentation in modulating immune responses against commensal organisms. Future studies using conditional knockout mice will be crucial for empirically defining the process by which MHC antigen presentation sculpts microbiota architecture.

Results from our experiments suggest that MHC polymorphisms may contribute to the high degree of individuality observed in microbiota composition among humans. Understanding how MHC shapes microbiota diversity to influence colonization resistance could yield important insights into the treatment of emerging enteric infectious diseases of man. *Clostridium difficile* and *Campylobacter jejuni* are enteric bacterial pathogens that cause severe disease in susceptible people, and colonization resistance is known to play an important role in mitigating disease caused by these organisms^27^,^28^. In addition, microbiota transplants from uninfected donors have proven extremely effective at treating recurring *C. difficile*-induced colitis^49^. Therefore, identifying how host genotype and microbiota composition synergize to determine the phenomenon of colonization resistance has clear therapeutic potential. For example, HLA-microbiota typing, coupled with microbiota transplant screens in germfree animals, might be a useful strategy for identifying the most efficacious microbiotas (that is, donor source) for transplantation therapies. In addition, such a strategy might also be a useful methodology for identifying the core ‘colonization-resistance-promoting’ members of the microbiota that could be tested for their potential in future probiotic formulations. Similarly, identifying HLA alleles or haplotypes that predispose individuals to infection by these and other enteric pathogens might be a useful biomarker in preventive medicine by identifying at-risk members of the population that might benefit from a probiotic regiment of colonization-resistance-promoting species. These potential therapeutic benefits warrant further research as they might enhance personalized medicine.

## Methods

### Animal models

Eight-week-old male and female homozygous BALB/c MHC congenic mice (hereafter denoted H2^*bb*^ (C.B10-H-2^*b*^/LilMcdJ, cat#001952), H2^*dd*^ (BALB/cJ, cat#000651) and H2^*kk*^ (C.C3-H-2^*k*^/LilMcdJ, cat#001951)) were purchased from Jackson Laboratories and housed under SPF conditions at the University of Utah. To control for legacy effects across mouse strains, homozygote breeders were re-derived using the progeny of heterozygote crosses. Homozygote genotypes were confirmed by PAGE using linked microsatellite markers that discriminate among MHC genotypes used in these experiments. Germfree BALB/c mice were bred and reared in our own germfree facility. Age-matched male and female WT C57BL/6, MHCII^−/−^ and B2M^−/−^ were kindly provided by Dr Xianjian Chen. All animal use was in compliance with federal regulations and guidelines set forth by the University of Utah’s Institutional Animal Care and Use Committee (Protocol#14-05009).

### Pathogen model and experimental infections

The LT2 strain of *Salmonella enterica* (serovar *typhimurium*) was used in all experimental infections. *S. e. typhimurium* was grown in LB broth overnight and animals were subsequently administered *ca.* 10^4^ CFUs via oral gavage. The wasting disease caused by systemic *S. e. typhimurium* infection was quantified as weight lost during the course of infection. Differences in weight loss were calculated by comparing the percentages of body weight by day among treatment groups, as well as calculating the average weight lost per day from the beginning of weight loss (day 2) to the end of the experiment (day 7). To do this, the slope of the change in body weight by day was used to estimate the average loss of weight per day. Spleen weight and *S. e. typhimurium* loads in this organ are highly correlated and were both used to estimate systemic invasion. *S. e. typhimurium* CFUs were enumerated by plating spleen homogenates on MacKonkey Agar plates. No colonies are detected in the faeces or spleens of uninfected animals by this method.

### Immune phenotyping

Flow cytometry was performed on an LSR Fortessa instrument (BD Biosciences). Positive cell populations were defined by the use of single stain and/or appropriate isotype controls in all experiments. Representative flow cytometry plots defining cell populations that differed significantly among genotypes are provided in relevant figures. Cells were harvested from tissues as previously described^15^. For surface antibody staining, isolated cells were plated at 500,000 cells per well in a 96-well plate and washed twice using sterile HBSS buffer (CellGro) supplemented with 2.5% FBS (HyClone). Cells were then suspended in 100 μl of buffer containing fluorescently conjugated antibodies (1/250 (v/v) dilution) and placed in the dark at 4 °C for 20 min. Cells were washed twice with buffer to remove unbound antibody, and then analysed. For intracellular staining, cells were surface stained as described, and then fixed overnight. The next day, cells were washed to remove fixative and then stained in 50 μl of 1 × permeabilization buffer (eBiosciences) with fluorescently conjugated antibodies (1/50 (v/v) dilution) in the dark at 4 °C for 30 min. Cells were washed twice with buffer and then analysed. For summary of markers used to distinguish cell subsets and a full list of antibodies, working concentrations, and catalogue numbers used in all flow cytometry experiments refer to Supplementary Table 2.

### Microbiota transplantation into germfree mice

Microbiota stocks were derived from each MHC congenic genotype by scraping luminal contents from the gut (duodenum to rectum) of donor animals (*n*=2–3). Contents were diluted in sterile 1 × HBSS to create a 50:50 (v/v) mix. Mixtures were homogenized, vortexed for 20 s, and then spun at 400 × *g* for 10 min to separate course materials from the microbial suspension. Suspensions were then divided into 500 μl single-use aliquots, flash frozen in liquid nitrogen, and immediately stored at −80 °C until use. For colonization, age-matched germfree BALB/c mice were orally gavaged with 100 μl of a thawed aliquot of appropriate microbiota stock for 7 days. Colonization dynamics typical of this method are shown in Supplementary Fig. 5b.

### Microbiota sequencing

For analysis of faecal microbial communities, fresh faecal pellets were collected from 8- to 12-week-old animals and then stored at −80 °C until further use. For analysis of mucosally associated communities, the entire gut (duodenum to anus) was collected from an animal. Luminal contents were gently removed with forceps, and then tissues were rinsed in sterile 1 × HBSS. The luminal face of the gut epithelium was scraped using sterile forceps and immediately stored at −80 °C until further use. Three independent sequencing experiments were performed utilizing two different sequencing technologies; Illumina MiSeq and pyrosequencing. Bacterial genomic DNA was extracted using MO BIO PowerSoil DNA Isolation Kit (MO BIO Laboratories) for all experiments. Cage effects (for example, maternal effects, strain isolation effects, stochastic drift and so on) are unavoidable confounding variables in mouse microbiota studies that cannot be fully controlled for. As detailed below for each experiment, we have endeavoured to minimize its contribution to our overall findings by (1) sampling across multiple cages when possible, (2) deriving animals from heterozygous crosses in an attempt to equalize microbiota exposure across genotypes and (3) by performing three independent tests of the hypothesis that MHC antigen presentation sculpts microbiota composition using animals from different mouse facilities, representing different sexes and different genetic models, and by characterizing the effect of MHC genotype between two sites in the gut. Pyrosequencing experiment #1 (reported in Fig. 2a–d): Male animals from a long-term breeding colony of C57BL/6, B2M^−/−^, and MHCII^−/−^ animals maintained in the Pathology Department at the University of Utah were used to compare faecal communities in Fig. 2a–d. Animals were sampled across multiple cages per genotype. Sequencing runs for these samples were conducted at Baylor College of Medicine in the lab of our collaborator Dr Joseph Petrosino using a previously described pyrosequencing approach targeting the V3–V5 region of the bacterial 16S *rRNA* gene. The 16S *rRNA* gene libraries were generated by the Center for Metagenomics and Microbiome Research at Baylor College of Medicine, using the V3–V5 (357F/926R) primer in accordance with standard Human Microbiome Project protocols 50. The 16S rRNA libraries were sequenced by the Human Genome Sequencing Center at Baylor College of Medicine using a Roche 454 GS FLX+ instrument (Roche, Indianapolis, IN) operated with titanium chemistry. 16S *rRNA* gene sequences were assigned into OTUs at a similarity cutoff value of 97% using the UPARSE pipeline in QIIME^51^ and the SILVA Database^52^. Before analysis all samples were first rarified to a depth of 3,382 sequences per sample. Sequences corresponding to data shown in Fig. 2a–d, Supplementary Fig. 4a, Supplementary Fig. 4b and Supplementary Fig. 4e have been deposited at the NCBI SRA under accession number PRJNA296810. Illumina MiSeq experiment #1 (reported in Fig. 2e–h): Faecal pellets were collected from female MHC homozygote and heterozygote animals that were members of a long-term breeding colony maintained in the Pathology Department at the University of Utah. These animals served as the source animals for all other experiments described in this manuscript. The experiments described in Figs 2e–h and 4a represents the results of a single experiment where five animals from each of the three different MHC genotypes had their microbiotas sequenced. In the case of the H2^*dd*^ (green dots) and H2^*kk*^ (red dots) animals, the five animals from each genotype were both litter- and cagemates (that is, for each genotype, five female animals from the same litter were housed in the same cage and used for analysis). The H2^*bb*^ animals (blue dots) represent five female animals derived from three different mothers that were housed in three separate cages. This design provides an internal control for the possibility that drift or maternal effects significantly influences our results by demonstrating that H2^*bb*^ animals still significantly cluster by genotype when compared with the other two H2 congenic cohorts. Re-deriving homozygote congenic breeder pairs from heterozygote crosses, and using their progeny for microbiota sequencing analysis, minimizes any potentially confounding legacy effect due to the independent husbandry of these strains for several decades (the strain isolation effect). Also, five heterozygous H2^*bd*^ animals depicted in Fig. 4a were derived from a single breeder pair (H2^*dd*^ × H2^*bb*^ cross) that exposes animals to both H2^*bb*^ and H2^*dd*^ microbiota (Supplementary Fig. 7). From these animals, we obtained paired-end 300 nucleotide Illumina MiSeq reads from 16S *rRNA* gene variable regions 3 and 4, then processed and overlapped them using mothur 53 and QIIME^51^ as previously described^15^. Open reference 97% similarity OTU picking against the Greengenes 13_8 reference database was employed. Before analysis all samples were rarified to a depth of 16,000 sequences. Sequences corresponding to data shown in Figs 2e–h and 4a, Supplementary Fig. 2c and Supplementary Fig. 6a–d have been deposited at the NCBI SRA under accession number SRP062960. Pyrosequencing experiment #2 (reported in Fig. 2i–l): Faecal and mucosal samples were collected from separately housed male MHC congenic BALB/c animals from a long-term breeding colony housed in the Biology Department at the University of Utah to compare faecal and mucosal communities. Animals were sampled across multiple cages per genotype. Sequencing runs for these samples were conducted at Baylor College of Medicine in the lab of our collaborator Dr Joseph Petrosino. The 16S rDNA V4 region was amplified by PCR and sequenced in the MiSeq platform (Illumina) using a 2 × 250 nucleotide paired-end protocol yielding pair-end reads that overlap almost completely. The read pairs were demultiplexed based on the unique molecular barcodes, and reads were merged using USEARCH v7.0.1001 (ref. 54). 16S *rRNA* gene sequences were assigned into OTUs at a similarity cutoff value of 97% using the UPARSE pipeline in QIIME and the SILVA Database. Abundances were recovered by mapping the demultiplexed reads to the UPARSE OTUs. Before comparison of faecal and mucosal communities in male BALB/c animals, all samples were rarified to a depth of 560 sequences. For independent analysis of the effect of host genotype on faecal and mucosal communities, samples were rarified to a depth of 4,700 and 560 sequences, respectively. Sequences corresponding to data shown in Fig. 2i–l, Supplementary Fig. 4d and Supplementary Fig. 4e have been deposited at the NCBI SRA under accession number PRJNA296810.

### Ig repertoire sequencing

Peyer’s patches of age-matched MHC congenic animals were collected, placed in 500 μl of Qiazol RNA stabilization solution, and frozen at −80 °C until further processing. After phenol-chloroform extraction of RNA in the aqueous phase, we further cleaned the RNA using Qiagen RNeasy columns. Following DNase digestion and inactivation, the cleaned RNA from each sample was used as template for separate cDNA synthesis reactions for each Ig repertoire (IgA, IgD, IgG and IgM) using gene-specific primers (Supplementary Table 3) designed to target the CH1 region of the heavy chain constant region in BALB/c mice. Primers were designed based on alignments of IMGT reference sequences and degenerate nucleotides were introduced to account for multiple alleles when present or, in the case of IgG, to cover multiple subclasses. Each gene-specific cDNA was then split evenly and used as template among triplicate 25 μl PCR reactions using the same gene-specific primer used in cDNA synthesis as a reverse primer along with a mix of 17 forward primers (mixed based on abundance of their targets) previously identified^55^ as targeting BALB/c heavy chain variable (V_H_) regions. We appended partial Illumina adaptors to the 5′-end of these V_H_ primers (Supplementary Table 3) to serve as targets for primers in a second PCR. PCR using high-fidelity Phusion HotStart II polymerase was performed as follows: 98 °C initial denaturation for 2 min; 26 cycles of 98 °C for 20 s, 60 °C anneal for 20 s, 72 °C extension for 20 s; final single extension at 72 °C for 2 min. Triplicate PCR reactions were then combined and cleaned using Qiagen MinElute columns and eluted with 27 μl. 10 μl of the eluate was used as template for a second 50 μl PCR designed to add the rest of the Illumina adaptors to amplified products with the same reaction conditions as for the first PCR, except with only 16 cycles. Reverse primers targeting the constant heavy region for the second PCR were internal to the set used in the cDNA synthesis and first PCR, and had full Illumina adaptors with eight-nucleotide indexes appended to their 5′-end. Forward primers in the second PCR target the partial Illumina adaptors added to the V_H_ region during the first PCR and add the rest of the Illumina adaptors, as well as another eight-nucleotide index sequence (Supplementary Table 3). Final indexed reactions were subsequently cleaned with Qiagen MinElute columns, mixed evenly and submitted for sequencing on the Illumina MiSeq with paired-end 300 nucleotide reads. Sequences were initially processed to create a single, primer-trimmed, high-quality contig from overlapping paired-end sequences using the mothur command make.contigs and subsequently screened for sequences that did not have at least 20 nucleotides overlapping. To reduce the number of sequences analysed, we then used the OTU picking method within QIIME to cluster identical sequences and create biom table of counts of Ig sequences by samples for each repertoire. A representative sequence for each cluster of identical sequences was then submitted for analysis by IMGT/HighV-QUEST^56^. The resulting tables of Ig sequence characteristics (for example, V-region identity, CDR3 sequences and nucleotide substitution rates reported in Fig. 1) were then parsed and added to biom tables as observation metadata to facilitate downstream analyses. For all analyses each repertoire was rarefied to an even depth of sequences per sample.

### Ig-bound bacteria flow cytometry assay

Percentages of Ig-bound bacteria was measured in the faeces of animals. Briefly, fresh faecal pellets were collected and homogenized in 500 μl of sterile 1 × HBSS buffer. Faecal suspensions were spun and 400 × *g* for 5 min to precipitate course materials, and supernatants containing suspended bacteria were placed in new 1.5 ml Eppendorf tubes. Tubes were spun for 10 min at 8,000 × *g* to pellet bacteria and supernatants containing unbound antibodies were discarded. To eliminate unbound antibodies further, bacterial pellets were washed by re-suspending them in 1 ml of sterile 1 × HBSS, spinning at 8,000 × *g* for 5 min, and then discarding supernatants. Two washes were performed. An antibody against mouse IgA (Southern Biotech, PE-conjugated rat anti-mouse IgA, cat#1165-09L) was used to stain for IgA-bound bacteria. Antibody was diluted 1/500 in sterile 1 × HBSS containing 10% FBS as a blocking agent. Antibody stains were pipetted at 100 μl volumes into a round-bottomed 96-well plate. 5 μl of faecal bacteria suspensions were added to appropriate wells, mixed by pipetting, and then incubated in the dark at 4 °C for 30 min. Antibody stains were then removed by spinning plates at 4,000 r.p.m. and flicking off stain. Bacteria were washed twice by suspending in 200 μl of sterile 1 × HBSS, spinning at 4,000 r.p.m., and flicking off supernatants. Bacteria were then suspended in 250 μl of sterile 1 × HBSS containing 5 μl of 1 × SYBR green I stain (Molecular Probes). Bacteria were incubated in the dark at 4 °C for 20 min before enumeration on a flow cytometer. RAG1^−/−^ faecal bacteria samples were included in all experiments as negative controls.

### Faecal Ig ELISA

The concentration of IgA in faecal pellets were quantified using an IgA-specific ELISA kit (eBioscience: Mouse IgA Ready-SET-go kit (cat#88-90450-88)) following kit protocols. All concentration estimates are standardized by faecal weight and depicted as concentration per gram of faeces.

### Quantitative PCR

All quantitative PCR reactions were conducted in 12.5 μl volumes using the SYBR green Master Mix (Roche). Quantitative PCR experiments were conducted on a Lightcycler LC480 instrument (Roche). Template quantity and quality was assessed using a Nanodrop spectrophotometer. Abundance estimates are standardized to the concentration of input DNA per reaction and are represented as copies per nanogram of faecal DNA. Template extraction for quantification of faecal bacteria loads: DNA was extracted from fresh faecal pellets using the PowerFecal DNA Isolation Kit (Mo Bio) following kit instructions. Bacterial loads were quantified using previously validated bacterial group-specific 16S primers (Supplementary Table 3).

### Statistics

Statistics were carried out using JMP9.0 (SAS), Prism 6.0 (Graphpad) and R software. permutational analysis of variance was used for hypothesis testing of significance between groups shown in PcoA plots. A multivariate analysis of variance was used in Fig. 3g to test for a significant difference in the effect of colonization with different microbiotas on the abundance of immune cell parameters (a sum model was employed).

A two-tailed unpaired Student’s *t*-test was used for all other pairwise statistical comparisons unless otherwise noted. A Welch’s correction was applied when a Levine’s test revealed unequal variance in otherwise normally distributed data sets. A nonparametric Mann–Whitney *U*-test was used on highly skewed data sets (that is, *Salmonella* CFU data). Error bars in all figures represent ±s.d. with the exception of Figures depicting trends in weight loss where error bars represent ±s.e.m. Before data analysis outliers were identified and excluded using the ROUT method in PRISM 6.0. A minimum sample size of five mice were used for all experiments. Sample size, #replicates, statistical test and estimates of dispersion are reported in all figure captions.

## Additional information

**How to cite this article:** Kubinak, J. L. *et al.* MHC variation sculpts individualized microbial communities that control susceptibility to enteric infection. *Nat. Commun.* 6:8642 doi: 10.1038/ncomms9642 (2015).

## Supplementary Material

Supplementary Information Supplementary Figures 1-7 and Supplementary Tables 1-6
