# Supplementary material for: MHC variation sculpts individualized microbial communities that control susceptibility to enteric infection
Source: Nat Commun. 2015 Oct 23;6:8642. doi: 10.1038/ncomms9642 (PMC4621775; doi:10.1038/ncomms9642)
Supplement: Supplementary Information — Supplementary Figures 1-7 and Supplementary Tables 1-6 [file ncomms9642-s1.pdf]

Supplementary Figures

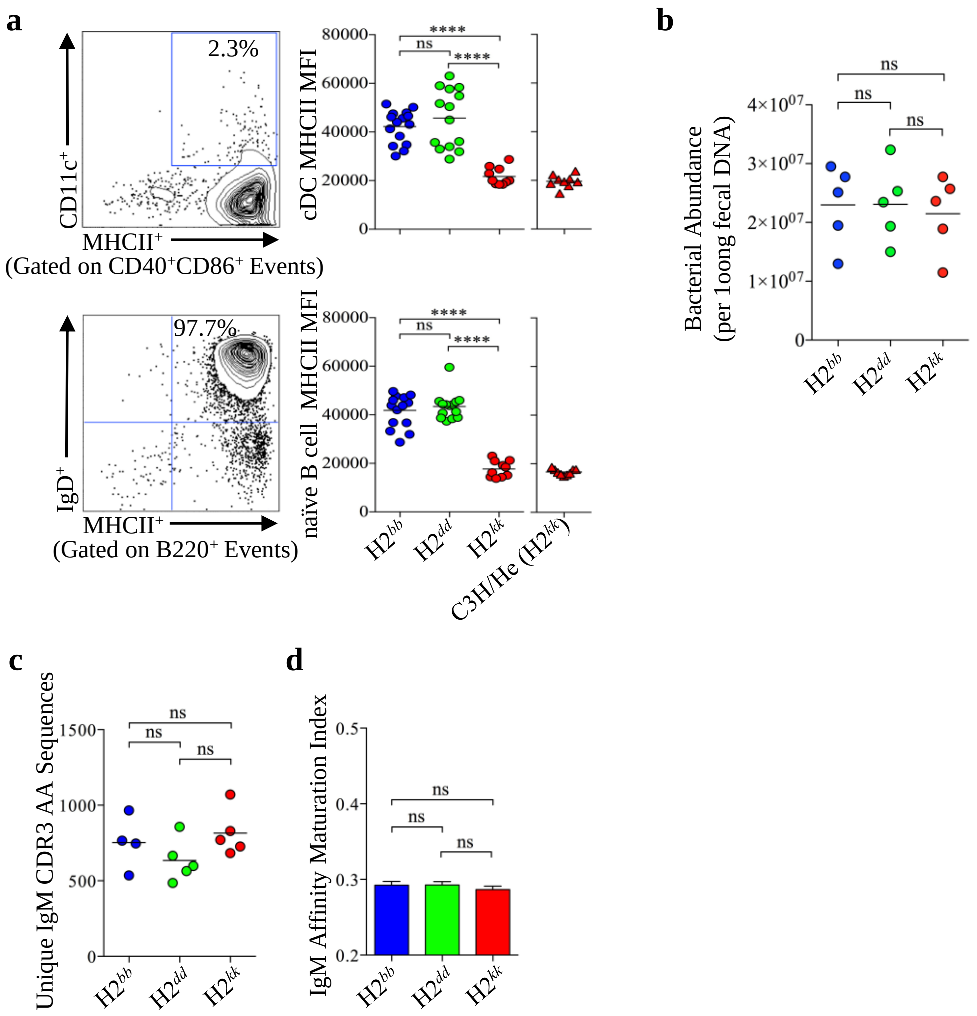

Supplementary Figure 1 | MHC mediates IgA response against gut microbiota. (a) Representative F.C. plots and data sets comparing the relative surface expression of MHC on dendritic cells and naïve B cells from MHC congenic animals ($H2^{bb}$-n=15; $H2^{dd}$-n=14; $H2^{kk}$-n=15, C3H/HeJ-n=9). **(b)** There is no difference in the relative abundance of fecal bacteria in H2 congenic animals ($H2^{bb}$-n=5, $H2^{dd}$-n=5, $H2^{kk}$-n=5). **(c,d)** Comparison of naïve IgM repertoire among MHC genotypes ($H2^{bb}$-n=4, $H2^{dd}$-n=5, $H2^{kk}$-n=5). There is no difference between MHC genotypes when (c) sequence diversity and (d) affinity maturation scores of the naïve IgM repertoire are compared among MHC genotypes. (a-c) Bars represent group means. (d) Error bars represent S.E.M. A two-tailed unpaired Student's t-test was used for all pair-wise statistical comparisons (****=p<0.00001; ***=p<0.001; **=p<0.01; *=p<0.05).

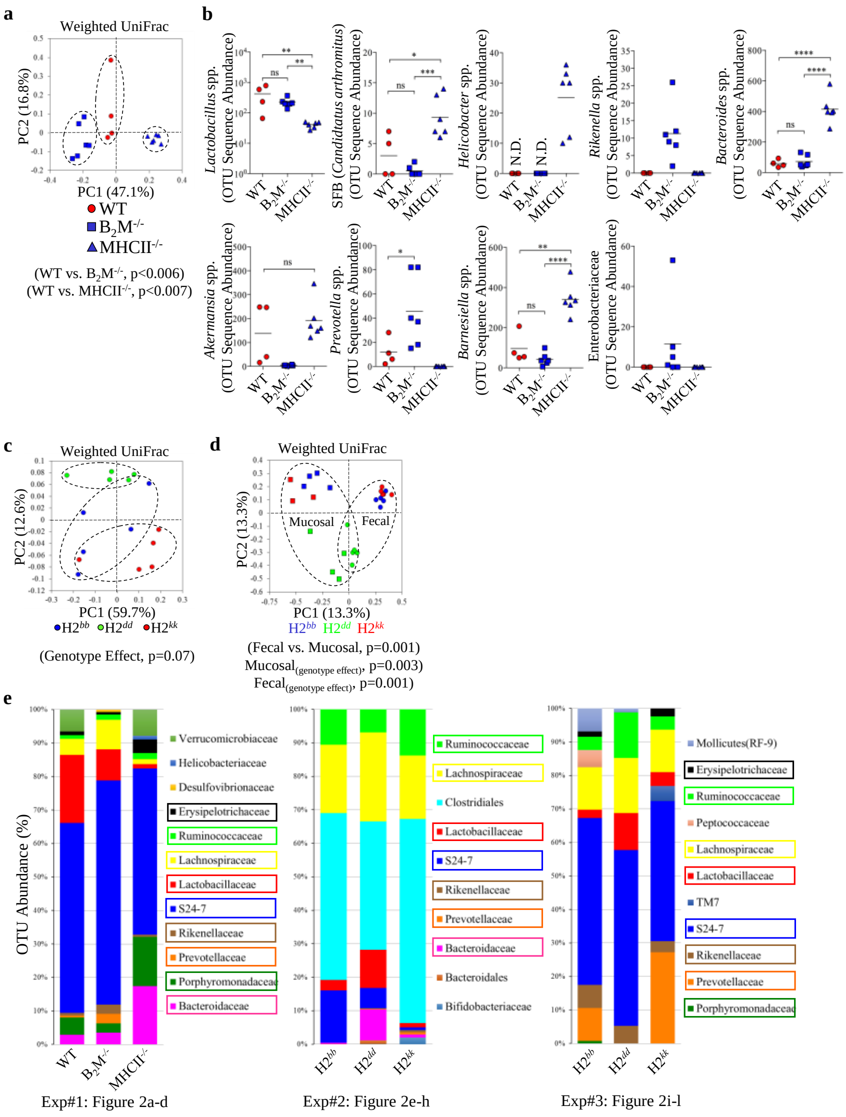

Supplementary Figure 2 | MHC sculpts microbiota composition in the gut. (a) PcoA plot based on weighted UniFrac analysis of fecal microbiotas from WT, $B_2M^{-/-}$, and $MHCII^{-/-}$ animals. **(b)** OTU abundance plots of specific bacterial groups among WT, $B_2M^{-/-}$, and $MHCII^{-/-}$ animals. "N.D"=not detected. All pairwise comparisons are results of Student's t-test (****=p<0.0001; ***=p<0.001; **=p<0.01; *=p<0.05). **(c)** PcoA plot based on weighted UniFrac analysis of fecal microbiotas from female H2 congenic animals. **(d)** PcoA plot based on weighted UniFrac analysis of fecal microbiotas from male H2 congenic animals used for comparison of fecal and microbiota communities. (a,c,d) P-values represent the results of a PERMANOVA based on 999 simulations. Ellipses in PcoA plots are for illustrative purposes only and are non-quantitative. **(e)** Stacked bar charts represent relative abundance among significantly differentially detected OTUs within each bacterial clade. Bacterial families consistently enriched for significant MHC effects across the independent experiments described in Figure 2 are highlighted by colored boxes (see Supplementary Tables 4-6 for full list of OTUs and results of kruskal-wallis statistical tests).

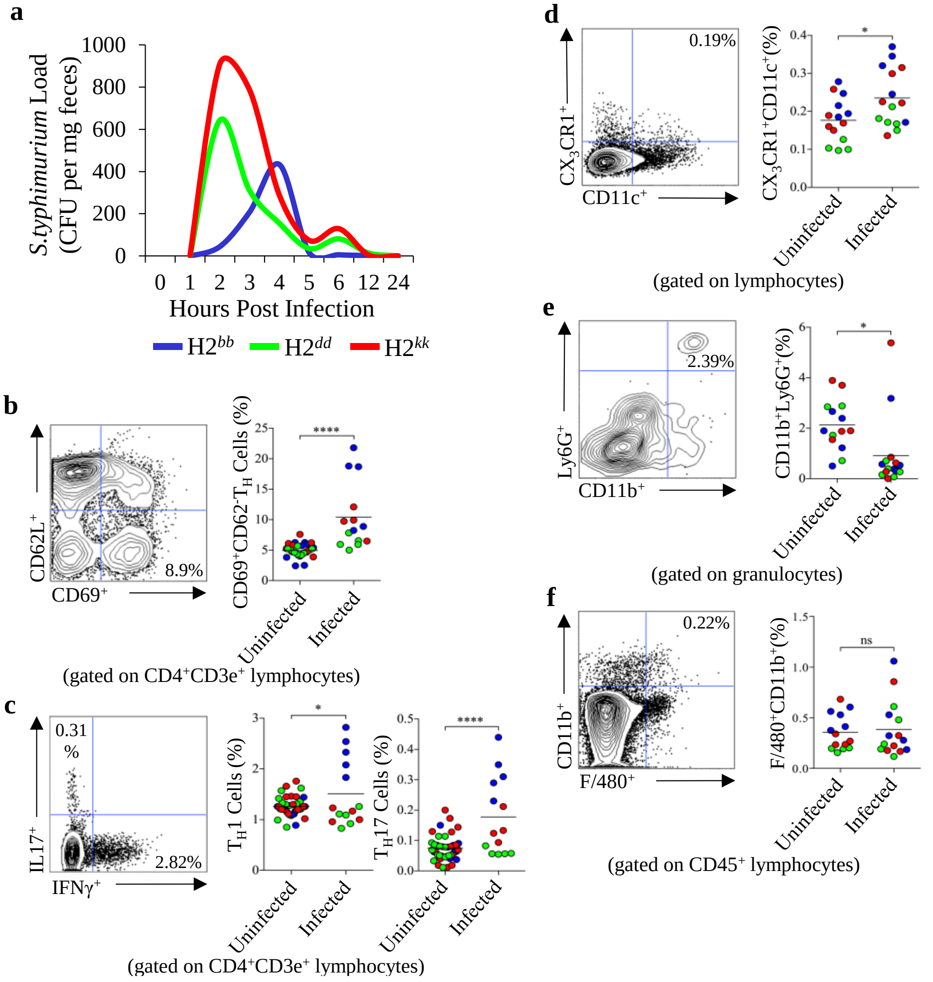

Supplementary Figure 3 | MHC mediates susceptibility to *S.e.typhimurium* infection and this is not due to an enhanced immune response in resistant genotypes. (a) Qualitative analysis of Salmonella loads in the feces of mice (n=3-5 per genotype) at defined time-points after oral gavage of 10^4 CFUs. Fecal pellets were collected at each time point, homogenized with pestel, and plated on MacConkey agar. Salmonella loads are standardized per mg feces. A smoothed trendline is shown. **(b,c)** Results of F.C. experiments demonstrating that infection of $H2^{bb}$ (n=5), but not $H2^{dd}$ (n=5) or $H2^{kk}$ (n=5), elicits an immune response as measured by increases in the abundance of (b) activated $CD4^+$ T_H cells, and (c) inflammatory T_H1 and T_H17 cells. **(d-f)** Results of F.C. experiments demonstrating that infection does not result from differential recruitment of phagocytes ($CX_3CR1^+CD11c^+$ monocytes, $Ly6G^+CD11b^+$ neutrophils, $CD11b^+F/480^+$ macrophages) among H2 congenic genotypes. (b-f) Representative F.C. plots are provided for all F.C. data sets (percentages are provided to identify relevant subgate). Unpaired two-tailed Student's t-test (****=p<0.0001; ***=p<0.001; **=p<0.01; *p<0.05).

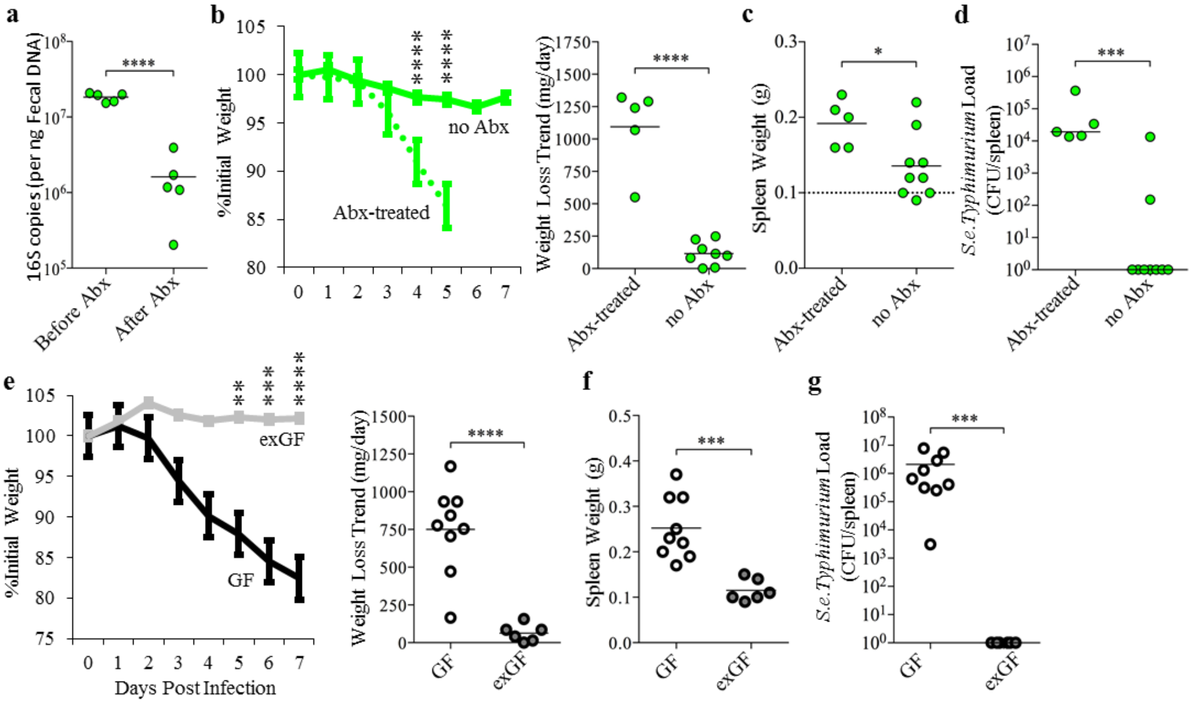

Supplementary Figure 4 | Susceptbility to *S.e.typhimurium* infection is microbiota-dependent. (a-d) Disease scores from Salmonella-infected (10^4 CFU via oral gavage) resistant $H2^{dd}$ animals, and $H2^{dd}$ animals that had been administered antibiotics in their drinking water for 3 days (0.5mg/mL of each of the following: Neomycin Sulfate, Ampicillin, Erythromycin, Gentamycin). Animals were taken off antibiotics 24 hours prior to infecting with 10^4 *S.e.typhimurium* CFUs via oral gavage. **(e-g)** Disease scores of Salmonella-infected (10^4 CFU via oral gavage) GF animals and animals that were born GF and were subsequently reared from birth in and SPF facility until 9 weeks of age (i.e. exGF). (b-right panel, c, e-right panel, f) Results of unpaired two-tailed Student's t-test (****=p<0.0001; ***=p<0.001; **=p<0.01; *=p<0.05). (b and e (left panels)) Error bars represent S.E.M. Asterisks represent significant differences based on results of t-tests comparing weight loss by day and reflect significant differences between Abx-treated and untreated $H2^{dd}$ animals in (b) and GF and exGF animals in (e). (d and g) (c,f) Asterisks represent significance based on results of Mann-Whitney U Test, (***=p<0.001).

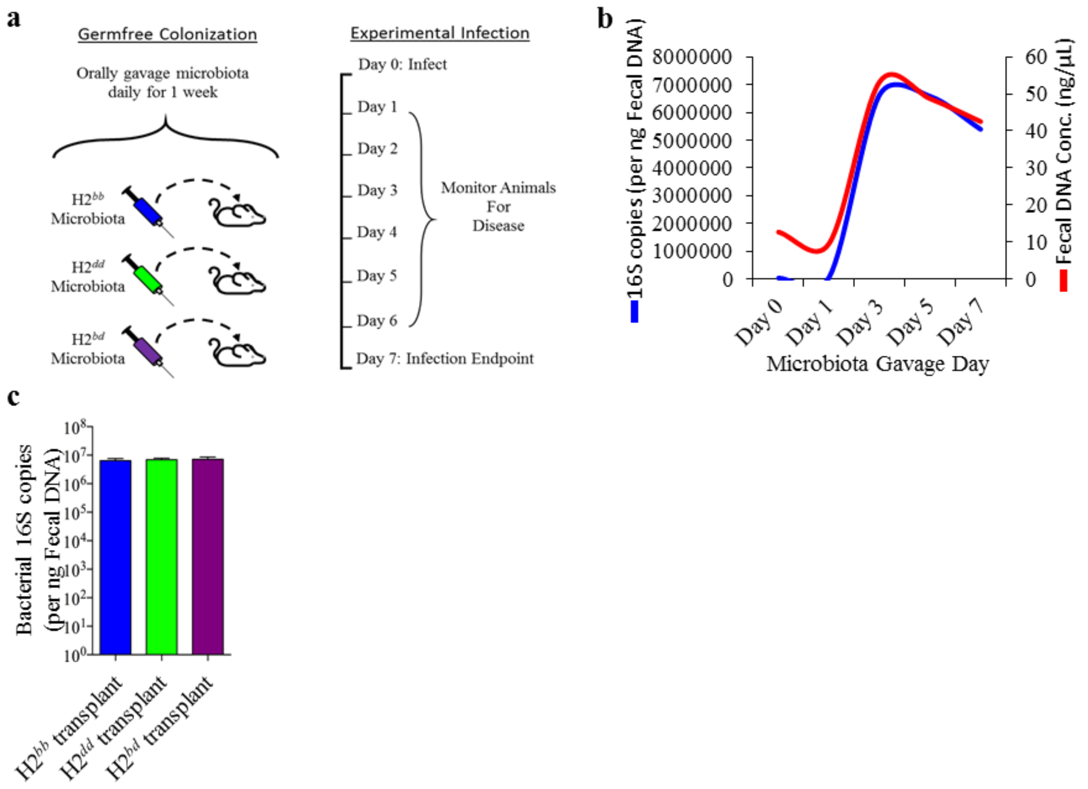

Supplementary Figure 5 | Fecal transplants recapitulate patterns of susceptibility in colonized GF animals. (a) Illustration for design of microbiota colonization, and Salmonella infection experiments. **(b)** Dynamics of microbiota colonization of GF animals as inferred from fecal bacterial loads and increasing fecal DNA concentrations over the course of 7 day colonizations (T_0-n=4; T_1-n=2; T_3-n=2; T_5-n=1; T_7-n=19). **(c)** Results of Q-PCR experiment quantifying fecal bacterial loads in GF animals that had been colonized with microbiota derived from $H2^{bb}$ (n=8), $H2^{dd}$ (n=4), and $H2^{bd}$ (n=7)(Day 7 time-point).

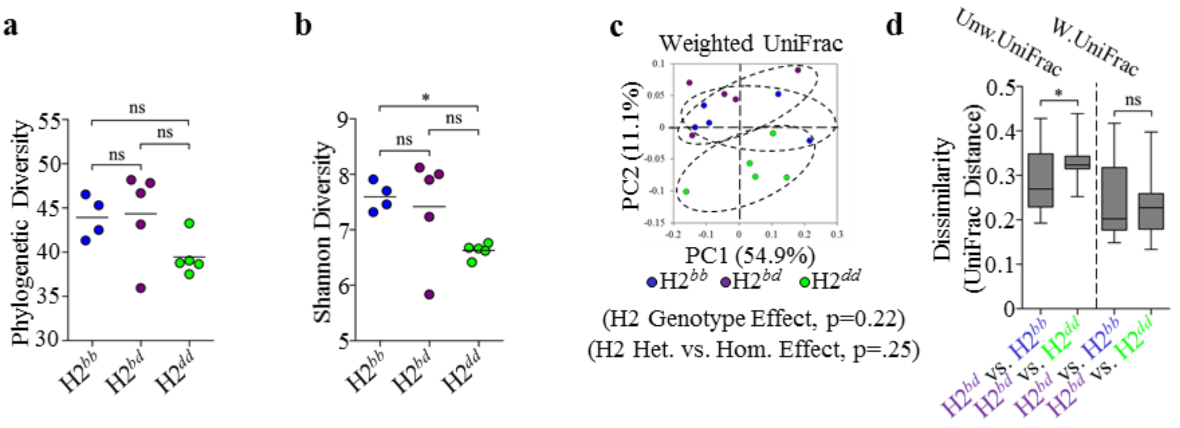

Supplementary Figure 6 | MHC heterozygosity influences microbiota composition. (a) PcoA plot based on weighted UniFrac analysis of fecal microbiotas from H2^{bb}, H2^{dd}, and H2^{bd} animals. The reported p-values represent the results of a PERMANOVA based on 999 simulations testing the effect of host genotype, or homozygosity versus heterozygosity. **(b,c)** Within-community estimates of (b) phylogenetic diversity and (c) Shannon diversity. **(d)** Distance boxplots of community similarity between H2^{bd} heterozygotes and each respective H2^{bb} and H2^{dd} homozygote parent strains based on unweighted and weighted UniFrac analysis. (b-d) Unpaired two-tailed Student's t-test ($p<0.05$=*).

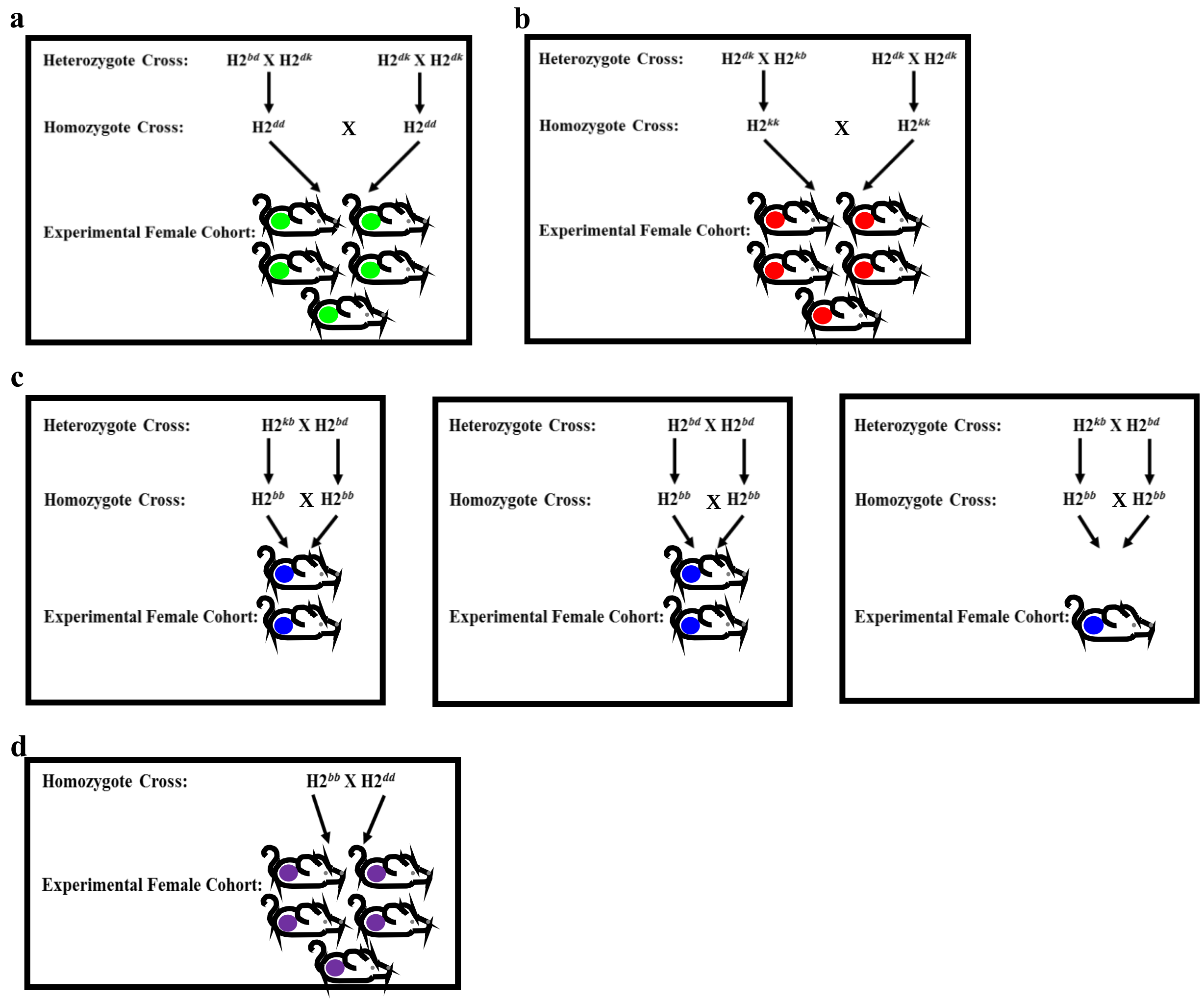

Supplementary Figure 7 | Breeding design for female MHC congenic mice used for microbiota sequencing experiment described in Figure 2e-h. The experiments described in Figure 2e-h and 4a represents the results of a single experiment where 5 animals from each of the three different MHC genotypes had their microbiotas sequenced. **(a)** In the case of the H2^{dd} (green dotted mice) and **(b)** H2^{kk} (red dotted mice) animals, the 5 animals from each genotype were both litter- and cage-mates (i.e. for each genotype, 5 female animals from the same litter were housed in the same cage and used for analysis). **(c)** The H2^{bb} animals (blue dotted mice) represent five female animals derived from three different mothers that were housed in three separate cages. This design provides an internal control for the possibility that drift or maternal effects significantly influences our results by demonstrating that H2^{bb} animals still significantly cluster by genotype when compared to the other two H2 congenic cohorts. (a-c) Re-deriving homozygote congenic breeder pairs from heterozygote crosses, and using their progeny for microbiota sequencing analysis, excludes the confounding effect of the independent husbandry of these strains for several decades (the strain isolation effect). **(d)** 5 heterozygous H2^{bd} animals (purple dotted mice) depicted in Figure 4a were derived from a single breeder pair (H2^{dd} x H2^{bb} cross) which exposes animals to both H2^{bb} and H2^{dd} microbiota.

Supplementary Table 1. MHC Haplotypes of H2 congenic BALB/c mice

	**Class Ia**	**Class IIa**				**Class Ia**	
**Locus**	**K**	**Aβ**	**Aα**	**Eβ**	**Eα**	**D**	**L**
**H2^{d} haplotype**	d	d	d	d	d	d	d
**H2^{b} haplotype**	b	b	b	b	^	b	b
**H2^{k} haplotype**	k	k	k	k	k	k	^

^: null allele
Adapted from [1]

Supplementary Table 2. Cell Subset, Markers, and Antibodies used for flow cytometry

Cell Subset	Markers
$CD4^+$ T_H Cells	$CD4^+CD3\varepsilon^+$
$CD8^+$ T_{CT} Cells	$CD8\alpha^+CD3\varepsilon^+$
Activated $CD4^+$ T_H Cells	$CD4^+CD3\varepsilon^+CD62L^-CD69^+$
Activated $CD8^+$ T_{CT} Cells	$CD8\alpha^+CD3\varepsilon^+CD62L^-CD69^+$
Tolerogenic Dendritic Cells	$CD11c^+CD103^+$
T_{reg} Cells	$CD4^+CD3\varepsilon^+FoxP3^+$
IL10+ Treg Cells	$CD4^+CD3\varepsilon^+FoxP3^+IL10^+$
T_H1 Cells	$CD4^+CD3\varepsilon^+IFN\gamma^+$
T_H17 Cells	$CD4^+CD3\varepsilon^+IL17^+$
T_{FH} Cells	$CD4^+CD3\varepsilon^+CXCR5^+PD1^+$
Naïve B Cells	$B220^+IgD^{hi}MHCII^+$
GC B Cells	$B220^+IgD^{lo}GL7^+FAS^+$
IgA^+ B Cells	$B220^+CD138^-IgA^+$
IgA^+ Plasma Cells	$B220^-CD138^+IgA^+$
Conventional Dendritic Cells	$CD40^+CD86^+CD11c^+MHCII^+$

CD4-FITC (1/250 dilution, clone RM4-5,eBioscience cat#11-0042) CD8α-PerCP.Cy5.5 (1/250 dilution, clone 53-6.7,BioLegend cat#100733); CD3ε-Pacific Blue (1/250 dilution, clone 145-2C11,eBioscience cat#48-0031); CD62L-PE (1/250 dilution, clone MEL-14,BioLegend cat#104407); CD69-PE/Cy7 (1/250 dilution, clone H1.2F3,BioLegend cat#104511); CD11c-APC (1/250 dilution, clone N418,eBioscience cat#17-0114); CD103-PE (1/250 dilution, clone 2E7,BioLegend cat#121405); FoxP3-APC (1/50 dilution, clone FJK-16s,eBioscience cat#17-5773); FoxP3-PerCP.Cy5.5 (1/50 dilution, clone FJK-16s,eBioscience cat#45-5773); IL10-PE (1/50 dilution, clone JES5-16E3,eBioscience cat#12-7101); IFNγ-PE (1/50 dilution, clone XMG1.2,BioLegend cat#505807); IL17A-eFluor660 (1/250 dilution, clone eBio17B7,eBioscience cat#50-7177); CXCR5-PE (1/250 dilution, clone SPRCL5,eBioscience cat#12-7185); CXCR5 isotype control Rat IgG2a-PE (1/250 dilution, clone eBR2a,eBioscience cat#9012-4321); PD1-PE/Cy7 (1/250 dilution, clone RMP1-30,BioLegend cat#109109); PD1 isotype control Rat IgG2b-PE/Cy7 (1/250 dilution, clone RTK4530,BioLegend cat#400617); B220-PerCP.Cy5.5 (1/500 dilution, clone RA3-6B2,BioLegend cat#103235); IgD-AlexaFluor647 (1/250 dilution, clone 11-26c.2a,BioLegend cat#405707); MHCII(IA/IE)-PE (1/250 dilution, clone M5/114.15.2,BioLegend cat#107607); MHCII-IA^k-PE (1/250 dilution, clone 10-3.6,BioLegend cat#109908); MHCII isotype control Rat IgG2b-PE (1/250 dilution, clone eB149,eBioscience cat#12-4031); GL7-AlexaFluor488 (1/250 dilution, clone GL-7,eBioscience cat#53-5902); FAS-PE/Cy7 (1/250 dilution, clone Jo2,BD Biosciences cat#557653); CD138-PE (1/250 dilution, clone 281-2,BioLegend cat#142503); Rat anti-mouse IgA-PE (1/500 dilution, SouthernBiotech catalog#1165-09L); CD40-FITC (1/250 dilution, clone HM40-3,BioLegend cat#102905); CD86-PerCP.Cy5.5 (1/250 dilution, clone GL-1,BioLegend cat#105027); Goat anti-mouse IgA-FITC (1/250 dilution, SouthernBiotech cat#1040-02)

Notes on antibody staining:
-IgA-PE was used to quantify IgA-bound fecal bacteria
-FoxP3-PerCP.Cy5.5 was used to stain Tregs in PPs

-The IA/IE antibody does not bind IA molecules from mice carrying the $H2^{kk}$ haplotype. To overcome this, an IA^{k}-specific antibody was mixed with the IA/IE antibody in all MHCII stains.

Supplementary Table 3. Primers used in study

Use	Target	Sequence (1,2)	Reference
Bacterial qPCR	Eubacteria	Forward: 5-ACTCCTACGGGAGGCAGCAGT-3<br>Reverse: 5-ATTACCGCGGCTGCTGGC-3	2
Bacterial qPCR	Bacteroides/Prevotella spp.	Forward: 5-CCTWCGATGGATAGGGGTT-3<br>Reverse: 5-CACGCTACTTGGCTGGTTCAG-3	3
Bacterial qPCR	Lactobacillus/Lactococcus spp.	Forward: 5-AGCAGTAGGGAATCTTCCA-3<br>Reverse: 5-CACCGCTACACATGGAG-3	4
Ig repertoire cDNA synthesis, first PCR	IgA C_H Region	AACTGGCTGCTCATGGTGTACC	
Ig repertoire cDNA synthesis, first PCR	IgD C_H Region	TATGGTGCAAGTGTGGTTGAGG	
Ig repertoire cDNA synthesis, first PCR	IgG C_H Region	CTGGACAGGGMTCCAKAGTTC	
Ig repertoire cDNA synthesis, first PCR	IgM C_H Region	CTCTTGGGAGACAGCAASACC	
Ig repertoire, first PCR	Ig V_H Regions	**ACACTCTTTCCCTACACGACGCTCTTCCGATCT**(N_{19-21})	5
Ig repertoire, second PCR	Ig first PCR product	**AATGATACGGCGACCACCGAGATCTACACXXXXXXXX ACACTCTTTCCCTACACGACGC**	
Ig repertoire, second PCR	Ig first PCR product – IgA C_H	**CAAGCAGAAGACGGCATACGAGATXXXXXXXXGTGAC TGGAGTTCAGACGTGTGCTCTTCCGATCT**GATGGTGGG ATTTCTCGCAGA	
Ig repertoire, second PCR	Ig first PCR product – IgD C_H	**CAAGCAGAAGACGGCATACGAGATXXXXXXXXGTG ACTGGAGTTCAGACGTGTGCTCTTCCGATCT**GGGCT TTGCACTCTGAGAGG	
Ig repertoire, second PCR	Ig first PCR product – IgG C_H	**CAAGCAGAAGACGGCATACGAGATXXXXXXX XGTGACTGGAGTTCAGACGTGTGCTCTTCCGA TCT**GGRCCARKGGATAGACHGATG	
Ig repertoire, second PCR	Ig first PCR product – IgM C_H	**CAAGCAGAAGACGGCATACGAGATXXXXXXXXG TGACTGGAGTTCAGACGTGTGCTCTTCCGATCT**G GGAAGACATTTGGGAAGGAC	

1. N_{19-21} represents the 17 "external" primers identified by Rohatgi et al., 2008 to target the 16 different V_H region families.

2. **Bold** sequences are Illumina adapter sequences. XXXXXXXX represents index sequences.

Supplementary Table References

1 Flurkey, K., Currer, J. M., Leiter, E. H. & Witham, B. (The Jackson Laboratory, Bar Harbor, ME, 2009).

2 Amann, R. I. *et al.* Combination of 16S rRNA-targeted oligonucleotide probes with flow cytometry for analyzing mixed microbial populations. *Applied and environmental microbiology* **56**, 1919-1925 (1990).

3 Furet, J. P. *et al.* Comparative assessment of human and farm animal faecal microbiota using real-time quantitative PCR. *FEMS microbiology ecology* **68**, 351-362, doi:10.1111/j.1574-6941.2009.00671.x (2009).

4 Rinttila, T., Kassinen, A., Malinen, E., Krogius, L. & Palva, A. Development of an extensive set of 16S rDNA-targeted primers for quantification of pathogenic and indigenous bacteria in faecal samples by real-time PCR. *Journal of applied microbiology* **97**, 1166-1177, doi:10.1111/j.1365-2672.2004.02409.x (2004).

5 Rohatgi, S., Ganju, P. & Sehgal, D. Systematic design and testing of nested (RT-)PCR primers for specific amplification of mouse rearranged/expressed immunoglobulin variable region genes from small number of B cells. *Journal of immunological methods* **339**, 205-219, doi:10.1016/j.jim.2008.09.017 (2008).

Supplementary Table 4. WT, B2M-/-, MHCII-/- Significant differences by OTU*

OTU	Test-Statistic	P	FDR_P	WT mean	B2M_KO mean	MHCII KO mean	taxonomy
FJ503633	14.1176471	0.00085979	0.0501624	0	0	2.83333333	Bacteria;__Bacteroidetes;__Bacteroidia;__Bacteroidales;__Bacteroidaceae;__Bacteroides
EU655886	13.2743363	0.00131073	0.05258272	0.5	8.33333333	170	Bacteria;__Bacteroidetes;__Bacteroidia;__Bacteroidales;__Bacteroidaceae;__Bacteroides
EF097042	13.121118	0.00141509	0.05258272	6.75	43.5	0	Bacteria;__Bacteroidetes;__Bacteroidia;__Bacteroidales;__Bacteroidaceae;__Bacteroides
FJ959620	11.8603604	0.002658	0.05258272	0	0.33333333	3	Bacteria;__Bacteroidetes;__Bacteroidia;__Bacteroidales;__Bacteroidaceae;__Bacteroides
GQ448246	11.8537415	0.00266681	0.05258272	0	0.33333333	3	Bacteria;__Bacteroidetes;__Bacteroidia;__Bacteroidales;__Bacteroidaceae;__Bacteroides
DQ807372	11.7855754	0.00275927	0.05258272	8.5	0	13.8333333	Bacteria;__Bacteroidetes;__Bacteroidia;__Bacteroidales;__Bacteroidaceae;__Bacteroides
DQ809036	11.1111111	0.00386592	0.05258272	0	0	1	Bacteria;__Bacteroidetes;__Bacteroidia;__Bacteroidales;__Bacteroidaceae;__Bacteroides
EU453726	11.0640648	0.00395794	0.05258272	34.75	17.3333333	204.833333	Bacteria;__Bacteroidetes;__Bacteroidia;__Bacteroidales;__Bacteroidaceae;__Bacteroides
EU767893	10.8932462	0.00431084	0.05258272	0	0	2	Bacteria;__Bacteroidetes;__Bacteroidia;__Bacteroidales;__Bacteroidaceae;__Bacteroides
EU766274	10.8841724	0.00433044	0.05258272	6.25	0	0.83333333	Bacteria;__Bacteroidetes;__Bacteroidia;__Bacteroidales;__Bacteroidaceae;__Bacteroides
JN084198	9.12280702	0.01044739	0.08500373	0	0.16666667	2	Bacteria;__Bacteroidetes;__Bacteroidia;__Bacteroidales;__Bacteroidaceae;__Bacteroides
EU457166	11.7820324	0.00276417	0.05258272	96.5	42.5	341.166667	Bacteria;__Bacteroidetes;__Bacteroidia;__Bacteroidales;__Porphyromonadaceae;__Barnesiella
EU655728	11.2871674	0.00354016	0.05258272	3.25	4.33333333	0	Bacteria;__Bacteroidetes;__Bacteroidia;__Bacteroidales;__Porphyromonadaceae;__Odoribacter
EU505366	14.0077821	0.00090834	0.0501624	0	7.16666667	0	Bacteria;__Bacteroidetes;__Bacteroidia;__Bacteroidales;__Porphyromonadaceae;__Parabacteroides
HQ791502	11.1111111	0.00386592	0.05258272	0	1	0	Bacteria;__Bacteroidetes;__Bacteroidia;__Bacteroidales;__Porphyromonadaceae;__Parabacteroides
AY993153	13.9805825	0.00092078	0.0501624	0	11.5	0	Bacteria;__Bacteroidetes;__Bacteroidia;__Bacteroidales;__Prevotellaceae;__g
EU505099	13.9805825	0.00092078	0.0501624	0	16	0	Bacteria;__Bacteroidetes;__Bacteroidia;__Bacteroidales;__Prevotellaceae;__Prevotella
EF097482	12.2719332	0.00216363	0.05258272	11.75	29.6666667	0	Bacteria;__Bacteroidetes;__Bacteroidia;__Bacteroidales;__Prevotellaceae;__Prevotella
EU451986	14.0077821	0.00090834	0.0501624	0	5.33333333	0	Bacteria;__Bacteroidetes;__Bacteroidia;__Bacteroidales;__Rikenellaceae;__Alistipes
DQ815937	11.5521327	0.00310089	0.05258272	0.25	0.5	4.83333333	Bacteria;__Bacteroidetes;__Bacteroidia;__Bacteroidales;__Rikenellaceae;__Alistipes
EU458021	10.8494065	0.00440637	0.05258272	14.75	0.83333333	10.5	Bacteria;__Bacteroidetes;__Bacteroidia;__Bacteroidales;__Rikenellaceae;__Alistipes
EU655722	14.0900196	0.00087175	0.0501624	0	36.1666667	0	Bacteria;__Bacteroidetes;__Bacteroidia;__Bacteroidales;__Rikenellaceae;__RC9_gut_group

EF097211	14.0077821	0.00090834	0.0501624	0	6.5	0	Bacteria;__Bacteroidetes;__Bacteroidia;__Bacteroidales;__Rikenellaceae;__Rikenella
EU655856	10.9170306	0.00425988	0.05258272	0	4.83333333	0	Bacteria;__Bacteroidetes;__Bacteroidia;__Bacteroidales;__Rikenellaceae;__Rikenella
EU504717	14.6564885	0.00065673	0.0501624	2.75	0	0	Bacteria;__Bacteroidetes;__Bacteroidia;__Bacteroidales;__S24-7;__g
EU655810	14.6564885	0.00065673	0.0501624	8.25	0	0	Bacteria;__Bacteroidetes;__Bacteroidia;__Bacteroidales;__S24-7;__g
EF097618	14.6192893	0.00066905	0.0501624	4.75	0	0	Bacteria;__Bacteroidetes;__Bacteroidia;__Bacteroidales;__S24-7;__g
EF099831	14.6192893	0.00066905	0.0501624	6.75	0	0	Bacteria;__Bacteroidetes;__Bacteroidia;__Bacteroidales;__S24-7;__g
EU760449	14.6192893	0.00066905	0.0501624	144.75	0	0	Bacteria;__Bacteroidetes;__Bacteroidia;__Bacteroidales;__S24-7;__g
EU504981	14.0350877	0.00089602	0.0501624	0	0	7.33333333	Bacteria;__Bacteroidetes;__Bacteroidia;__Bacteroidales;__S24-7;__g
EU453733	14.0077821	0.00090834	0.0501624	0	5.5	0	Bacteria;__Bacteroidetes;__Bacteroidia;__Bacteroidales;__S24-7;__g
EU504471	14.0077821	0.00090834	0.0501624	0	3	0	Bacteria;__Bacteroidetes;__Bacteroidia;__Bacteroidales;__S24-7;__g
EU656052	13.9805825	0.00092078	0.0501624	0	19.6666667	0	Bacteria;__Bacteroidetes;__Bacteroidia;__Bacteroidales;__S24-7;__g
EU504401	13.9805825	0.00092078	0.0501624	0	0	7.33333333	Bacteria;__Bacteroidetes;__Bacteroidia;__Bacteroidales;__S24-7;__g
EU505458	13.9805825	0.00092078	0.0501624	0	317.166667	0	Bacteria;__Bacteroidetes;__Bacteroidia;__Bacteroidales;__S24-7;__g
JQ085115	13.5338346	0.00115124	0.05258272	0	6.33333333	88.8333333	Bacteria;__Bacteroidetes;__Bacteroidia;__Bacteroidales;__S24-7;__g
EU510133	13.4328358	0.00121087	0.05258272	0	10.3333333	26.8333333	Bacteria;__Bacteroidetes;__Bacteroidia;__Bacteroidales;__S24-7;__g
EU510097	13.121118	0.00141509	0.05258272	10.75	4.83333333	0	Bacteria;__Bacteroidetes;__Bacteroidia;__Bacteroidales;__S24-7;__g
EU510667	13.0580357	0.00146044	0.05258272	0.25	16.6666667	0	Bacteria;__Bacteroidetes;__Bacteroidia;__Bacteroidales;__S24-7;__g
EU512010	12.7067669	0.00174085	0.05258272	19.5	7.83333333	1	Bacteria;__Bacteroidetes;__Bacteroidia;__Bacteroidales;__S24-7;__g
EF097520	12.6876877	0.00175753	0.05258272	3.25	0.66666667	15.1666667	Bacteria;__Bacteroidetes;__Bacteroidia;__Bacteroidales;__S24-7;__g
EF096498	12.6707903	0.00177245	0.05258272	5.5	2.16666667	28	Bacteria;__Bacteroidetes;__Bacteroidia;__Bacteroidales;__S24-7;__g
EU505062	12.6334683	0.00180583	0.05258272	113.75	14.1666667	166	Bacteria;__Bacteroidetes;__Bacteroidia;__Bacteroidales;__S24-7;__g
EF406778	12.537092	0.00189498	0.05258272	3.75	17.5	104.166667	Bacteria;__Bacteroidetes;__Bacteroidia;__Bacteroidales;__S24-7;__g
EF406534	12.4631268	0.00196638	0.05258272	19.75	9.33333333	100	Bacteria;__Bacteroidetes;__Bacteroidia;__Bacteroidales;__S24-7;__g
EF097069	12.1416526	0.00230926	0.05258272	12.75	1	0	Bacteria;__Bacteroidetes;__Bacteroidia;__Bacteroidales;__S24-7;__g
EF40678	12.09499	0.002363	0.052582	28.5	11.5	118	Bacteria;__Bacteroidetes;__Bacteroidia;__Bacteroidales;__S24-7;__g

9	26	77	72				
EU454618	12.0949926	0.00236377	0.05258272	134.75	36	98.1666667	Bacteria;__Bacteroidetes;__Bacteroidia;__Bacteroidales;__S24-7;__g
EF406754	12.0322327	0.00243912	0.05258272	1.25	0.16666667	3.5	Bacteria;__Bacteroidetes;__Bacteroidia;__Bacteroidales;__S24-7;__g
EU451465	11.9899818	0.0024912	0.05258272	0	0.16666667	1.83333333	Bacteria;__Bacteroidetes;__Bacteroidia;__Bacteroidales;__S24-7;__g
EF097335	11.9868713	0.00249508	0.05258272	13.5	2.33333333	21.6666667	Bacteria;__Bacteroidetes;__Bacteroidia;__Bacteroidales;__S24-7;__g
FJ879490	11.9106317	0.00259202	0.05258272	0	2.16666667	6.16666667	Bacteria;__Bacteroidetes;__Bacteroidia;__Bacteroidales;__S24-7;__g
EU451403	11.7557177	0.00280078	0.05258272	1.75	3.5	0	Bacteria;__Bacteroidetes;__Bacteroidia;__Bacteroidales;__S24-7;__g
EF097483	11.6653727	0.00293019	0.05258272	21.25	19.3333333	0	Bacteria;__Bacteroidetes;__Bacteroidia;__Bacteroidales;__S24-7;__g
EU504830	11.6144068	0.00300582	0.05258272	3.5	1	0	Bacteria;__Bacteroidetes;__Bacteroidia;__Bacteroidales;__S24-7;__g
EF096989	11.4728682	0.00322625	0.05258272	104.5	86.6666667	0	Bacteria;__Bacteroidetes;__Bacteroidia;__Bacteroidales;__S24-7;__g
EU455595	11.4490741	0.00326486	0.05258272	10	4	41.1666667	Bacteria;__Bacteroidetes;__Bacteroidia;__Bacteroidales;__S24-7;__g
EU455919	11.4490741	0.00326486	0.05258272	8.5	4.66666667	46.6666667	Bacteria;__Bacteroidetes;__Bacteroidia;__Bacteroidales;__S24-7;__g
EU504474	11.4356532	0.00328685	0.05258272	24	0	12	Bacteria;__Bacteroidetes;__Bacteroidia;__Bacteroidales;__S24-7;__g
EU455284	11.3171642	0.00348746	0.05258272	32.25	7.83333333	15	Bacteria;__Bacteroidetes;__Bacteroidia;__Bacteroidales;__S24-7;__g
EF099832	10.9170306	0.00425988	0.05258272	0	2	0	Bacteria;__Bacteroidetes;__Bacteroidia;__Bacteroidales;__S24-7;__g
EU454128	10.8932462	0.00431084	0.05258272	0	2.33333333	0	Bacteria;__Bacteroidetes;__Bacteroidia;__Bacteroidales;__S24-7;__g
EU505419	10.8932462	0.00431084	0.05258272	0	0	3.16666667	Bacteria;__Bacteroidetes;__Bacteroidia;__Bacteroidales;__S24-7;__g
EU504508	10.8857304	0.00432707	0.05258272	1	13.1666667	1.16666667	Bacteria;__Bacteroidetes;__Bacteroidia;__Bacteroidales;__S24-7;__g
AM932633	10.8695652	0.00436218	0.05258272	0	54.8333333	0	Bacteria;__Bacteroidetes;__Bacteroidia;__Bacteroidales;__S24-7;__g
EU791064	10.8695652	0.00436218	0.05258272	0	10.3333333	0	Bacteria;__Bacteroidetes;__Bacteroidia;__Bacteroidales;__S24-7;__g
EF406401	10.8655275	0.004371	0.05258272	14	16.8333333	0.83333333	Bacteria;__Bacteroidetes;__Bacteroidia;__Bacteroidales;__S24-7;__g
EF096984	10.7536765	0.00462241	0.05464042	37	147.5	55	Bacteria;__Bacteroidetes;__Bacteroidia;__Bacteroidales;__S24-7;__g
EF406854	10.6934033	0.00476384	0.05578588	10.25	4.16666667	0.83333333	Bacteria;__Bacteroidetes;__Bacteroidia;__Bacteroidales;__S24-7;__g
EU457778	10.3846154	0.00555916	0.06332392	16.75	5	0	Bacteria;__Bacteroidetes;__Bacteroidia;__Bacteroidales;__S24-7;__g
EF096846	10.3220612	0.00573579	0.06412785	2.25	4.16666667	0	Bacteria;__Bacteroidetes;__Bacteroidia;__Bacteroidales;__S24-7;__g

EU5046 03	10.28571 43	0.005840 98	0.064127 85	1.75	0	0	Bacteria;__Bacteroidetes;__Bacteroidia;__Bacteroidales;__S24-7;__g
EU7909 90	10.25316 46	0.005936 82	0.064127 85	3.5	0	0	Bacteria;__Bacteroidetes;__Bacteroidia;__Bacteroidales;__S24-7;__g
EF09920 3	10.10846 22	0.006382 27	0.068350 32	0.75	0	1.6666666 7	Bacteria;__Bacteroidetes;__Bacteroidia;__Bacteroidales;__S24-7;__g
EF09751 0	10.01956 18	0.006672 37	0.070256 08	0	1.16666667	4	Bacteria;__Bacteroidetes;__Bacteroidia;__Bacteroidales;__S24-7;__g
EF60304 0	9.971153 85	0.006835 83	0.071377 49	2.75	1.33333333	0	Bacteria;__Bacteroidetes;__Bacteroidia;__Bacteroidales;__S24-7;__g
EF09746 5	9.889705 88	0.007119 96	0.073729 85	3.25	2.66666667	0	Bacteria;__Bacteroidetes;__Bacteroidia;__Bacteroidales;__S24-7;__g
EF09819 5	9.827330 51	0.007345 52	0.075442 06	3	1	0.6666666 7	Bacteria;__Bacteroidetes;__Bacteroidia;__Bacteroidales;__S24-7;__g
EF09723 8	9.742702 1	0.007663 01	0.077899 22	9.5	9.16666667	0.5	Bacteria;__Bacteroidetes;__Bacteroidia;__Bacteroidales;__S24-7;__g
EU5046 47	9.694852 94	0.007848 55	0.078049 47	169.5	325.833333	137.66666 7	Bacteria;__Bacteroidetes;__Bacteroidia;__Bacteroidales;__S24-7;__g
EF09783 7	9.649493 24	0.008028 59	0.078592 35	3.75	1.5	0	Bacteria;__Bacteroidetes;__Bacteroidia;__Bacteroidales;__S24-7;__g
EF09713 5	9.597423 51	0.008240 36	0.079424 35	0	0.83333333	2.1666666 7	Bacteria;__Bacteroidetes;__Bacteroidia;__Bacteroidales;__S24-7;__g
EU5050 87	9.495759 59	0.008670 06	0.081173 58	25.75	49.1666667	20.166666 7	Bacteria;__Bacteroidetes;__Bacteroidia;__Bacteroidales;__S24-7;__g
EF09711 2	9.298245 61	0.009569 99	0.085003 73	0.25	0	3	Bacteria;__Bacteroidetes;__Bacteroidia;__Bacteroidales;__S24-7;__g
EF09705 2	9.298245 61	0.009569 99	0.085003 73	0.25	3	0	Bacteria;__Bacteroidetes;__Bacteroidia;__Bacteroidales;__S24-7;__g
EF09757 7	9.239130 43	0.009857 08	0.085003 73	9.5	6.83333333	0	Bacteria;__Bacteroidetes;__Bacteroidia;__Bacteroidales;__S24-7;__g
EU5045 69	9.239130 43	0.009857 08	0.085003 73	33.5	19.6666667	0	Bacteria;__Bacteroidetes;__Bacteroidia;__Bacteroidales;__S24-7;__g
EU5050 67	9.200892 86	0.010047 35	0.085003 73	4	26.5	0	Bacteria;__Bacteroidetes;__Bacteroidia;__Bacteroidales;__S24-7;__g
DQ0149 20	9.179090 21	0.010157 48	0.085003 73	1.5	5.16666667	2.3333333 3	Bacteria;__Bacteroidetes;__Bacteroidia;__Bacteroidales;__S24-7;__g
EF09993 7	9.152173 91	0.010295 1	0.085003 73	2.25	0.16666667	0.5	Bacteria;__Bacteroidetes;__Bacteroidia;__Bacteroidales;__S24-7;__g
AM9325 66	9.137134 05	0.010372 81	0.085003 73	8.25	2	0.5	Bacteria;__Bacteroidetes;__Bacteroidia;__Bacteroidales;__S24-7;__g
EF40646 2	9.041889 48	0.010878 74	0.086546 91	0.75	0.16666667	1.8333333 3	Bacteria;__Bacteroidetes;__Bacteroidia;__Bacteroidales;__S24-7;__g
EU5041 42	9.035539 22	0.010913 34	0.086546 91	0.25	0	1.5	Bacteria;__Bacteroidetes;__Bacteroidia;__Bacteroidales;__S24-7;__g
EU5081 78	9.000224 42	0.011107 75	0.087534 66	0.75	0	2.6666666 7	Bacteria;__Bacteroidetes;__Bacteroidia;__Bacteroidales;__S24-7;__g
EF09802 8	8.816831 68	0.012174 45	0.095341 16	0.25	1.33333333	0	Bacteria;__Bacteroidetes;__Bacteroidia;__Bacteroidales;__S24-7;__g
JQ08395	9.730707	0.007709	0.077899	5.75	7.33333333	0	Bacteria;__Cyanobacteria;__4C0d-2;__o;__f;__g

3	4	1	22				
DQ823870	8.79279279	0.01232166	0.09589468	0.5	0	2	Bacteria;__Cyanobacteria;__4C0d-2;__o;__f;__g
EU452206	12.2743682	0.002161	0.05258272	0	2	0.16666667	Bacteria;__Firmicutes;__Bacilli;__Lactobacillales;__Lactobacillaceae;__Lactobacillus
EU455551	12.2617102	0.00217472	0.05258272	12.25	0.16666667	0	Bacteria;__Firmicutes;__Bacilli;__Lactobacillales;__Lactobacillaceae;__Lactobacillus
EU505198	11.878882	0.0026335	0.05258272	83.5	32.6666667	0	Bacteria;__Firmicutes;__Bacilli;__Lactobacillales;__Lactobacillaceae;__Lactobacillus
EU505261	11.8604651	0.00265786	0.05258272	249.75	92.1666667	0	Bacteria;__Firmicutes;__Bacilli;__Lactobacillales;__Lactobacillaceae;__Lactobacillus
DQ815810	11.6946373	0.00288763	0.05258272	0.25	0.83333333	10.3333333	Bacteria;__Firmicutes;__Bacilli;__Lactobacillales;__Lactobacillaceae;__Lactobacillus
EU452861	11.5915916	0.00304031	0.05258272	0	10.6666667	3.16666667	Bacteria;__Firmicutes;__Bacilli;__Lactobacillales;__Lactobacillaceae;__Lactobacillus
EF097925	11.5731755	0.00306843	0.05258272	37.75	15.5	0	Bacteria;__Firmicutes;__Bacilli;__Lactobacillales;__Lactobacillaceae;__Lactobacillus
HM363537	11.545082	0.00311184	0.05258272	0	0.5	4	Bacteria;__Firmicutes;__Bacilli;__Lactobacillales;__Lactobacillaceae;__Lactobacillus
EU453643	11.25	0.00360656	0.05258272	0.5	0.5	5.33333333	Bacteria;__Firmicutes;__Bacilli;__Lactobacillales;__Lactobacillaceae;__Lactobacillus
EU508795	11.1129032	0.00386246	0.05258272	8.25	2.66666667	0	Bacteria;__Firmicutes;__Bacilli;__Lactobacillales;__Lactobacillaceae;__Lactobacillus
EU451376	11.1115326	0.00386511	0.05258272	0	7.16666667	2	Bacteria;__Firmicutes;__Bacilli;__Lactobacillales;__Lactobacillaceae;__Lactobacillus
EU507317	9.41789216	0.00901427	0.08244441	3.25	0.83333333	0	Bacteria;__Firmicutes;__Bacilli;__Lactobacillales;__Lactobacillaceae;__Lactobacillus
EU452856	9.23664122	0.00986936	0.08500373	0	10.5	2.83333333	Bacteria;__Firmicutes;__Bacilli;__Lactobacillales;__Lactobacillaceae;__Lactobacillus
EU453156	9.19452888	0.01007937	0.08500373	0	8.66666667	2.5	Bacteria;__Firmicutes;__Bacilli;__Lactobacillales;__Lactobacillaceae;__Lactobacillus
EU504925	10.3120125	0.00576468	0.06412785	3	0.5	9.33333333	Bacteria;__Firmicutes;__Clostridia;__Clostridiales;__Clostridiaceae;__Candidatus_Arthromitus
EU454563	12.4789207	0.00195091	0.05258272	0	0.33333333	3.83333333	Bacteria;__Firmicutes;__Clostridia;__Clostridiales;__Family_XIII_Incertae_Sedis;__Incertae_Sedis
EF098507	14.0900196	0.00087175	0.0501624	0	3.33333333	0	Bacteria;__Firmicutes;__Clostridia;__Clostridiales;__Lachnospiraceae;__g
EU505679	11.417896	0.00331616	0.05258272	5	0	6.83333333	Bacteria;__Firmicutes;__Clostridia;__Clostridiales;__Lachnospiraceae;__g
EF096337	11.2939453	0.00352818	0.05258272	7.75	7.33333333	0	Bacteria;__Firmicutes;__Clostridia;__Clostridiales;__Lachnospiraceae;__g
EU474250	11.2009804	0.00369605	0.05258272	2	0	0.33333333	Bacteria;__Firmicutes;__Clostridia;__Clostridiales;__Lachnospiraceae;__g
EU505540	10.9500805	0.00419006	0.05258272	2	0	4	Bacteria;__Firmicutes;__Clostridia;__Clostridiales;__Lachnospiraceae;__g
DQ014815	10.8932462	0.00431084	0.05258272	0	3	0	Bacteria;__Firmicutes;__Clostridia;__Clostridiales;__Lachnospiraceae;__g
EU509807	10.8695652	0.00436218	0.05258272	0	34	0	Bacteria;__Firmicutes;__Clostridia;__Clostridiales;__Lachnospiraceae;__g

JQ083865	10.8695652	0.00436218	0.05258272	0	100.166667	0	Bacteria;__Firmicutes;__Clostridia;__Clostridiales;__Lachnospiraceae;__g
AY991187	10.8695652	0.00436218	0.05258272	0	0	11.8333333	Bacteria;__Firmicutes;__Clostridia;__Clostridiales;__Lachnospiraceae;__g
EU510232	10.2531646	0.00593682	0.06412785	8.5	0	0	Bacteria;__Firmicutes;__Clostridia;__Clostridiales;__Lachnospiraceae;__g
EU511665	10.2531646	0.00593682	0.06412785	6	0	0	Bacteria;__Firmicutes;__Clostridia;__Clostridiales;__Lachnospiraceae;__g
DQ014717	9.67412451	0.00793032	0.07824163	7.5	1	0	Bacteria;__Firmicutes;__Clostridia;__Clostridiales;__Lachnospiraceae;__g
EU505388	9.57940252	0.00831494	0.07953146	2.75	0.16666667	5.33333333	Bacteria;__Firmicutes;__Clostridia;__Clostridiales;__Lachnospiraceae;__g
EU474414	9.49324324	0.00868097	0.08117358	21.25	3.83333333	1.83333333	Bacteria;__Firmicutes;__Clostridia;__Clostridiales;__Lachnospiraceae;__g
EU510011	9.36125212	0.00927321	0.08419803	1.25	0	2.66666667	Bacteria;__Firmicutes;__Clostridia;__Clostridiales;__Lachnospiraceae;__g
EU454936	9.28015564	0.00965695	0.08500373	0.25	7.83333333	0	Bacteria;__Firmicutes;__Clostridia;__Clostridiales;__Lachnospiraceae;__g
EU474212	9.14196568	0.01034778	0.08500373	24	8.66666667	0.33333333	Bacteria;__Firmicutes;__Clostridia;__Clostridiales;__Lachnospiraceae;__g
HQ740257	9.08900394	0.01062546	0.08589487	0.25	0	1.33333333	Bacteria;__Firmicutes;__Clostridia;__Clostridiales;__Lachnospiraceae;__g
EU509679	9.03553922	0.01091334	0.08654691	0.25	4.16666667	0	Bacteria;__Firmicutes;__Clostridia;__Clostridiales;__Lachnospiraceae;__g
EU509978	14.7692308	0.00062073	0.0501624	3	0	0	Bacteria;__Firmicutes;__Clostridia;__Clostridiales;__Lachnospiraceae;__Incertae_Sedis
EU455469	10.9147351	0.00426477	0.05258272	1.5	2.83333333	0	Bacteria;__Firmicutes;__Clostridia;__Clostridiales;__Lachnospiraceae;__Incertae_Sedis
AY991185	14.6564885	0.00065673	0.0501624	6.75	0	0	Bacteria;__Firmicutes;__Clostridia;__Clostridiales;__Ruminococcaceae;__g
EU503721	11.7370543	0.00282703	0.05258272	0.75	2	0	Bacteria;__Firmicutes;__Clostridia;__Clostridiales;__Ruminococcaceae;__g
EF406693	11.0662359	0.00395364	0.05258272	0	0.66666667	7.16666667	Bacteria;__Firmicutes;__Clostridia;__Clostridiales;__Ruminococcaceae;__g
EU467603	10.9836703	0.00412028	0.05258272	0	2.16666667	11.1666667	Bacteria;__Firmicutes;__Clostridia;__Clostridiales;__Ruminococcaceae;__g
EU510020	10.9285714	0.00423537	0.05258272	1.5	0	8.83333333	Bacteria;__Firmicutes;__Clostridia;__Clostridiales;__Ruminococcaceae;__g
EU506367	9.60724708	0.00819998	0.07942435	0.25	3.83333333	0	Bacteria;__Firmicutes;__Clostridia;__Clostridiales;__Ruminococcaceae;__g
EU505220	9.125	0.01043594	0.08500373	2	3	0.16666667	Bacteria;__Firmicutes;__Clostridia;__Clostridiales;__Ruminococcaceae;__g
FJ680831	9.12280702	0.01044739	0.08500373	0	2.83333333	0.16666667	Bacteria;__Firmicutes;__Clostridia;__Clostridiales;__Ruminococcaceae;__g
AY993790	14.0077821	0.00090834	0.0501624	0	0	7.16666667	Bacteria;__Firmicutes;__Clostridia;__Clostridiales;__Ruminococcaceae;__Incertae_Sedis
EU507363	10.9649123	0.0041591	0.05258272	0	0	1.33333333	Bacteria;__Firmicutes;__Clostridia;__Clostridiales;__Ruminococcaceae;__Incertae_Sedis
EF60378	10.89324	0.004310	0.052582	0	0	4	Bacteria;__Firmicutes;__Clostridia;__Clostridiales;__Ruminococcaceae;__Incertae_Sedis

1	62	84	72				
EF098509	10.0309598	0.00663445	0.07025608	8.5	2.16666667	0.33333333	Bacteria;__Firmicutes;__Clostridia;__Clostridiales;__Ruminococcaceae;__Incertae_Sedis
EF096640	9.17578125	0.0101743	0.08500373	0.75	15.1666667	0.5	Bacteria;__Firmicutes;__Clostridia;__Clostridiales;__Ruminococcaceae;__Oscillibacter
EU457075	10.989011	0.00410929	0.05258272	0	0	1.16666667	Bacteria;__Firmicutes;__Clostridia;__Clostridiales;__uncultured;__g
DQ815799	11.7580645	0.00279749	0.05258272	1.75	0	10.1666667	Bacteria;__Firmicutes;__Erysipelotrichi;__Erysipelotrichales;__Erysipelotrichaceae;__g
JQ083859	11.7288136	0.00283871	0.05258272	0.75	0	5	Bacteria;__Firmicutes;__Erysipelotrichi;__Erysipelotrichales;__Erysipelotrichaceae;__g
AB606407	11.0241503	0.00403772	0.05258272	0	0.83333333	7	Bacteria;__Firmicutes;__Erysipelotrichi;__Erysipelotrichales;__Erysipelotrichaceae;__g
EU508251	9.71319133	0.00777691	0.07795579	9	5.66666667	36.8333333	Bacteria;__Firmicutes;__Erysipelotrichi;__Erysipelotrichales;__Erysipelotrichaceae;__g
AM932629	9.47324102	0.00876823	0.08138215	10	6.33333333	35.5	Bacteria;__Firmicutes;__Erysipelotrichi;__Erysipelotrichales;__Erysipelotrichaceae;__Incertae_Sedis
AM932646	10.8695652	0.00436218	0.05258272	0	12.6666667	0	Bacteria;__Proteobacteria;__Deltaproteobacteria;__Desulfovibrionales;__Desulfovibrionaceae;__Desulfovibrio
EU457232	10.4399323	0.00540751	0.06216158	0	3.33333333	0.5	Bacteria;__Proteobacteria;__Deltaproteobacteria;__Desulfovibrionales;__Desulfovibrionaceae;__Desulfovibrio
AY990042	14.0077821	0.00090834	0.0501624	0	0	25.1666667	Bacteria;__Proteobacteria;__Epsilonproteobacteria;__Campylobacterales;__Helicobacteraceae;__Helicobacter
AM932639	10.5254516	0.00518116	0.06011108	1.25	2	0	Bacteria;__Tenericutes;__Mollicutes;__Anaeroplasmatales;__Anaeroplasmataceae;__Anaeroplasma
EU939405	11.2403101	0.00362408	0.05258272	38	0	54.1666667	Bacteria;__Verrucomicrobia;__Verrucomicrobiae;__Verrucomicrobiales;__Verrucomicrobiaceae;__Akkermansia
DQ805303	11.2403101	0.00362408	0.05258272	86	0	122.666667	Bacteria;__Verrucomicrobia;__Verrucomicrobiae;__Verrucomicrobiales;__Verrucomicrobiaceae;__Akkermansia
EU507486	9.54166667	0.00847332	0.08043231	1	0	1.33333333	Bacteria;__Verrucomicrobia;__Verrucomicrobiae;__Verrucomicrobiales;__Verrucomicrobiaceae;__Akkermansia
EU775014	9.44947665	0.00887304	0.08174936	3.25	0	3.5	Bacteria;__Verrucomicrobia;__Verrucomicrobiae;__Verrucomicrobiales;__Verrucomicrobiaceae;__Akkermansia

*Graphically represented in Supplementary Figure 2e.

Supplementary Table 5. Pathology H2 Congenics (Figure 2e-h) Sifnificant differences by OTU*

OTU	Test-	P	FDR_P	BB	DD	KK	taxonomy

	Statistic			mean	mean	mean	
New.CleanUp.ReferenceOTU1285	9.91150442	0.00704278	0.07089587	3	0	0	k__Bacteria
228798	9.85185185	0.007256	0.07089587	10	0	12.2	k__Bacteria
1571092	9.48158845	0.00873171	0.07089587	17	0.4	30.4	k__Bacteria
3176547	7.64155844	0.02191072	0.0953027	10.8	0.6	41.6	k__Bacteria
New.CleanUp.ReferenceOTU134143	13.2911392	0.00129977	0.07089587	0	0	6.4	k__Bacteria;p__Actinobacteria;c__Actinobacteria;o__Bifidobacteriales;f__Bifidobacteriaceae;g__Bifidobacterium
681370	11.0352941	0.00401528	0.07089587	0.2	1.2	106	k__Bacteria;p__Actinobacteria;c__Actinobacteria;o__Bifidobacteriales;f__Bifidobacteriaceae;g__Bifidobacterium;s__pseudolongum
New.CleanUp.ReferenceOTU212230	8.32182741	0.0155933	0.08141434	2.6	0	0.2	k__Bacteria;p__Bacteroidetes;c__Bacteroidia;o__Bacteroidales
New.ReferenceOTU969	7.65726316	0.02173934	0.09512074	0	15	1	k__Bacteria;p__Bacteroidetes;c__Bacteroidia;o__Bacteroidales
New.ReferenceOTU467	7.54328358	0.02301425	0.09791994	0.4	9.6	2.6	k__Bacteria;p__Bacteroidetes;c__Bacteroidia;o__Bacteroidales
New.CleanUp.ReferenceOTU120492	11.0518248	0.00398223	0.07089587	0.6	25.4	4.6	k__Bacteria;p__Bacteroidetes;c__Bacteroidia;o__Bacteroidales;f__Bacteroidaceae;g__Bacteroides
181719	10.1030411	0.00639959	0.07089587	8.6	177.6	44	k__Bacteria;p__Bacteroidetes;c__Bacteroidia;o__Bacteroidales;f__Bacteroidaceae;g__Bacteroides;s__
162539	13.2911392	0.00129977	0.07089587	0	0	15.4	k__Bacteria;p__Bacteroidetes;c__Bacteroidia;o__Bacteroidales;f__Rikenellaceae
New.ReferenceOTU1402	7.75555556	0.02069677	0.09263873	0	3.6	22.2	k__Bacteria;p__Bacteroidetes;c__Bacteroidia;o__Bacteroidales;f__Rikenellaceae;g__Rikenella;s__
215897	13.2911392	0.00129977	0.07089587	8.4	0	0	k__Bacteria;p__Bacteroidetes;c__Bacteroidia;o__Bacteroidales;f__S24-7;g__;s__
New.CleanUp.ReferenceOTU76772	11.7050228	0.00287268	0.07089587	3.2	0.2	0	k__Bacteria;p__Bacteroidetes;c__Bacteroidia;o__Bacteroidales;f__S24-7;g__;s__
New.ReferenceOTU190	11.6783599	0.00291123	0.07089587	12	0	0.2	k__Bacteria;p__Bacteroidetes;c__Bacteroidia;o__Bacteroidales;f__S24-7;g__;s__
New.ReferenceOTU736	11.0526316	0.00398063	0.07089587	16.6	0.2	0.2	k__Bacteria;p__Bacteroidetes;c__Bacteroidia;o__Bacteroidales;f__S24-7;g__;s__
162025	10.467148	0.00533443	0.07089587	82.2	0.4	11	k__Bacteria;p__Bacteroidetes;c__Bacteroidia;o__Bacteroidales;f__S24-7;g__;s__
261350	10.0606526	0.00653668	0.07089587	76.4	1.6	0.2	k__Bacteria;p__Bacteroidetes;c__Bacteroidia;o__Bacteroidales;f__S24-7;g__;s__
174805	9.91150442	0.00704278	0.07089587	4	0	0	k__Bacteria;p__Bacteroidetes;c__Bacteroidia;o__Bacteroidales;f__S24-7;g__;s__
177533	9.91150442	0.00704278	0.07089587	7.8	0	0	k__Bacteria;p__Bacteroidetes;c__Bacteroidia;o__Bacteroidales;f__S24-7;g__;s__
New.ReferenceOTU1265	9.91150442	0.00704278	0.07089587	6.6	0	0	k__Bacteria;p__Bacteroidetes;c__Bacteroidia;o__Bacteroidales;f__S24-7;g__;s__
New.ReferenceOTU1677	9.91150442	0.00704278	0.07089587	2	0	0	k__Bacteria;p__Bacteroidetes;c__Bacteroidia;o__Bacteroidales;f__S24-7;g__;s__
New.ReferenceOTU1642	9.91150	0.00704	0.07089	4	0	0	k__Bacteria;p__Bacteroidetes;c__Bacteroidia;o__Bacteroidales;f__S24-7;g__;s__

	442	278	587				
New.ReferenceOTU1144	9.91150442	0.00704278	0.07089587	4.4	0	0	k__Bacteria;p__Bacteroidetes;c__Bacteroidia;o__Bacteroidales;f__S24-7;g__;s__
New.ReferenceOTU1526	9.88235294	0.00714619	0.07089587	6.6	0	0	k__Bacteria;p__Bacteroidetes;c__Bacteroidia;o__Bacteroidales;f__S24-7;g__;s__
New.CleanUp.ReferenceOTU206210	9.88235294	0.00714619	0.07089587	6.8	0	0	k__Bacteria;p__Bacteroidetes;c__Bacteroidia;o__Bacteroidales;f__S24-7;g__;s__
2212505	9.84179104	0.0072926	0.07089587	158.8	1.6	0.8	k__Bacteria;p__Bacteroidetes;c__Bacteroidia;o__Bacteroidales;f__S24-7;g__;s__
New.ReferenceOTU519	8.89113924	0.01173042	0.07676224	1.4	15.6	4	k__Bacteria;p__Bacteroidetes;c__Bacteroidia;o__Bacteroidales;f__S24-7;g__;s__
208434	8.72230216	0.01276369	0.07847564	38.2	111	31.6	k__Bacteria;p__Bacteroidetes;c__Bacteroidia;o__Bacteroidales;f__S24-7;g__;s__
2435303	8.30075949	0.01575843	0.08141434	17.8	0.2	0	k__Bacteria;p__Bacteroidetes;c__Bacteroidia;o__Bacteroidales;f__S24-7;g__;s__
New.ReferenceOTU627	8.30075949	0.01575843	0.08141434	6.2	0	0.2	k__Bacteria;p__Bacteroidetes;c__Bacteroidia;o__Bacteroidales;f__S24-7;g__;s__
New.ReferenceOTU63	8.12954545	0.01716689	0.08431842	8.2	1.8	0.2	k__Bacteria;p__Bacteroidetes;c__Bacteroidia;o__Bacteroidales;f__S24-7;g__;s__
New.CleanUp.ReferenceOTU90984	7.65524297	0.02176131	0.09512074	0	1.4	0.2	k__Bacteria;p__Bacteroidetes;c__Bacteroidia;o__Bacteroidales;f__S24-7;g__;s__
New.ReferenceOTU685	8.98074074	0.01121649	0.07560995	0.8	14	2	k__Bacteria;p__Firmicutes
418334	8.16443595	0.01687001	0.08390017	4.8	0	4.8	k__Bacteria;p__Firmicutes
637934	7.51832061	0.0233033	0.09877421	13.8	0.4	31.6	k__Bacteria;p__Firmicutes
318764	10.4437956	0.00539708	0.07089587	0.8	12.6	1.6	k__Bacteria;p__Firmicutes;c__Bacilli;o__Lactobacillales;f__Lactobacillaceae;g__Lactobacillus
509149	9.45393258	0.00885329	0.07089587	1.2	7.2	0.6	k__Bacteria;p__Firmicutes;c__Bacilli;o__Lactobacillales;f__Lactobacillaceae;g__Lactobacillus
New.ReferenceOTU1373	7.70126582	0.02126627	0.09405913	3.2	12.8	3.2	k__Bacteria;p__Firmicutes;c__Bacilli;o__Lactobacillales;f__Lactobacillaceae;g__Lactobacillus
4397402	10.3610687	0.005625	0.07089587	2.4	184.4	1.4	k__Bacteria;p__Firmicutes;c__Bacilli;o__Lactobacillales;f__Lactobacillaceae;g__Lactobacillus;s__
4428313	9.56834532	0.00836104	0.07089587	81.4	1.4	54.2	k__Bacteria;p__Firmicutes;c__Bacilli;o__Lactobacillales;f__Lactobacillaceae;g__Lactobacillus;s__
New.ReferenceOTU1518	11.4704545	0.00323015	0.07089587	0.6	8.2	2.6	k__Bacteria;p__Firmicutes;c__Bacilli;o__Lactobacillales;f__Lactobacillaceae;g__Lactobacillus;s__vaginalis
New.ReferenceOTU962	9.19064748	0.01009895	0.07141835	5.2	23.8	9.4	k__Bacteria;p__Firmicutes;c__Bacilli;o__Lactobacillales;f__Lactobacillaceae;g__Lactobacillus;s__vaginalis
214919	10.280916	0.00585501	0.07089587	1	1	62.6	k__Bacteria;p__Firmicutes;c__Bacilli;o__Turicibacterales;f__Turicibacteraceae;g__Turicibacter;s__
234912	13.3248731	0.00127803	0.07089587	0	0	8.4	k__Bacteria;p__Firmicutes;c__Clostridia;o__Clostridiales
195711	13.2911392	0.00129977	0.07089587	0	0	41	k__Bacteria;p__Firmicutes;c__Clostridia;o__Clostridiales
260156	12.118451	0.00233621	0.07089587	0.4	0	9.6	k__Bacteria;p__Firmicutes;c__Clostridia;o__Clostridiales

293754	12.0909091	0.0023686	0.07089587	0	0.2	15.6	k__Bacteria;p__Firmicutes;c__Clostridia;o__Clostridiales
337407	11.7050228	0.00287268	0.07089587	0.2	0	6.6	k__Bacteria;p__Firmicutes;c__Clostridia;o__Clostridiales
215018	11.6898608	0.00289454	0.07089587	1	0	11.4	k__Bacteria;p__Firmicutes;c__Clostridia;o__Clostridiales
261590	11.6176471	0.00300096	0.07089587	0.6	0	128.4	k__Bacteria;p__Firmicutes;c__Clostridia;o__Clostridiales
New.ReferenceOTU717	11.508785	0.00316883	0.07089587	1.8	0.6	13.6	k__Bacteria;p__Firmicutes;c__Clostridia;o__Clostridiales
New.CleanUp.ReferenceOTU137408	11.2254545	0.0036511	0.07089587	1.6	9.6	0	k__Bacteria;p__Firmicutes;c__Clostridia;o__Clostridiales
New.ReferenceOTU1426	11.2051376	0.00368838	0.07089587	3.4	0.2	12.2	k__Bacteria;p__Firmicutes;c__Clostridia;o__Clostridiales
174824	11.1596396	0.00377325	0.07089587	69.8	361.6	1.2	k__Bacteria;p__Firmicutes;c__Clostridia;o__Clostridiales
635059	11.0759494	0.00393449	0.07089587	0.2	0.2	9.8	k__Bacteria;p__Firmicutes;c__Clostridia;o__Clostridiales
New.ReferenceOTU760	10.9724008	0.00414356	0.07089587	1.2	0.2	7.4	k__Bacteria;p__Firmicutes;c__Clostridia;o__Clostridiales
322564	10.8091778	0.0044959	0.07089587	3.8	0.2	48.8	k__Bacteria;p__Firmicutes;c__Clostridia;o__Clostridiales
New.ReferenceOTU64	10.7493562	0.00463241	0.07089587	4	0	0.8	k__Bacteria;p__Firmicutes;c__Clostridia;o__Clostridiales
New.ReferenceOTU802	10.6664151	0.00482856	0.07089587	2	0	3	k__Bacteria;p__Firmicutes;c__Clostridia;o__Clostridiales
275138	10.64	0.00489275	0.07089587	0.2	0.4	12.8	k__Bacteria;p__Firmicutes;c__Clostridia;o__Clostridiales
173939	10.4429423	0.00539938	0.07089587	2.6	0	15.8	k__Bacteria;p__Firmicutes;c__Clostridia;o__Clostridiales
307170	10.3120721	0.0057645	0.07089587	17.4	0.6	32	k__Bacteria;p__Firmicutes;c__Clostridia;o__Clostridiales
195433	10.280916	0.00585501	0.07089587	0.6	1	22.2	k__Bacteria;p__Firmicutes;c__Clostridia;o__Clostridiales
335632	10.2726115	0.00587937	0.07089587	6	0.2	0.2	k__Bacteria;p__Firmicutes;c__Clostridia;o__Clostridiales
New.ReferenceOTU31	10.2305709	0.00600426	0.07089587	2.8	0.2	4.8	k__Bacteria;p__Firmicutes;c__Clostridia;o__Clostridiales
New.ReferenceOTU1331	10.08	0.00647375	0.07089587	1.4	7.8	0.2	k__Bacteria;p__Firmicutes;c__Clostridia;o__Clostridiales
162576	10.0768362	0.006484	0.07089587	0	9	6.6	k__Bacteria;p__Firmicutes;c__Clostridia;o__Clostridiales
198537	9.88235294	0.00714619	0.07089587	0	0	3.2	k__Bacteria;p__Firmicutes;c__Clostridia;o__Clostridiales
227967	9.88235294	0.00714619	0.07089587	0	0	18.2	k__Bacteria;p__Firmicutes;c__Clostridia;o__Clostridiales
335183	9.88015267	0.00715405	0.07089587	4.6	0.6	83.8	k__Bacteria;p__Firmicutes;c__Clostridia;o__Clostridiales
New.ReferenceOTU280	9.83376	0.00732	0.07089	14.	0.2	41.	k__Bacteria;p__Firmicutes;c__Clostridia;o__Clostridiales

	623	192	587	8		6	
176486	9.79701493	0.00745771	0.07089587	0	0.6	9.4	k__Bacteria;p__Firmicutes;c__Clostridia;o__Clostridiales
175758	9.75942029	0.00759922	0.07089587	2.8	3	271.6	k__Bacteria;p__Firmicutes;c__Clostridia;o__Clostridiales
189024	9.72740741	0.00772183	0.07089587	9.2	1.8	31.2	k__Bacteria;p__Firmicutes;c__Clostridia;o__Clostridiales
202419	9.58133874	0.0083069	0.07089587	0.2	0.4	12.2	k__Bacteria;p__Firmicutes;c__Clostridia;o__Clostridiales
174698	9.57435897	0.00833594	0.07089587	0.8	1.8	15.6	k__Bacteria;p__Firmicutes;c__Clostridia;o__Clostridiales
180944	9.55458167	0.00841878	0.07089587	1	0.2	8	k__Bacteria;p__Firmicutes;c__Clostridia;o__Clostridiales
New.ReferenceOTU1671	9.45325885	0.00885627	0.07089587	1	0.8	6.6	k__Bacteria;p__Firmicutes;c__Clostridia;o__Clostridiales
179703	9.4255814	0.00897968	0.07089587	0.8	0.4	4.6	k__Bacteria;p__Firmicutes;c__Clostridia;o__Clostridiales
332854	9.40446097	0.00907501	0.07089587	5.4	15	0.2	k__Bacteria;p__Firmicutes;c__Clostridia;o__Clostridiales
313724	9.38527828	0.00916247	0.07089587	18.6	3	6.6	k__Bacteria;p__Firmicutes;c__Clostridia;o__Clostridiales
190063	9.35555556	0.00929966	0.07127614	4.8	0	11.4	k__Bacteria;p__Firmicutes;c__Clostridia;o__Clostridiales
263899	9.32738854	0.00943156	0.07133005	1	0	4.4	k__Bacteria;p__Firmicutes;c__Clostridia;o__Clostridiales
New.ReferenceOTU1117	9.32661871	0.00943519	0.07133005	10	3.2	21	k__Bacteria;p__Firmicutes;c__Clostridia;o__Clostridiales
264359	9.29451796	0.00958785	0.07133005	0.6	4.4	1.4	k__Bacteria;p__Firmicutes;c__Clostridia;o__Clostridiales
180919	9.25659656	0.00977137	0.07133005	2	0.2	6.8	k__Bacteria;p__Firmicutes;c__Clostridia;o__Clostridiales
New.ReferenceOTU1324	9.24848485	0.00981108	0.07133005	0.6	3.2	0.8	k__Bacteria;p__Firmicutes;c__Clostridia;o__Clostridiales
New.CleanUp.ReferenceOTU85373	9.15605214	0.01027516	0.07141835	1.8	0.4	7	k__Bacteria;p__Firmicutes;c__Clostridia;o__Clostridiales
New.CleanUp.ReferenceOTU36979	9.06581741	0.01074936	0.07355839	0.4	5.4	0.2	k__Bacteria;p__Firmicutes;c__Clostridia;o__Clostridiales
356353	8.9240367	0.01153905	0.0759541	2.2	1.2	28.4	k__Bacteria;p__Firmicutes;c__Clostridia;o__Clostridiales
4364242	8.83976143	0.01203567	0.0773068	0.4	3	46.2	k__Bacteria;p__Firmicutes;c__Clostridia;o__Clostridiales
New.ReferenceOTU1270	8.7447983	0.01262092	0.07846008	2	0	6	k__Bacteria;p__Firmicutes;c__Clostridia;o__Clostridiales
4364243	8.68946322	0.01297499	0.07848116	10.2	0	27	k__Bacteria;p__Firmicutes;c__Clostridia;o__Clostridiales
271734	8.63805668	0.01331281	0.07923956	1.2	0	2.4	k__Bacteria;p__Firmicutes;c__Clostridia;o__Clostridiales
267411	8.61818182	0.01344577	0.07936454	10	0.2	31	k__Bacteria;p__Firmicutes;c__Clostridia;o__Clostridiales

267752	8.45017921	0.01462402	0.08141434	31	77.4	13.6	k__Bacteria;p__Firmicutes;c__Clostridia;o__Clostridiales
198363	8.42058824	0.014842	0.08141434	0.4	3.2	4	k__Bacteria;p__Firmicutes;c__Clostridia;o__Clostridiales
346291	8.4	0.01499558	0.08141434	33.4	16.6	0	k__Bacteria;p__Firmicutes;c__Clostridia;o__Clostridiales
New.ReferenceOTU484	8.4	0.01499558	0.08141434	19.4	0	26.2	k__Bacteria;p__Firmicutes;c__Clostridia;o__Clostridiales
229452	8.30289017	0.01574165	0.08141434	7.2	0	4.8	k__Bacteria;p__Firmicutes;c__Clostridia;o__Clostridiales
New.CleanUp.ReferenceOTU151457	8.29157175	0.01583099	0.08141434	4.8	0	5.2	k__Bacteria;p__Firmicutes;c__Clostridia;o__Clostridiales
199731	8.27891892	0.01593146	0.08141434	87	1.2	146.2	k__Bacteria;p__Firmicutes;c__Clostridia;o__Clostridiales
318819	8.27272727	0.01598086	0.08141434	3.4	8.6	0	k__Bacteria;p__Firmicutes;c__Clostridia;o__Clostridiales
274289	8.22366412	0.01637774	0.08292621	8.2	0	7.2	k__Bacteria;p__Firmicutes;c__Clostridia;o__Clostridiales
771177	8.18461538	0.01670065	0.08342869	28	0.4	28.4	k__Bacteria;p__Firmicutes;c__Clostridia;o__Clostridiales
195885	8.08230769	0.01757718	0.0855168	1.2	0.2	5.2	k__Bacteria;p__Firmicutes;c__Clostridia;o__Clostridiales
New.ReferenceOTU136	7.99198543	0.01838918	0.08756381	5.8	0.8	10.2	k__Bacteria;p__Firmicutes;c__Clostridia;o__Clostridiales
268379	7.97251908	0.01856904	0.08804558	0.2	0	6	k__Bacteria;p__Firmicutes;c__Clostridia;o__Clostridiales
172464	7.92442748	0.01902096	0.08905629	4.6	0.2	10.4	k__Bacteria;p__Firmicutes;c__Clostridia;o__Clostridiales
199674	7.86113208	0.01963256	0.09115697	5.2	0.6	30.4	k__Bacteria;p__Firmicutes;c__Clostridia;o__Clostridiales
New.ReferenceOTU554	7.81550696	0.02008557	0.09225034	1.8	8	0.4	k__Bacteria;p__Firmicutes;c__Clostridia;o__Clostridiales
317389	7.81518987	0.02008876	0.09225034	0.6	0.6	8.8	k__Bacteria;p__Firmicutes;c__Clostridia;o__Clostridiales
185923	7.77910448	0.0204545	0.09263873	0.2	0.4	4.6	k__Bacteria;p__Firmicutes;c__Clostridia;o__Clostridiales
260346	7.76202532	0.02062992	0.09263873	8	26.4	6.4	k__Bacteria;p__Firmicutes;c__Clostridia;o__Clostridiales
134065	7.66315789	0.02167536	0.09512074	1.8	0.8	6.8	k__Bacteria;p__Firmicutes;c__Clostridia;o__Clostridiales
New.ReferenceOTU1007	7.54805195	0.02295944	0.09791994	25.2	0.2	11	k__Bacteria;p__Firmicutes;c__Clostridia;o__Clostridiales
264660	13.2911392	0.00129977	0.07089587	0	13	0	k__Bacteria;p__Firmicutes;c__Clostridia;o__Clostridiales;f__;g__;s__
259870	12.118451	0.00233621	0.07089587	0.2	0	16.6	k__Bacteria;p__Firmicutes;c__Clostridia;o__Clostridiales;f__;g__;s__
174126	12.0909091	0.0023686	0.07089587	0	0.2	10.4	k__Bacteria;p__Firmicutes;c__Clostridia;o__Clostridiales;f__;g__;s__
349874	12.0909	0.00236	0.07089	1.2	0	19	k__Bacteria;p__Firmicutes;c__Clostridia;o__Clostridiales;f__;g__;s__

	091	86	587				
259820	12.0909091	0.0023686	0.07089587	0.8	0	38.6	k__Bacteria;p__Firmicutes;c__Clostridia;o__Clostridiales;f__;g__;s__
New.CleanUp.ReferenceOTU14730	11.6783599	0.00291123	0.07089587	0.4	0	7.6	k__Bacteria;p__Firmicutes;c__Clostridia;o__Clostridiales;f__;g__;s__
268063	11.4446097	0.00327216	0.07089587	6.4	0	11.6	k__Bacteria;p__Firmicutes;c__Clostridia;o__Clostridiales;f__;g__;s__
New.ReferenceOTU1136	11.2254545	0.0036511	0.07089587	2	25.8	0	k__Bacteria;p__Firmicutes;c__Clostridia;o__Clostridiales;f__;g__;s__
New.ReferenceOTU995	10.9336032	0.00422472	0.07089587	1.8	0	7.2	k__Bacteria;p__Firmicutes;c__Clostridia;o__Clostridiales;f__;g__;s__
268492	10.9013333	0.00429344	0.07089587	4.2	0.4	32	k__Bacteria;p__Firmicutes;c__Clostridia;o__Clostridiales;f__;g__;s__
New.ReferenceOTU549	10.6464491	0.004877	0.07089587	2.2	0	7.4	k__Bacteria;p__Firmicutes;c__Clostridia;o__Clostridiales;f__;g__;s__
1108453	10.6282105	0.00492168	0.07089587	22.4	0.4	0.2	k__Bacteria;p__Firmicutes;c__Clostridia;o__Clostridiales;f__;g__;s__
330296	10.4346241	0.00542188	0.07089587	0.8	0	16.4	k__Bacteria;p__Firmicutes;c__Clostridia;o__Clostridiales;f__;g__;s__
New.ReferenceOTU420	10.240481	0.00597459	0.07089587	0.8	0	4.2	k__Bacteria;p__Firmicutes;c__Clostridia;o__Clostridiales;f__;g__;s__
228043	10.1560229	0.00623229	0.07089587	4.8	0.2	22.2	k__Bacteria;p__Firmicutes;c__Clostridia;o__Clostridiales;f__;g__;s__
185177	10.1187614	0.00634949	0.07089587	60.2	0.4	107.6	k__Bacteria;p__Firmicutes;c__Clostridia;o__Clostridiales;f__;g__;s__
351309	10.1003636	0.00640817	0.07089587	114.2	0.6	213.8	k__Bacteria;p__Firmicutes;c__Clostridia;o__Clostridiales;f__;g__;s__
197706	10.0728597	0.0064969	0.07089587	28.4	0.6	43	k__Bacteria;p__Firmicutes;c__Clostridia;o__Clostridiales;f__;g__;s__
272455	10.0213333	0.00666646	0.07089587	6.8	1.2	73.4	k__Bacteria;p__Firmicutes;c__Clostridia;o__Clostridiales;f__;g__;s__
275618	9.97595628	0.00681944	0.07089587	28.2	1.2	139.8	k__Bacteria;p__Firmicutes;c__Clostridia;o__Clostridiales;f__;g__;s__
191811	9.96328872	0.00686277	0.07089587	7.2	0	17.4	k__Bacteria;p__Firmicutes;c__Clostridia;o__Clostridiales;f__;g__;s__
197568	9.96183486	0.00686776	0.07089587	2.2	38	1.4	k__Bacteria;p__Firmicutes;c__Clostridia;o__Clostridiales;f__;g__;s__
317653	9.95781818	0.00688157	0.07089587	16.8	0.4	22.2	k__Bacteria;p__Firmicutes;c__Clostridia;o__Clostridiales;f__;g__;s__
272092	9.94427481	0.00692832	0.07089587	15.4	0	30.6	k__Bacteria;p__Firmicutes;c__Clostridia;o__Clostridiales;f__;g__;s__
272619	9.91150442	0.00704278	0.07089587	0	0	7.6	k__Bacteria;p__Firmicutes;c__Clostridia;o__Clostridiales;f__;g__;s__
275796	9.9059761	0.00706227	0.07089587	1.4	0	5.6	k__Bacteria;p__Firmicutes;c__Clostridia;o__Clostridiales;f__;g__;s__
New.ReferenceOTU1039	9.82692308	0.00734701	0.07089587	8.4	0	14	k__Bacteria;p__Firmicutes;c__Clostridia;o__Clostridiales;f__;g__;s__
189585	9.7519084	0.00762781	0.07089587	31.6	0	41.2	k__Bacteria;p__Firmicutes;c__Clostridia;o__Clostridiales;f__;g__;s__

267124	9.67272727	0.00793586	0.07089587	16.2	0.4	21.8	k__Bacteria;p__Firmicutes;c__Clostridia;o__Clostridiales;f__;g__;s__
New.ReferenceOTU858	9.66307385	0.00797426	0.07089587	1.2	0	4.2	k__Bacteria;p__Firmicutes;c__Clostridia;o__Clostridiales;f__;g__;s__
258165	9.64135189	0.00806134	0.07089587	0.4	0.6	35.4	k__Bacteria;p__Firmicutes;c__Clostridia;o__Clostridiales;f__;g__;s__
3919792	9.54018018	0.00847962	0.07089587	9	109.6	9.2	k__Bacteria;p__Firmicutes;c__Clostridia;o__Clostridiales;f__;g__;s__
182712	9.4765343	0.0087538	0.07089587	30.2	0.4	23	k__Bacteria;p__Firmicutes;c__Clostridia;o__Clostridiales;f__;g__;s__
196697	9.45719626	0.00883885	0.07089587	2.2	5	0.2	k__Bacteria;p__Firmicutes;c__Clostridia;o__Clostridiales;f__;g__;s__
3868285	9.408	0.00905897	0.07089587	1.2	0.2	19.6	k__Bacteria;p__Firmicutes;c__Clostridia;o__Clostridiales;f__;g__;s__
193288	9.26666667	0.0097223	0.07133005	1.6	0.2	11.6	k__Bacteria;p__Firmicutes;c__Clostridia;o__Clostridiales;f__;g__;s__
262556	9.24663024	0.00982019	0.07133005	65	0.6	44.2	k__Bacteria;p__Firmicutes;c__Clostridia;o__Clostridiales;f__;g__;s__
267914	9.24312268	0.00983742	0.07133005	27.8	0.2	37.4	k__Bacteria;p__Firmicutes;c__Clostridia;o__Clostridiales;f__;g__;s__
184451	9.20888889	0.01000726	0.07133005	141	0.6	252.4	k__Bacteria;p__Firmicutes;c__Clostridia;o__Clostridiales;f__;g__;s__
New.ReferenceOTU402	9.20876494	0.01000788	0.07133005	1.2	0.2	8.8	k__Bacteria;p__Firmicutes;c__Clostridia;o__Clostridiales;f__;g__;s__
258812	9.14358974	0.01033939	0.07141835	8.8	0.6	40.4	k__Bacteria;p__Firmicutes;c__Clostridia;o__Clostridiales;f__;g__;s__
New.ReferenceOTU1364	9.01923077	0.01100269	0.07461825	2	0	4.6	k__Bacteria;p__Firmicutes;c__Clostridia;o__Clostridiales;f__;g__;s__
New.ReferenceOTU934	8.95551102	0.01135888	0.07571145	1	8.4	0.4	k__Bacteria;p__Firmicutes;c__Clostridia;o__Clostridiales;f__;g__;s__
New.ReferenceOTU463	8.95410526	0.01136687	0.07571145	1.2	0	4.8	k__Bacteria;p__Firmicutes;c__Clostridia;o__Clostridiales;f__;g__;s__
New.CleanUp.ReferenceOTU141476	8.87392996	0.01183179	0.07697545	1.8	0	4.4	k__Bacteria;p__Firmicutes;c__Clostridia;o__Clostridiales;f__;g__;s__
298723	8.83767535	0.01204823	0.0773068	0.6	0.2	4	k__Bacteria;p__Firmicutes;c__Clostridia;o__Clostridiales;f__;g__;s__
New.ReferenceOTU408	8.79676113	0.01229724	0.0773068	2	0.2	3.6	k__Bacteria;p__Firmicutes;c__Clostridia;o__Clostridiales;f__;g__;s__
New.ReferenceOTU1383	8.75062612	0.0125842	0.07846008	6.8	23.4	10.2	k__Bacteria;p__Firmicutes;c__Clostridia;o__Clostridiales;f__;g__;s__
New.ReferenceOTU1717	8.6912	0.01296373	0.07848116	1.6	0.2	6	k__Bacteria;p__Firmicutes;c__Clostridia;o__Clostridiales;f__;g__;s__
276580	8.65036496	0.01323114	0.07917455	9.6	0.4	13.2	k__Bacteria;p__Firmicutes;c__Clostridia;o__Clostridiales;f__;g__;s__
271219	8.60322581	0.01354669	0.07936517	1	0	3.4	k__Bacteria;p__Firmicutes;c__Clostridia;o__Clostridiales;f__;g__;s__
179265	8.57060932	0.01376943	0.08024994	29.8	5.6	83.2	k__Bacteria;p__Firmicutes;c__Clostridia;o__Clostridiales;f__;g__;s__
New.ReferenceOTU1573	8.5344	0.01402	0.08129	4.4	0	7.2	k__Bacteria;p__Firmicutes;c__Clostridia;o__Clostridiales;f__;g__;s__

		099	267				
271403	8.38235<br>294	0.01512<br>848	0.08141<br>434	2.4	0	5.8	k__Bacteria;p__Firmicutes;c__Clostridia;o__Clostridiales;f__;g__;s__
196571	8.38235<br>294	0.01512<br>848	0.08141<br>434	5.8	0	13.<br>4	k__Bacteria;p__Firmicutes;c__Clostridia;o__Clostridiales;f__;g__;s__
329528	8.30075<br>949	0.01575<br>843	0.08141<br>434	0	0.2	5.4	k__Bacteria;p__Firmicutes;c__Clostridia;o__Clostridiales;f__;g__;s__
New.ReferenceOTU803	8.29157<br>175	0.01583<br>099	0.08141<br>434	1.6	0	2.6	k__Bacteria;p__Firmicutes;c__Clostridia;o__Clostridiales;f__;g__;s__
176298	8.28458<br>781	0.01588<br>637	0.08141<br>434	162	3.2	206<br>.8	k__Bacteria;p__Firmicutes;c__Clostridia;o__Clostridiales;f__;g__;s__
276762	8.27272<br>727	0.01598<br>086	0.08141<br>434	4.8	0	4	k__Bacteria;p__Firmicutes;c__Clostridia;o__Clostridiales;f__;g__;s__
New.ReferenceOTU543	8.0864	0.01754<br>125	0.08551<br>68	2.2	0.4	6.4	k__Bacteria;p__Firmicutes;c__Clostridia;o__Clostridiales;f__;g__;s__
4424327	8.00512<br>821	0.01826<br>874	0.08756<br>381	59.<br>2	0.6	169	k__Bacteria;p__Firmicutes;c__Clostridia;o__Clostridiales;f__;g__;s__
New.ReferenceOTU287	7.96008<br>493	0.01868<br>485	0.08822<br>085	1.8	0	3	k__Bacteria;p__Firmicutes;c__Clostridia;o__Clostridiales;f__;g__;s__
1112121	7.93705<br>179	0.01890<br>128	0.08886<br>776	2	0.2	10.<br>6	k__Bacteria;p__Firmicutes;c__Clostridia;o__Clostridiales;f__;g__;s__
266091	7.90557<br>621	0.01920<br>109	0.08952<br>509	7.2	0.2	16.<br>2	k__Bacteria;p__Firmicutes;c__Clostridia;o__Clostridiales;f__;g__;s__
New.ReferenceOTU845	7.80655<br>106	0.02017<br>572	0.09225<br>034	2	0.2	4.6	k__Bacteria;p__Firmicutes;c__Clostridia;o__Clostridiales;f__;g__;s__
274374	7.78015<br>267	0.02044<br>379	0.09263<br>873	12.<br>2	0.4	20	k__Bacteria;p__Firmicutes;c__Clostridia;o__Clostridiales;f__;g__;s__
271494	7.72987<br>013	0.02096<br>428	0.09346<br>228	1.4	13.<br>4	2.4	k__Bacteria;p__Firmicutes;c__Clostridia;o__Clostridiales;f__;g__;s__
2797565	11.0301<br>471	0.00402<br>563	0.07089<br>587	2.8	0.2	5.8	k__Bacteria;p__Firmicutes;c__Clostridia;o__Clostridiales;f__[Mogibacteriaceae];g__;s__
228140	7.76168<br>582	0.02063<br>343	0.09263<br>873	2.6	10.<br>2	0.2	k__Bacteria;p__Firmicutes;c__Clostridia;o__Clostridiales;f__[Mogibacteriaceae];g__;s__
4383953	13.2911<br>392	0.00129<br>977	0.07089<br>587	0	0	96.<br>8	k__Bacteria;p__Firmicutes;c__Clostridia;o__Clostridiales;f__Clostridiaceae;g__Clostridium
189407	13.2911<br>392	0.00129<br>977	0.07089<br>587	0	0	88.<br>2	k__Bacteria;p__Firmicutes;c__Clostridia;o__Clostridiales;f__Clostridiaceae;g__SMB53;s__
196315	12.0909<br>091	0.00236<br>86	0.07089<br>587	0.2	0	24	k__Bacteria;p__Firmicutes;c__Clostridia;o__Clostridiales;f__Clostridiaceae;g__SMB53;s__
347908	13.3248<br>731	0.00127<br>803	0.07089<br>587	0	0	11.<br>2	k__Bacteria;p__Firmicutes;c__Clostridia;o__Clostridiales;f__Lachnospiraceae
3392840	12.1184<br>51	0.00233<br>621	0.07089<br>587	0.2	0	13.<br>6	k__Bacteria;p__Firmicutes;c__Clostridia;o__Clostridiales;f__Lachnospiraceae
305750	11.6783<br>599	0.00291<br>123	0.07089<br>587	0.2	0	4.6	k__Bacteria;p__Firmicutes;c__Clostridia;o__Clostridiales;f__Lachnospiraceae
320912	10.8993<br>684	0.00429<br>766	0.07089<br>587	3.4	25.<br>6	0	k__Bacteria;p__Firmicutes;c__Clostridia;o__Clostridiales;f__Lachnospiraceae
203970	10.6781<br>362	0.00480<br>034	0.07089<br>587	93.<br>4	16.<br>2	239	k__Bacteria;p__Firmicutes;c__Clostridia;o__Clostridiales;f__Lachnospiraceae

273376	10.4346241	0.00542188	0.07089587	2.8	0	0.4	k__Bacteria;p__Firmicutes;c__Clostridia;o__Clostridiales;f__Lachnospiraceae
New.ReferenceOTU1674	10.4346241	0.00542188	0.07089587	1	0	13	k__Bacteria;p__Firmicutes;c__Clostridia;o__Clostridiales;f__Lachnospiraceae
2120784	9.92727273	0.00698747	0.07089587	4.4	1	76.6	k__Bacteria;p__Firmicutes;c__Clostridia;o__Clostridiales;f__Lachnospiraceae
38415	9.89266547	0.00710943	0.07089587	12.2	60.8	15.8	k__Bacteria;p__Firmicutes;c__Clostridia;o__Clostridiales;f__Lachnospiraceae
264345	9.66924829	0.00794968	0.07089587	14	76.6	0	k__Bacteria;p__Firmicutes;c__Clostridia;o__Clostridiales;f__Lachnospiraceae
4393892	9.6372093	0.00807805	0.07089587	153.8	40.2	49.2	k__Bacteria;p__Firmicutes;c__Clostridia;o__Clostridiales;f__Lachnospiraceae
New.ReferenceOTU185	8.80145191	0.01226843	0.0773068	6	1	5.4	k__Bacteria;p__Firmicutes;c__Clostridia;o__Clostridiales;f__Lachnospiraceae
1991255	8.65501859	0.01320038	0.07917455	3.4	0.2	3	k__Bacteria;p__Firmicutes;c__Clostridia;o__Clostridiales;f__Lachnospiraceae
190425	8.4	0.01499558	0.08141434	5.6	3.6	1	k__Bacteria;p__Firmicutes;c__Clostridia;o__Clostridiales;f__Lachnospiraceae
747987	8.30075949	0.01575843	0.08141434	0	0.2	7.2	k__Bacteria;p__Firmicutes;c__Clostridia;o__Clostridiales;f__Lachnospiraceae
4418586	8.30075949	0.01575843	0.08141434	0	0.2	18.6	k__Bacteria;p__Firmicutes;c__Clostridia;o__Clostridiales;f__Lachnospiraceae
277497	8.02599278	0.01807914	0.08736524	1.6	12.8	8.6	k__Bacteria;p__Firmicutes;c__Clostridia;o__Clostridiales;f__Lachnospiraceae
180800	10.0914498	0.00643679	0.07089587	13.6	0.6	77.2	k__Bacteria;p__Firmicutes;c__Clostridia;o__Clostridiales;f__Lachnospiraceae;g__;s__
193680	9.71234347	0.00778021	0.07089587	30	18.4	2.8	k__Bacteria;p__Firmicutes;c__Clostridia;o__Clostridiales;f__Lachnospiraceae;g__;s__
258522	9.58054054	0.00831021	0.07089587	74.2	2.6	210.6	k__Bacteria;p__Firmicutes;c__Clostridia;o__Clostridiales;f__Lachnospiraceae;g__;s__
172161	9.38823529	0.00914894	0.07089587	1.6	0.2	21.4	k__Bacteria;p__Firmicutes;c__Clostridia;o__Clostridiales;f__Lachnospiraceae;g__;s__
266192	9.38	0.00918669	0.07089587	37	96.8	55.8	k__Bacteria;p__Firmicutes;c__Clostridia;o__Clostridiales;f__Lachnospiraceae;g__;s__
180105	8.93454545	0.01147858	0.0759541	3.2	0	19.4	k__Bacteria;p__Firmicutes;c__Clostridia;o__Clostridiales;f__Lachnospiraceae;g__;s__
273091	8.61374046	0.01347566	0.07936454	5.4	0.6	40.2	k__Bacteria;p__Firmicutes;c__Clostridia;o__Clostridiales;f__Lachnospiraceae;g__;s__
327739	8.30075949	0.01575843	0.08141434	8	0	0.2	k__Bacteria;p__Firmicutes;c__Clostridia;o__Clostridiales;f__Lachnospiraceae;g__;s__
188078	8.30075949	0.01575843	0.08141434	34.4	0.2	0	k__Bacteria;p__Firmicutes;c__Clostridia;o__Clostridiales;f__Lachnospiraceae;g__;s__
186052	8.26953405	0.01600639	0.08141434	22.4	11.6	7.4	k__Bacteria;p__Firmicutes;c__Clostridia;o__Clostridiales;f__Lachnospiraceae;g__;s__
261114	8.18778947	0.01667417	0.08342869	1.4	0.4	17.2	k__Bacteria;p__Firmicutes;c__Clostridia;o__Clostridiales;f__Lachnospiraceae;g__;s__
261178	8.02222222	0.01811326	0.08736524	17.8	0.2	60.8	k__Bacteria;p__Firmicutes;c__Clostridia;o__Clostridiales;f__Lachnospiraceae;g__;s__
346648	7.54671	0.02297	0.09791	12	7	0.8	k__Bacteria;p__Firmicutes;c__Clostridia;o__Clostridiales;f__Lachnospiraceae;g__;s__

	533	479	994				
3750373	12.5899281	0.00184558	0.07089587	2.6	31.6	9	k__Bacteria;p__Firmicutes;c__Clostridia;o__Clostridiales;f__Lachnospiraceae;g__[Ruminococcus];s__gnavus
262537	10.6520646	0.00486333	0.07089587	5.2	25.2	14.8	k__Bacteria;p__Firmicutes;c__Clostridia;o__Clostridiales;f__Lachnospiraceae;g__[Ruminococcus];s__gnavus
2897332	9.15109489	0.01030066	0.07141835	8.6	1.8	6.2	k__Bacteria;p__Firmicutes;c__Clostridia;o__Clostridiales;f__Lachnospiraceae;g__[Ruminococcus];s__gnavus
266726	12.0909091	0.0023686	0.07089587	0.2	0	10	k__Bacteria;p__Firmicutes;c__Clostridia;o__Clostridiales;f__Lachnospiraceae;g__Coprococcus
182016	11.3284404	0.00346785	0.07089587	0.2	0	4.6	k__Bacteria;p__Firmicutes;c__Clostridia;o__Clostridiales;f__Lachnospiraceae;g__Coprococcus;s__
262227	8.32182741	0.0155933	0.08141434	0.4	0	6.6	k__Bacteria;p__Firmicutes;c__Clostridia;o__Clostridiales;f__Lachnospiraceae;g__Coprococcus;s__
309480	10.0157706	0.00668503	0.07089587	6.2	39.8	8.2	k__Bacteria;p__Firmicutes;c__Clostridia;o__Clostridiales;f__Lachnospiraceae;g__Dorea
192204	9.06	0.01078068	0.07355839	23.8	108	36.6	k__Bacteria;p__Firmicutes;c__Clostridia;o__Clostridiales;f__Lachnospiraceae;g__Dorea
193509	8.70677291	0.01286318	0.07848116	3.2	0	7.6	k__Bacteria;p__Firmicutes;c__Clostridia;o__Clostridiales;f__Lachnospiraceae;g__Dorea
170185	9.91150442	0.00704278	0.07089587	2.2	0	0	k__Bacteria;p__Firmicutes;c__Clostridia;o__Clostridiales;f__Ruminococcaceae
389529	9.66242775	0.00797683	0.07089587	3.8	0	1.6	k__Bacteria;p__Firmicutes;c__Clostridia;o__Clostridiales;f__Ruminococcaceae
199307	9.64444444	0.00804888	0.07089587	11.8	0.2	7	k__Bacteria;p__Firmicutes;c__Clostridia;o__Clostridiales;f__Ruminococcaceae
344906	9.58540146	0.00829004	0.07089587	10	0.2	9.4	k__Bacteria;p__Firmicutes;c__Clostridia;o__Clostridiales;f__Ruminococcaceae
177271	8.4	0.01499558	0.08141434	8.4	0	7	k__Bacteria;p__Firmicutes;c__Clostridia;o__Clostridiales;f__Ruminococcaceae
273586	8.12954545	0.01716689	0.08431842	1.6	0.2	3.8	k__Bacteria;p__Firmicutes;c__Clostridia;o__Clostridiales;f__Ruminococcaceae
375106	8.128	0.01718016	0.08431842	4	0.2	10	k__Bacteria;p__Firmicutes;c__Clostridia;o__Clostridiales;f__Ruminococcaceae
New.ReferenceOTU617	7.63585657	0.02197328	0.0953027	3.4	0	3.8	k__Bacteria;p__Firmicutes;c__Clostridia;o__Clostridiales;f__Ruminococcaceae
192598	8.27272727	0.01598086	0.08141434	8.2	0	13	k__Bacteria;p__Firmicutes;c__Clostridia;o__Clostridiales;f__Ruminococcaceae;g__;s__
180879	12.6126126	0.00182476	0.07089587	34	1.4	13.8	k__Bacteria;p__Firmicutes;c__Clostridia;o__Clostridiales;f__Ruminococcaceae;g__Oscillospira;s__
356055	11.7131474	0.00286103	0.07089587	1	0	5.4	k__Bacteria;p__Firmicutes;c__Clostridia;o__Clostridiales;f__Ruminococcaceae;g__Oscillospira;s__
166226	11.6783599	0.00291123	0.07089587	0.2	14.8	0	k__Bacteria;p__Firmicutes;c__Clostridia;o__Clostridiales;f__Ruminococcaceae;g__Oscillospira;s__
267689	11.0344086	0.00401706	0.07089587	21.8	5.2	86.6	k__Bacteria;p__Firmicutes;c__Clostridia;o__Clostridiales;f__Ruminococcaceae;g__Oscillospira;s__
178959	10.8978417	0.00430094	0.07089587	24.6	2	52	k__Bacteria;p__Firmicutes;c__Clostridia;o__Clostridiales;f__Ruminococcaceae;g__Oscillospira;s__
New.ReferenceOTU9	10.8219178	0.00446735	0.07089587	0.6	0.2	3	k__Bacteria;p__Firmicutes;c__Clostridia;o__Clostridiales;f__Ruminococcaceae;g__Oscillospira;s__

307608	10.4222222	0.00545561	0.07089587	1.8	13.4	31.6	k__Bacteria;p__Firmicutes;c__Clostridia;o__Clostridiales;f__Ruminococcaceae;g__Oscillospira;s__
1110253	10.372844	0.00559198	0.07089587	2.2	0.6	15.6	k__Bacteria;p__Firmicutes;c__Clostridia;o__Clostridiales;f__Ruminococcaceae;g__Oscillospira;s__
265793	9.89184891	0.00711234	0.07089587	1	0.2	10	k__Bacteria;p__Firmicutes;c__Clostridia;o__Clostridiales;f__Ruminococcaceae;g__Oscillospira;s__
275423	9.6797075	0.00790821	0.07089587	2.2	1	115	k__Bacteria;p__Firmicutes;c__Clostridia;o__Clostridiales;f__Ruminococcaceae;g__Oscillospira;s__
167034	9.5059246	0.0086261	0.07089587	22.6	5	27.6	k__Bacteria;p__Firmicutes;c__Clostridia;o__Clostridiales;f__Ruminococcaceae;g__Oscillospira;s__
332608	9.4190991	0.00900883	0.07089587	1	7.6	5	k__Bacteria;p__Firmicutes;c__Clostridia;o__Clostridiales;f__Ruminococcaceae;g__Oscillospira;s__
180235	9.23116883	0.0098964	0.07133005	2	12.4	0.8	k__Bacteria;p__Firmicutes;c__Clostridia;o__Clostridiales;f__Ruminococcaceae;g__Oscillospira;s__
165046	9.14936709	0.01030956	0.07141835	17.6	0.8	32.6	k__Bacteria;p__Firmicutes;c__Clostridia;o__Clostridiales;f__Ruminococcaceae;g__Oscillospira;s__
191558	8.80218182	0.01226395	0.0773068	9.8	9	17.4	k__Bacteria;p__Firmicutes;c__Clostridia;o__Clostridiales;f__Ruminococcaceae;g__Oscillospira;s__
273648	8.72288288	0.01275998	0.07847564	13.2	0.8	15.4	k__Bacteria;p__Firmicutes;c__Clostridia;o__Clostridiales;f__Ruminococcaceae;g__Oscillospira;s__
180535	8.48220183	0.01439174	0.08141434	2.2	0.6	9.2	k__Bacteria;p__Firmicutes;c__Clostridia;o__Clostridiales;f__Ruminococcaceae;g__Oscillospira;s__
832558	8.20726577	0.01651258	0.08323232	4.2	0.4	14.6	k__Bacteria;p__Firmicutes;c__Clostridia;o__Clostridiales;f__Ruminococcaceae;g__Oscillospira;s__
275543	7.99922631	0.01832273	0.08756381	1.2	0.4	5.6	k__Bacteria;p__Firmicutes;c__Clostridia;o__Clostridiales;f__Ruminococcaceae;g__Oscillospira;s__
304080	7.80436364	0.0201978	0.09225034	5.6	1.2	10.6	k__Bacteria;p__Firmicutes;c__Clostridia;o__Clostridiales;f__Ruminococcaceae;g__Oscillospira;s__
258725	7.70133333	0.02126555	0.09405913	14.8	0.2	36	k__Bacteria;p__Firmicutes;c__Clostridia;o__Clostridiales;f__Ruminococcaceae;g__Oscillospira;s__
189840	7.59355993	0.02244292	0.09696382	21.4	26.8	170	k__Bacteria;p__Firmicutes;c__Clostridia;o__Clostridiales;f__Ruminococcaceae;g__Oscillospira;s__
194662	7.55443038	0.02288634	0.09791994	6.6	2.2	13.4	k__Bacteria;p__Firmicutes;c__Clostridia;o__Clostridiales;f__Ruminococcaceae;g__Oscillospira;s__
261365	10.2788104	0.00586117	0.07089587	5.2	0	10.8	k__Bacteria;p__Firmicutes;c__Clostridia;o__Clostridiales;f__Ruminococcaceae;g__Ruminococcus;s__
268720	9.38	0.00918669	0.07089587	31.2	44.4	14.4	k__Bacteria;p__Firmicutes;c__Clostridia;o__Clostridiales;f__Ruminococcaceae;g__Ruminococcus;s__
189147	8.82960289	0.01209696	0.0773068	19	0.6	20	k__Bacteria;p__Firmicutes;c__Clostridia;o__Clostridiales;f__Ruminococcaceae;g__Ruminococcus;s__

*Graphically represented in Supplementary Figure 2e

Supplementary Table 6. Biology H2 Congenics (Figure 2i-l) Significant differences by OTU*

OTU	Test-Statistic	P	FDR_P	BB_mean	DD_mean	KK_mean	taxonomy
EF097218	8.97795591	0.01123212	0.08788206	1.8	0	3.8	Bacteria; __Actinobacteria; __Coriobacteriia; __Coriobacteriales; __Coriobacteriaceae; __Enterorhabdus
DQ014947	13.2911392	0.00129977	0.04406832	22	0	0	Bacteria; __Bacteroidetes; __Bacteroidia; __Bacteroidales; __Porphyromonadaceae; __Barnesiella
EF614623	12.962963	0.00153154	0.0464024	274.2	0	698.2	Bacteria; __Bacteroidetes; __Bacteroidia; __Bacteroidales; __Prevotellaceae; __g
EF096895	11.5207767	0.00314989	0.06579326	1.4	0	5.4	Bacteria; __Bacteroidetes; __Bacteroidia; __Bacteroidales; __Prevotellaceae; __g

EU505099	13.0841121	0.00144152	0.0464024	5	0	14.4	Bacteria; __Bacteroidetes; __Bacteroidia; __Bacteroidales; __Prevotellaceae; __Prevotella
DQ815668	13.3248731	0.00127803	0.04406832	5.2	0	0	Bacteria; __Bacteroidetes; __Bacteroidia; __Bacteroidales; __Rikenellaceae; __Alistipes
AY991070	11.6176471	0.00300096	0.06579326	168	0	18.2	Bacteria; __Bacteroidetes; __Bacteroidia; __Bacteroidales; __Rikenellaceae; __Alistipes
EU457823	11.3544828	0.00342299	0.06579326	2.8	0	0.2	Bacteria; __Bacteroidetes; __Bacteroidia; __Bacteroidales; __Rikenellaceae; __Alistipes
EU655844	9.91150442	0.00704278	0.06579326	3.4	0	0	Bacteria; __Bacteroidetes; __Bacteroidia; __Bacteroidales; __Rikenellaceae; __Alistipes
EF100034	8.82	0.01215518	0.09266714	16.8	79	70	Bacteria; __Bacteroidetes; __Bacteroidia; __Bacteroidales; __Rikenellaceae; __Alistipes
EF406867	13.42711	0.00121434	0.04406832	2.4	0	0	Bacteria; __Bacteroidetes; __Bacteroidia; __Bacteroidales; __S24-7; __g
EU505154	13.3248731	0.00127803	0.04406832	36.8	0	0	Bacteria; __Bacteroidetes; __Bacteroidia; __Bacteroidales; __S24-7; __g
EF097510	13.3248731	0.00127803	0.04406832	4.8	0	0	Bacteria; __Bacteroidetes; __Bacteroidia; __Bacteroidales; __S24-7; __g
EU505465	13.3248731	0.00127803	0.04406832	7.2	0	0	Bacteria; __Bacteroidetes; __Bacteroidia; __Bacteroidales; __S24-7; __g
EF406803	13.3248731	0.00127803	0.04406832	11.8	0	0	Bacteria; __Bacteroidetes; __Bacteroidia; __Bacteroidales; __S24-7; __g
EF098040	13.2911392	0.00129977	0.04406832	6	0	0	Bacteria; __Bacteroidetes; __Bacteroidia; __Bacteroidales; __S24-7; __g
EU504810	13.2911392	0.00129977	0.04406832	19.2	0	0	Bacteria; __Bacteroidetes; __Bacteroidia; __Bacteroidales; __S24-7; __g
EU791170	13.2911392	0.00129977	0.04406832	56.4	0	0	Bacteria; __Bacteroidetes; __Bacteroidia; __Bacteroidales; __S24-7; __g
EU456778	13.2911392	0.00129977	0.04406832	73	0	0	Bacteria; __Bacteroidetes; __Bacteroidia; __Bacteroidales; __S24-7; __g
EU504432	13.2911392	0.00129977	0.04406832	30.8	0	0	Bacteria; __Bacteroidetes; __Bacteroidia; __Bacteroidales; __S24-7; __g
EF097526	13.2911392	0.00129977	0.04406832	10.6	0	0	Bacteria; __Bacteroidetes; __Bacteroidia; __Bacteroidales; __S24-7; __g
EU655810	13.2911392	0.00129977	0.04406832	56.4	0	0	Bacteria; __Bacteroidetes; __Bacteroidia; __Bacteroidales; __S24-7; __g
EF099648	13.2911392	0.00129977	0.04406832	13.2	0	0	Bacteria; __Bacteroidetes; __Bacteroidia; __Bacteroidales; __S24-7; __g
EF097554	13.2911392	0.00129977	0.04406832	6.6	0	0	Bacteria; __Bacteroidetes; __Bacteroidia; __Bacteroidales; __S24-7; __g
EF406486	13.2911392	0.00129977	0.04406832	56.6	0	0	Bacteria; __Bacteroidetes; __Bacteroidia; __Bacteroidales; __S24-7; __g
EU510749	13.0841121	0.00144152	0.0464024	12.2	0	27	Bacteria; __Bacteroidetes; __Bacteroidia; __Bacteroidales; __S24-7; __g
EU453830	12.987013	0.00151323	0.0464024	5.4	0	25.6	Bacteria; __Bacteroidetes; __Bacteroidia; __Bacteroidales; __S24-7; __g
EU457245	12.962963	0.00153154	0.0464024	7	0	48.8	Bacteria; __Bacteroidetes; __Bacteroidia; __Bacteroidales; __S24-7; __g
EU452656	12.1797323	0.00226571	0.06579326	2	0	22.4	Bacteria; __Bacteroidetes; __Bacteroidia; __Bacteroidales; __S24-7;

							__g
EF406569	12.0909091	0.0023686	0.06579326	13.4	0.2	0	Bacteria; __Bacteroidetes; __Bacteroidia; __Bacteroidales; __S24-7; __g
EU510625	11.6898608	0.00289454	0.06579326	11.8	0	75.4	Bacteria; __Bacteroidetes; __Bacteroidia; __Bacteroidales; __S24-7; __g
AY990698	11.6783599	0.00291123	0.06579326	0.2	0	3.6	Bacteria; __Bacteroidetes; __Bacteroidia; __Bacteroidales; __S24-7; __g
EU504803	10.9686441	0.00415135	0.06579326	5.8	0	1.4	Bacteria; __Bacteroidetes; __Bacteroidia; __Bacteroidales; __S24-7; __g
EU503894	10.6181818	0.00494642	0.06579326	4.6	0	8.4	Bacteria; __Bacteroidetes; __Bacteroidia; __Bacteroidales; __S24-7; __g
EU504801	10.5291089	0.0051717	0.06579326	1	0	2.8	Bacteria; __Bacteroidetes; __Bacteroidia; __Bacteroidales; __S24-7; __g
EU504568	10.4463551	0.00539017	0.06579326	6.8	0	4.8	Bacteria; __Bacteroidetes; __Bacteroidia; __Bacteroidales; __S24-7; __g
EU504184	10.3413333	0.00568078	0.06579326	2.4	0	1.8	Bacteria; __Bacteroidetes; __Bacteroidia; __Bacteroidales; __S24-7; __g
EU505382	10.1818182	0.00615242	0.06579326	0.8	0	0	Bacteria; __Bacteroidetes; __Bacteroidia; __Bacteroidales; __S24-7; __g
EU452809	10	0.00673795	0.06579326	1.2	0	0	Bacteria; __Bacteroidetes; __Bacteroidia; __Bacteroidales; __S24-7; __g
EF097783	10	0.00673795	0.06579326	1	0	0	Bacteria; __Bacteroidetes; __Bacteroidia; __Bacteroidales; __S24-7; __g
EF096871	9.9408284	0.00694027	0.06579326	1.2	0	0	Bacteria; __Bacteroidetes; __Bacteroidia; __Bacteroidales; __S24-7; __g
EF406765	9.9408284	0.00694027	0.06579326	1.2	0	0	Bacteria; __Bacteroidetes; __Bacteroidia; __Bacteroidales; __S24-7; __g
EU457101	9.91150442	0.00704278	0.06579326	13.8	0	0	Bacteria; __Bacteroidetes; __Bacteroidia; __Bacteroidales; __S24-7; __g
EF406873	9.91150442	0.00704278	0.06579326	3.4	0	0	Bacteria; __Bacteroidetes; __Bacteroidia; __Bacteroidales; __S24-7; __g
EF406646	9.91150442	0.00704278	0.06579326	1.6	0	0	Bacteria; __Bacteroidetes; __Bacteroidia; __Bacteroidales; __S24-7; __g
EF096622	9.91150442	0.00704278	0.06579326	1.8	0	0	Bacteria; __Bacteroidetes; __Bacteroidia; __Bacteroidales; __S24-7; __g
EF614662	9.91150442	0.00704278	0.06579326	5.6	0	0	Bacteria; __Bacteroidetes; __Bacteroidia; __Bacteroidales; __S24-7; __g
EF406710	9.91150442	0.00704278	0.06579326	1.4	0	0	Bacteria; __Bacteroidetes; __Bacteroidia; __Bacteroidales; __S24-7; __g
EF406592	9.88235294	0.00714619	0.06579326	18.2	0	0	Bacteria; __Bacteroidetes; __Bacteroidia; __Bacteroidales; __S24-7; __g
EU503917	9.88235294	0.00714619	0.06579326	0	8.4	0	Bacteria; __Bacteroidetes; __Bacteroidia; __Bacteroidales; __S24-7; __g
EF406815	9.88235294	0.00714619	0.06579326	2	0	0	Bacteria; __Bacteroidetes; __Bacteroidia; __Bacteroidales; __S24-7; __g
EF098484	9.85243446	0.00725389	0.06579326	4.8	0	6	Bacteria; __Bacteroidetes; __Bacteroidia; __Bacteroidales; __S24-7; __g
EF099832	9.83770197	0.00730752	0.0658602	8.4	70.4	4	Bacteria; __Bacteroidetes; __Bacteroidia; __Bacteroidales; __S24-7; __g

EU455919	9.79490909	0.00746556	0.06670634	117.8	11.6	105	Bacteria; __Bacteroidetes; __Bacteroidia; __Bacteroidales; __S24-7; __g
EF099130	9.75742397	0.00760681	0.06686476	19.6	48.4	81.6	Bacteria; __Bacteroidetes; __Bacteroidia; __Bacteroidales; __S24-7; __g
EU504899	9.72740741	0.00772183	0.0670481	423.2	0	383.6	Bacteria; __Bacteroidetes; __Bacteroidia; __Bacteroidales; __S24-7; __g
EU455595	9.59127273	0.00826574	0.07018886	234.8	26	206.4	Bacteria; __Bacteroidetes; __Bacteroidia; __Bacteroidales; __S24-7; __g
EF406607	9.3996357	0.00909693	0.07575458	25.8	2.2	23.2	Bacteria; __Bacteroidetes; __Bacteroidia; __Bacteroidales; __S24-7; __g
EU455460	9.32008114	0.00946608	0.07791732	2.8	0	1	Bacteria; __Bacteroidetes; __Bacteroidia; __Bacteroidales; __S24-7; __g
EU458112	9.30569476	0.00953442	0.07801507	15	4.4	0	Bacteria; __Bacteroidetes; __Bacteroidia; __Bacteroidales; __S24-7; __g
EU507474	9.2765653	0.0096743	0.07815183	20.6	603.2	50.8	Bacteria; __Bacteroidetes; __Bacteroidia; __Bacteroidales; __S24-7; __g
EF406866	9.26835443	0.0097141	0.07815183	3.6	1	0	Bacteria; __Bacteroidetes; __Bacteroidia; __Bacteroidales; __S24-7; __g
EU504185	9.04373832	0.01086869	0.08598341	0.8	11.6	0.8	Bacteria; __Bacteroidetes; __Bacteroidia; __Bacteroidales; __S24-7; __g
EF099162	8.60400729	0.0135414	0.09991167	27.6	3.4	21	Bacteria; __Bacteroidetes; __Bacteroidia; __Bacteroidales; __S24-7; __g
EU509932	12.0909091	0.0023686	0.06579326	14	0	0.6	Bacteria; __Bacteroidetes; __VC2.1_Bac22; __o; __f; __g
DQ815839	13.2911392	0.00129977	0.04406832	0	0	56.8	Bacteria; __Candidate_division_TM7; __c; __o; __f; __g
HM124025	13.2911392	0.00129977	0.04406832	0	0	18.4	Bacteria; __Candidate_division_TM7; __c; __o; __f; __g
EF095966	13.2911392	0.00129977	0.04406832	0	0	23.8	Bacteria; __Candidate_division_TM7; __c; __o; __f; __g
AY991178	9.88235294	0.00714619	0.06579326	0	0	19.8	Bacteria; __Candidate_division_TM7; __c; __o; __f; __g
EU510641	13.3587786	0.00125655	0.04406832	0	0	6.2	Bacteria; __Firmicutes; __Bacilli; __Lactobacillales; __Lactobacillaceae; __Lactobacillus
EF096152	11.6898608	0.00289454	0.06579326	1	23	0	Bacteria; __Firmicutes; __Bacilli; __Lactobacillales; __Lactobacillaceae; __Lactobacillus
EU505198	10.6975701	0.00475392	0.06579326	4.4	0	10.8	Bacteria; __Firmicutes; __Bacilli; __Lactobacillales; __Lactobacillaceae; __Lactobacillus
EU505448	10.3496296	0.00565726	0.06579326	28.8	0	45.6	Bacteria; __Firmicutes; __Bacilli; __Lactobacillales; __Lactobacillaceae; __Lactobacillus
EU457074	9.88235294	0.00714619	0.06579326	0	142.8	0	Bacteria; __Firmicutes; __Bacilli; __Lactobacillales; __Lactobacillaceae; __Lactobacillus
EU451393	9.78701299	0.00749509	0.06670634	10.2	0	12.4	Bacteria; __Firmicutes; __Bacilli; __Lactobacillales; __Lactobacillaceae; __Lactobacillus
EU451013	9.76888889	0.00756332	0.06686476	26.4	0	34	Bacteria; __Firmicutes; __Bacilli; __Lactobacillales; __Lactobacillaceae; __Lactobacillus
EU761885	9.91150442	0.00704278	0.06579326	2.4	0	0	Bacteria; __Firmicutes; __Clostridia; __Clostridiales; __Christensenellaceae; __Christensenella
EU469268	13.6363636	0.00109371	0.04406832	1.2	0	0	Bacteria; __Firmicutes; __Clostridia; __Clostridiales; __Christensenellaceae; __g
EF099030	10	0.00673795	0.06579326	0	1.4	0	Bacteria; __Firmicutes; __Clostridia; __Clostridiales; __Family_XIII_Incertae_Sedis; __Incertae_Sedis

AY993664	9.91150442	0.00704278	0.06579326	0	0	1.8	Bacteria; __Firmicutes; __Clostridia; __Clostridiales; __Lachnospiraceae; __Coprococcus
EU511743	9.88235294	0.00714619	0.06579326	49.6	0	0	Bacteria; __Firmicutes; __Clostridia; __Clostridiales; __Lachnospiraceae; __Coprococcus
EU462089	10.1818182	0.00615242	0.06579326	0	0	0.8	Bacteria; __Firmicutes; __Clostridia; __Clostridiales; __Lachnospiraceae; __Dorea
EU456889	13.42711	0.00121434	0.04406832	0	2.8	0	Bacteria; __Firmicutes; __Clostridia; __Clostridiales; __Lachnospiraceae; __g
EU503956	13.42711	0.00121434	0.04406832	1.6	0	0	Bacteria; __Firmicutes; __Clostridia; __Clostridiales; __Lachnospiraceae; __g
EU457789	13.3248731	0.00127803	0.04406832	0	0	2.4	Bacteria; __Firmicutes; __Clostridia; __Clostridiales; __Lachnospiraceae; __g
DQ015528	13.3248731	0.00127803	0.04406832	0	2.8	0	Bacteria; __Firmicutes; __Clostridia; __Clostridiales; __Lachnospiraceae; __g
EU504205	13.2911392	0.00129977	0.04406832	44.8	0	0	Bacteria; __Firmicutes; __Clostridia; __Clostridiales; __Lachnospiraceae; __g
EU509990	13.2911392	0.00129977	0.04406832	6.2	0	0	Bacteria; __Firmicutes; __Clostridia; __Clostridiales; __Lachnospiraceae; __g
EU503953	13.2911392	0.00129977	0.04406832	47.4	0	0	Bacteria; __Firmicutes; __Clostridia; __Clostridiales; __Lachnospiraceae; __g
EU503995	13.2911392	0.00129977	0.04406832	63	0	0	Bacteria; __Firmicutes; __Clostridia; __Clostridiales; __Lachnospiraceae; __g
EU503881	13.2911392	0.00129977	0.04406832	26.6	0	0	Bacteria; __Firmicutes; __Clostridia; __Clostridiales; __Lachnospiraceae; __g
EU504214	13.2911392	0.00129977	0.04406832	6.6	0	0	Bacteria; __Firmicutes; __Clostridia; __Clostridiales; __Lachnospiraceae; __g
EU508368	12.0909091	0.0023686	0.06579326	1	42.2	0	Bacteria; __Firmicutes; __Clostridia; __Clostridiales; __Lachnospiraceae; __g
EU508707	11.9592308	0.0025298	0.06579326	1	9.2	0	Bacteria; __Firmicutes; __Clostridia; __Clostridiales; __Lachnospiraceae; __g
EU510102	11.7684211	0.00278304	0.06579326	3	10.2	0	Bacteria; __Firmicutes; __Clostridia; __Clostridiales; __Lachnospiraceae; __g
EU453073	11.3544828	0.00342299	0.06579326	0	3.4	0.4	Bacteria; __Firmicutes; __Clostridia; __Clostridiales; __Lachnospiraceae; __g
EU504233	11.3284404	0.00346785	0.06579326	0	0.2	4	Bacteria; __Firmicutes; __Clostridia; __Clostridiales; __Lachnospiraceae; __g
EU510378	11.0907063	0.00390556	0.06579326	4	0	15.2	Bacteria; __Firmicutes; __Clostridia; __Clostridiales; __Lachnospiraceae; __g
EU511776	11.0453333	0.00399518	0.06579326	3.8	0	13.4	Bacteria; __Firmicutes; __Clostridia; __Clostridiales; __Lachnospiraceae; __g
EU511760	10.9686441	0.00415135	0.06579326	16.8	0	0.4	Bacteria; __Firmicutes; __Clostridia; __Clostridiales; __Lachnospiraceae; __g
EU509071	10.9557809	0.00417813	0.06579326	1	0	15	Bacteria; __Firmicutes; __Clostridia; __Clostridiales; __Lachnospiraceae; __g
EU455386	10.8587814	0.00438577	0.06579326	1.6	8.6	18.8	Bacteria; __Firmicutes; __Clostridia; __Clostridiales; __Lachnospiraceae; __g
EU454243	10.7476923	0.00463627	0.06579326	0.2	0.8	28	Bacteria; __Firmicutes; __Clostridia; __Clostridiales; __Lachnospiraceae; __g
EU455488	10.6669231	0.00482733	0.06579326	1.2	8.4	0	Bacteria; __Firmicutes; __Clostridia; __Clostridiales;

							__Lachnospiraceae; __g
EU511991	10.5617647	0.00508794	0.06579326	0.4	2.2	5.2	Bacteria; __Firmicutes; __Clostridia; __Clostridiales; __Lachnospiraceae; __g
AY990911	10.5056	0.00523285	0.06579326	4.4	0	0.8	Bacteria; __Firmicutes; __Clostridia; __Clostridiales; __Lachnospiraceae; __g
EF099230	10.3610687	0.005625	0.06579326	2.8	21.2	0	Bacteria; __Firmicutes; __Clostridia; __Clostridiales; __Lachnospiraceae; __g
EU505206	10.240481	0.00597459	0.06579326	0.8	5.2	0	Bacteria; __Firmicutes; __Clostridia; __Clostridiales; __Lachnospiraceae; __g
EU457662	10.1818182	0.00615242	0.06579326	0	0.8	0	Bacteria; __Firmicutes; __Clostridia; __Clostridiales; __Lachnospiraceae; __g
EU504592	10.1066667	0.006388	0.06579326	0.4	0	1.8	Bacteria; __Firmicutes; __Clostridia; __Clostridiales; __Lachnospiraceae; __g
AM932651	10.0912409	0.00643747	0.06579326	3	1.6	43.6	Bacteria; __Firmicutes; __Clostridia; __Clostridiales; __Lachnospiraceae; __g
EU504066	10.0671756	0.00651539	0.06579326	0.6	1.4	33.2	Bacteria; __Firmicutes; __Clostridia; __Clostridiales; __Lachnospiraceae; __g
EU451636	10.0507463	0.00656913	0.06579326	4.4	0	5.6	Bacteria; __Firmicutes; __Clostridia; __Clostridiales; __Lachnospiraceae; __g
EU457126	10.0258065	0.00665156	0.06579326	0	1	4	Bacteria; __Firmicutes; __Clostridia; __Clostridiales; __Lachnospiraceae; __g
EU503625	10	0.00673795	0.06579326	1.2	0	0	Bacteria; __Firmicutes; __Clostridia; __Clostridiales; __Lachnospiraceae; __g
EU454538	9.9408284	0.00694027	0.06579326	0	1.6	0	Bacteria; __Firmicutes; __Clostridia; __Clostridiales; __Lachnospiraceae; __g
EU452997	9.91150442	0.00704278	0.06579326	0	1.6	0	Bacteria; __Firmicutes; __Clostridia; __Clostridiales; __Lachnospiraceae; __g
EU504224	9.91150442	0.00704278	0.06579326	1.4	0	0	Bacteria; __Firmicutes; __Clostridia; __Clostridiales; __Lachnospiraceae; __g
GQ493732	9.91150442	0.00704278	0.06579326	0	0	1.6	Bacteria; __Firmicutes; __Clostridia; __Clostridiales; __Lachnospiraceae; __g
AY992736	9.91150442	0.00704278	0.06579326	1.8	0	0	Bacteria; __Firmicutes; __Clostridia; __Clostridiales; __Lachnospiraceae; __g
EU503678	9.9059761	0.00706227	0.06579326	0	4	1.2	Bacteria; __Firmicutes; __Clostridia; __Clostridiales; __Lachnospiraceae; __g
EU505155	9.88235294	0.00714619	0.06579326	0	0	59.4	Bacteria; __Firmicutes; __Clostridia; __Clostridiales; __Lachnospiraceae; __g
EF098033	9.88235294	0.00714619	0.06579326	0	2.4	0	Bacteria; __Firmicutes; __Clostridia; __Clostridiales; __Lachnospiraceae; __g
EU504017	9.88235294	0.00714619	0.06579326	6.2	0	0	Bacteria; __Firmicutes; __Clostridia; __Clostridiales; __Lachnospiraceae; __g
EU507860	9.88235294	0.00714619	0.06579326	8.6	0	0	Bacteria; __Firmicutes; __Clostridia; __Clostridiales; __Lachnospiraceae; __g
EU503948	9.88235294	0.00714619	0.06579326	2.4	0	0	Bacteria; __Firmicutes; __Clostridia; __Clostridiales; __Lachnospiraceae; __g
EU457269	9.87368421	0.00717723	0.06579326	0	6.8	20	Bacteria; __Firmicutes; __Clostridia; __Clostridiales; __Lachnospiraceae; __g
EF096560	9.85263158	0.00725318	0.06579326	1.4	0.6	5	Bacteria; __Firmicutes; __Clostridia; __Clostridiales; __Lachnospiraceae; __g

EU509827	9.73284133	0.00770088	0.0670481	0.8	1	6.4	Bacteria; __Firmicutes; __Clostridia; __Clostridiales; __Lachnospiraceae; __g
EU507528	9.36266174	0.00926667	0.07671943	0.2	2.8	5.4	Bacteria; __Firmicutes; __Clostridia; __Clostridiales; __Lachnospiraceae; __g
EU504701	9.29458484	0.00958753	0.07801507	0.6	35.8	9	Bacteria; __Firmicutes; __Clostridia; __Clostridiales; __Lachnospiraceae; __g
EU505623	9.03208955	0.01093218	0.08600785	0.2	2.8	5.6	Bacteria; __Firmicutes; __Clostridia; __Clostridiales; __Lachnospiraceae; __g
EU511694	8.8648855	0.01188542	0.09198282	13.2	0.2	5.6	Bacteria; __Firmicutes; __Clostridia; __Clostridiales; __Lachnospiraceae; __g
EU510606	8.84756335	0.01198881	0.09228142	0.8	0.2	2.6	Bacteria; __Firmicutes; __Clostridia; __Clostridiales; __Lachnospiraceae; __g
EF098385	8.78843931	0.01234851	0.09303853	0.4	4.8	1	Bacteria; __Firmicutes; __Clostridia; __Clostridiales; __Lachnospiraceae; __g
EF100052	8.77028986	0.01246108	0.09339253	1.2	7.2	3.8	Bacteria; __Firmicutes; __Clostridia; __Clostridiales; __Lachnospiraceae; __g
EU503858	13.6363636	0.00109371	0.04406832	1.6	0	0	Bacteria; __Firmicutes; __Clostridia; __Clostridiales; __Lachnospiraceae; __Incertae_Sedis
EU504135	11.6666667	0.0029283	0.06579326	5.2	0	0.4	Bacteria; __Firmicutes; __Clostridia; __Clostridiales; __Lachnospiraceae; __Incertae_Sedis
EU454464	10	0.00673795	0.06579326	1	0	0	Bacteria; __Firmicutes; __Clostridia; __Clostridiales; __Lachnospiraceae; __Incertae_Sedis
AY989982	9.91150442	0.00704278	0.06579326	0	1.6	0	Bacteria; __Firmicutes; __Clostridia; __Clostridiales; __Lachnospiraceae; __Incertae_Sedis
EU510644	9.91150442	0.00704278	0.06579326	0	50.2	0	Bacteria; __Firmicutes; __Clostridia; __Clostridiales; __Lachnospiraceae; __Incertae_Sedis
EU510652	9.91150442	0.00704278	0.06579326	2.4	0	0	Bacteria; __Firmicutes; __Clostridia; __Clostridiales; __Lachnospiraceae; __Incertae_Sedis
EU451858	9.88235294	0.00714619	0.06579326	2.6	0	0	Bacteria; __Firmicutes; __Clostridia; __Clostridiales; __Lachnospiraceae; __Incertae_Sedis
EF098682	9.88235294	0.00714619	0.06579326	3.8	0	0	Bacteria; __Firmicutes; __Clostridia; __Clostridiales; __Lachnospiraceae; __Incertae_Sedis
EU512007	9.68634686	0.007882	0.06802406	1	1	9	Bacteria; __Firmicutes; __Clostridia; __Clostridiales; __Lachnospiraceae; __Incertae_Sedis
DQ808013	13.2911392	0.00129977	0.04406832	4.4	0	0	Bacteria; __Firmicutes; __Clostridia; __Clostridiales; __Lachnospiraceae; __Roseburia
EU504206	10	0.00673795	0.06579326	0	0	1	Bacteria; __Firmicutes; __Clostridia; __Clostridiales; __Lachnospiraceae; __Roseburia
DQ807348	9.91150442	0.00704278	0.06579326	2.4	0	0	Bacteria; __Firmicutes; __Clostridia; __Clostridiales; __Lachnospiraceae; __Roseburia
EU456198	9.5671028	0.00836623	0.07018886	0.2	2	3.2	Bacteria; __Firmicutes; __Clostridia; __Clostridiales; __Lachnospiraceae; __Roseburia
EU508322	13.2911392	0.00129977	0.04406832	146	0	0	Bacteria; __Firmicutes; __Clostridia; __Clostridiales; __Peptococcaceae; __g
EU507522	10.5456842	0.00512901	0.06579326	0.8	4.4	0	Bacteria; __Firmicutes; __Clostridia; __Clostridiales; __Ruminococcaceae; __Anaerotruncus
EU509296	9.57552743	0.00833107	0.07018886	0.8	11.2	0	Bacteria; __Firmicutes; __Clostridia; __Clostridiales; __Ruminococcaceae; __Anaerotruncus
AY993804	9.56398467	0.00837929	0.07018886	19	0.4	3.2	Bacteria; __Firmicutes; __Clostridia; __Clostridiales;

							__Ruminococcaceae; __Anaerotruncus
FJ880311	9.14520548	0.01033104	0.08233277	3.4	0.4	0.6	Bacteria; __Firmicutes; __Clostridia; __Clostridiales; __Ruminococcaceae; __Anaerotruncus
EU452161	8.61970803	0.01343551	0.09964671	1.6	19	7.6	Bacteria; __Firmicutes; __Clostridia; __Clostridiales; __Ruminococcaceae; __Anaerotruncus
EF614895	13.3587786	0.00125655	0.04406832	3.4	0	0	Bacteria; __Firmicutes; __Clostridia; __Clostridiales; __Ruminococcaceae; __g
EU504467	13.3587786	0.00125655	0.04406832	6	0	0	Bacteria; __Firmicutes; __Clostridia; __Clostridiales; __Ruminococcaceae; __g
EU506377	13.3248731	0.00127803	0.04406832	4	0	0	Bacteria; __Firmicutes; __Clostridia; __Clostridiales; __Ruminococcaceae; __g
EF098020	11.6176471	0.00300096	0.06579326	0	13.6	1.4	Bacteria; __Firmicutes; __Clostridia; __Clostridiales; __Ruminococcaceae; __g
EU511065	11.4333333	0.00329066	0.06579326	0.2	1.6	0	Bacteria; __Firmicutes; __Clostridia; __Clostridiales; __Ruminococcaceae; __g
EU504517	10.3496296	0.00565726	0.06579326	37.4	0	59.8	Bacteria; __Firmicutes; __Clostridia; __Clostridiales; __Ruminococcaceae; __g
EU507046	10.2253165	0.00602006	0.06579326	5	0	0.6	Bacteria; __Firmicutes; __Clostridia; __Clostridiales; __Ruminococcaceae; __g
EU510149	9.91150442	0.00704278	0.06579326	0	0	9	Bacteria; __Firmicutes; __Clostridia; __Clostridiales; __Ruminococcaceae; __g
AY992826	9.91150442	0.00704278	0.06579326	2.2	0	0	Bacteria; __Firmicutes; __Clostridia; __Clostridiales; __Ruminococcaceae; __g
EU508875	9.91150442	0.00704278	0.06579326	2.4	0	0	Bacteria; __Firmicutes; __Clostridia; __Clostridiales; __Ruminococcaceae; __g
EU510000	9.91150442	0.00704278	0.06579326	1.8	0	0	Bacteria; __Firmicutes; __Clostridia; __Clostridiales; __Ruminococcaceae; __g
DQ815745	9.88235294	0.00714619	0.06579326	0	11	0	Bacteria; __Firmicutes; __Clostridia; __Clostridiales; __Ruminococcaceae; __g
EU505209	9.88235294	0.00714619	0.06579326	0	2	0	Bacteria; __Firmicutes; __Clostridia; __Clostridiales; __Ruminococcaceae; __g
FJ681448	9.88235294	0.00714619	0.06579326	2.6	0	0	Bacteria; __Firmicutes; __Clostridia; __Clostridiales; __Ruminococcaceae; __g
EF097460	9.85996276	0.00722664	0.06579326	6	0	6.4	Bacteria; __Firmicutes; __Clostridia; __Clostridiales; __Ruminococcaceae; __g
FJ880035	9.65538462	0.00800497	0.06866917	1.8	0	0.6	Bacteria; __Firmicutes; __Clostridia; __Clostridiales; __Ruminococcaceae; __g
AY992012	8.95410526	0.01136687	0.08845037	1.2	14.6	0	Bacteria; __Firmicutes; __Clostridia; __Clostridiales; __Ruminococcaceae; __g
EU508855	8.79117647	0.01233162	0.09303853	9.4	14.6	0.4	Bacteria; __Firmicutes; __Clostridia; __Clostridiales; __Ruminococcaceae; __g
EU453498	13.6363636	0.00109371	0.04406832	0	1.4	0	Bacteria; __Firmicutes; __Clostridia; __Clostridiales; __Ruminococcaceae; __Incertae_Sedis
EF098406	13.2911392	0.00129977	0.04406832	0	41.4	0	Bacteria; __Firmicutes; __Clostridia; __Clostridiales; __Ruminococcaceae; __Incertae_Sedis
EU453982	11.6176471	0.00300096	0.06579326	0	59.8	1.4	Bacteria; __Firmicutes; __Clostridia; __Clostridiales; __Ruminococcaceae; __Incertae_Sedis
AM932668	10.9919321	0.00410329	0.06579326	2.6	0	0.4	Bacteria; __Firmicutes; __Clostridia; __Clostridiales; __Ruminococcaceae; __Incertae_Sedis

EF614814	10.9686441	0.00415135	0.06579326	0.4	4.8	0	Bacteria; __Firmicutes; __Clostridia; __Clostridiales; __Ruminococcaceae; __Incertae_Sedis
AY993110	10.0258065	0.00665156	0.06579326	0.8	0	3.6	Bacteria; __Firmicutes; __Clostridia; __Clostridiales; __Ruminococcaceae; __Incertae_Sedis
EF098043	10	0.00673795	0.06579326	0	1.2	0	Bacteria; __Firmicutes; __Clostridia; __Clostridiales; __Ruminococcaceae; __Incertae_Sedis
EU622687	10	0.00673795	0.06579326	1	0	0	Bacteria; __Firmicutes; __Clostridia; __Clostridiales; __Ruminococcaceae; __Incertae_Sedis
EU505069	9.88235294	0.00714619	0.06579326	0	2.6	0	Bacteria; __Firmicutes; __Clostridia; __Clostridiales; __Ruminococcaceae; __Incertae_Sedis
EU505157	8.81771562	0.01216907	0.09266714	0.6	0	1.4	Bacteria; __Firmicutes; __Clostridia; __Clostridiales; __Ruminococcaceae; __Incertae_Sedis
EU454374	13.2911392	0.00129977	0.04406832	0	0	7.8	Bacteria; __Firmicutes; __Clostridia; __Clostridiales; __Ruminococcaceae; __Oscillibacter
EU506730	11.6898608	0.00289454	0.06579326	1.8	0	23.2	Bacteria; __Firmicutes; __Clostridia; __Clostridiales; __uncultured; __g
GQ175414	9.91150442	0.00704278	0.06579326	2.8	0	0	Bacteria; __Firmicutes; __Clostridia; __Clostridiales; __uncultured; __g
EU505577	10.0727273	0.00649733	0.06579326	43	0	62.8	Bacteria; __Firmicutes; __Erysipelotrichi; __Erysipelotrichales; __Erysipelotrichaceae; __Allobaculum
EU510600	9.88235294	0.00714619	0.06579326	0	46.8	0	Bacteria; __Fusobacteria; __Fusobacteria; __SHA-35; __f; __g
AM932639	8.66046512	0.01316449	0.09814779	22.2	133.6	25.8	Bacteria; __Tenericutes; __Mollicutes; __Anaeroplasmatales; __Anaeroplasmataceae; __Anaeroplasma
EU457451	13.2911392	0.00129977	0.04406832	5.4	0	0	Bacteria; __Tenericutes; __Mollicutes; __RF9; __f; __g
EU508311	9.91150442	0.00704278	0.06579326	1.8	0	0	Bacteria; __Tenericutes; __Mollicutes; __RF9; __f; __g
EU454200	9.88235294	0.00714619	0.06579326	178.4	0	0	Bacteria; __Tenericutes; __Mollicutes; __RF9; __f; __g
EU455880	9.14165067	0.01034941	0.08233277	11.2	18	0	Bacteria; __Tenericutes; __Mollicutes; __RF9; __f; __g

*Graphically represented in Supplementary Figure 2e
